# High-precision measurement of the W boson mass with the CMS experiment

**DOI:** 10.1038/s41586-026-10168-5

**Published:** 2026-04-08

**Authors:** The CMS Collaboration, V. Chekhovsky, A. Hayrapetyan, V. Makarenko, A. Tumasyan, W. Adam, J. W. Andrejkovic, L. Benato, T. Bergauer, S. Chatterjee, K. Damanakis, M. Dragicevic, P. S. Hussain, M. Jeitler, N. Krammer, A. Li, D. Liko, I. Mikulec, J. Schieck, R. Schöfbeck, D. Schwarz, M. Sonawane, W. Waltenberger, C.-E. Wulz, T. Janssen, H. Kwon, T. Van Laer, P. Van Mechelen, N. Breugelmans, J. D’Hondt, S. Dansana, A. De Moor, M. Delcourt, F. Heyen, Y. Hong, S. Lowette, I. Makarenko, D. Müller, S. Tavernier, M. Tytgat, G. P. Van Onsem, S. Van Putte, D. Vannerom, B. Bilin, B. Clerbaux, A. K. Das, I. De Bruyn, G. De Lentdecker, H. Evard, L. Favart, P. Gianneios, A. Khalilzadeh, F. A. Khan, K. Lee, A. Malara, M. A. Shahzad, L. Thomas, M. Vanden Bemden, C. Vander Velde, P. Vanlaer, M. De Coen, D. Dobur, G. Gokbulut, J. Knolle, L. Lambrecht, D. Marckx, K. Skovpen, N. Van Den Bossche, J. van der Linden, J. Vandenbroeck, L. Wezenbeek, S. Bein, A. Benecke, A. Bethani, G. Bruno, C. Caputo, J. De Favereau De Jeneret, C. Delaere, I. S. Donertas, A. Giammanco, A. O. Guzel, S. A. Jain, V. Lemaitre, J. Lidrych, P. Mastrapasqua, T. T. Tran, S. Turkcapar, G. A. Alves, E. Coelho, G. Correia Silva, C. Hensel, T. Menezes De Oliveira, C. Mora Herrera, P. Rebello Teles, M. Soeiro, E. J. Tonelli Manganote, A. Vilela Pereira, W. L. Aldá Júnior, M. Barroso Ferreira Filho, H. Brandao Malbouisson, W. Carvalho, J. Chinellato, E. M. Da Costa, G. G. Da Silveira, D. De Jesus Damiao, S. Fonseca De Souza, R. Gomes De Souza, T. Laux Kuhn, M. Macedo, J. Martins, K. Mota Amarilo, L. Mundim, H. Nogima, J. P. Pinheiro, A. Santoro, A. Sznajder, M. Thiel, C. A. Bernardes, L. Calligaris, T. R. Fernandez Perez Tomei, E. M. Gregores, I. Maietto Silverio, P. G. Mercadante, S. F. Novaes, B. Orzari, Sandra S. Padula, V. Scheurer, A. Aleksandrov, G. Antchev, R. Hadjiiska, P. Iaydjiev, M. Misheva, M. Shopova, G. Sultanov, A. Dimitrov, L. Litov, B. Pavlov, P. Petkov, A. Petrov, E. Shumka, S. Keshri, D. Laroze, S. Thakur, T. Cheng, T. Javaid, L. Yuan, Z. Hu, Z. Liang, J. Liu, G. M. Chen, H. S. Chen, M. Chen, F. Iemmi, C. H. Jiang, A. Kapoor, H. Liao, Z.-A. Liu, R. Sharma, J. N. Song, J. Tao, C. Wang, J. Wang, Z. Wang, H. Zhang, J. Zhao, A. Agapitos, Y. Ban, A. Carvalho Antunes De Oliveira, S. Deng, B. Guo, C. Jiang, A. Levin, C. Li, Q. Li, Y. Mao, S. Qian, S. J. Qian, X. Qin, X. Sun, D. Wang, H. Yang, Y. Zhao, C. Zhou, S. Yang, Z. You, K. Jaffel, N. Lu, G. Bauer, B. Li, H. Wang, K. Yi, J. Zhang, Y. Li, Z. Lin, C. Lu, M. Xiao, C. Avila, D. A. Barbosa Trujillo, A. Cabrera, C. Florez, J. Fraga, J. A. Reyes Vega, J. Jaramillo, C. Rendón, M. Rodriguez, A. A. Ruales Barbosa, J. D. Ruiz Alvarez, D. Giljanovic, N. Godinovic, D. Lelas, A. Sculac, M. Kovac, A. Petkovic, T. Sculac, P. Bargassa, V. Brigljevic, B. K. Chitroda, D. Ferencek, K. Jakovcic, A. Starodumov, T. Susa, A. Attikis, K. Christoforou, A. Hadjiagapiou, C. Leonidou, J. Mousa, C. Nicolaou, L. Paizanos, F. Ptochos, P. A. Razis, H. Rykaczewski, H. Saka, A. Stepennov, M. Finger, M. Finger, A. Kveton, E. Ayala, E. Carrera Jarrin, B. El-mahdy, S. Khalil, E. Salama, M. Abdullah Al-Mashad, M. A. Mahmoud, K. Ehataht, M. Kadastik, T. Lange, C. Nielsen, J. Pata, M. Raidal, L. Tani, C. Veelken, K. Osterberg, M. Voutilainen, N. Bin Norjoharuddeen, E. Brücken, F. Garcia, P. Inkaew, K. T. S. Kallonen, T. Lampén, K. Lassila-Perini, S. Lehti, T. Lindén, M. Myllymäki, M. M. Rantanen, J. Tuominiemi, H. Kirschenmann, P. Luukka, H. Petrow, M. Besancon, F. Couderc, M. Dejardin, D. Denegri, J. L. Faure, F. Ferri, S. Ganjour, P. Gras, G. Hamel de Monchenault, M. Kumar, V. Lohezic, J. Malcles, F. Orlandi, L. Portales, A. Rosowsky, M. Ö. Sahin, A. Savoy-Navarro, P. Simkina, M. Titov, M. Tornago, F. Beaudette, G. Boldrini, P. Busson, A. Cappati, C. Charlot, M. Chiusi, T. D. Cuisset, F. Damas, O. Davignon, A. De Wit, I. T. Ehle, B. A. Fontana Santos Alves, S. Ghosh, A. Gilbert, R. Granier de Cassagnac, B. Harikrishnan, L. Kalipoliti, G. Liu, M. Nguyen, S. Obraztsov, C. Ochando, R. Salerno, J. B. Sauvan, Y. Sirois, G. Sokmen, L. Urda Gómez, E. Vernazza, A. Zabi, A. Zghiche, J.-L. Agram, J. Andrea, D. Bloch, J.-M. Brom, E. C. Chabert, C. Collard, S. Falke, U. Goerlach, R. Haeberle, A.-C. Le Bihan, M. Meena, O. Poncet, G. Saha, M. A. Sessini, P. Van Hove, P. Vaucelle, A. Di Florio, D. Amram, S. Beauceron, B. Blancon, G. Boudoul, N. Chanon, D. Contardo, P. Depasse, C. Dozen, H. El Mamouni, J. Fay, S. Gascon, M. Gouzevitch, C. Greenberg, G. Grenier, B. Ille, E. Jourd`huy, I. B. Laktineh, M. Lethuillier, L. Mirabito, S. Perries, A. Purohit, M. Vander Donckt, P. Verdier, J. Xiao, G. Adamov, I. Lomidze, Z. Tsamalaidze, V. Botta, S. Consuegra Rodríguez, L. Feld, K. Klein, M. Lipinski, D. Meuser, A. Pauls, D. Pérez Adán, N. Röwert, M. Teroerde, S. Diekmann, A. Dodonova, N. Eich, D. Eliseev, F. Engelke, J. Erdmann, M. Erdmann, B. Fischer, T. Hebbeker, K. Hoepfner, F. Ivone, A. Jung, M. Y. Lee, F. Mausolf, M. Merschmeyer, A. Meyer, S. Mukherjee, F. Nowotny, A. Pozdnyakov, Y. Rath, W. Redjeb, F. Rehm, H. Reithler, V. Sarkisovi, A. Schmidt, C. Seth, A. Sharma, J. L. Spah, F. Torres Da Silva De Araujo, S. Wiedenbeck, S. Zaleski, C. Dziwok, G. Flügge, T. Kress, A. Nowack, O. Pooth, A. Stahl, T. Ziemons, A. Zotz, H. Aarup Petersen, M. Aldaya Martin, J. Alimena, S. Amoroso, Y. An, J. Bach, S. Baxter, M. Bayatmakou, H. Becerril Gonzalez, O. Behnke, A. Belvedere, F. Blekman, K. Borras, A. Campbell, A. Cardini, F. Colombina, M. De Silva, G. Eckerlin, D. Eckstein, L. I. Estevez Banos, E. Gallo, A. Geiser, V. Guglielmi, M. Guthoff, A. Hinzmann, L. Jeppe, B. Kaech, M. Kasemann, C. Kleinwort, R. Kogler, M. Komm, D. Krücker, W. Lange, D. Leyva Pernia, K. Lipka, W. Lohmann, F. Lorkowski, R. Mankel, I.-A. Melzer-Pellmann, M. Mendizabal Morentin, A. B. Meyer, G. Milella, K. Moral Figueroa, A. Mussgiller, L. P. Nair, J. Niedziela, A. Nürnberg, J. Park, E. Ranken, A. Raspereza, D. Rastorguev, J. Rübenach, L. Rygaard, M. Scham, S. Schnake, P. Schütze, C. Schwanenberger, D. Selivanova, K. Sharko, M. Shchedrolosiev, D. Stafford, F. Vazzoler, A. Ventura Barroso, R. Walsh, D. Wang, Q. Wang, K. Wichmann, L. Wiens, C. Wissing, Y. Yang, S. Zakharov, A. Zimermmane Castro Santos, A. Albrecht, S. Albrecht, M. Antonello, S. Bollweg, M. Bonanomi, P. Connor, K. El Morabit, Y. Fischer, E. Garutti, A. Grohsjean, J. Haller, D. Hundhausen, H. R. Jabusch, G. Kasieczka, P. Keicher, R. Klanner, W. Korcari, T. Kramer, C. C. Kuo, V. Kutzner, F. Labe, J. Lange, A. Lobanov, C. Matthies, L. Moureaux, M. Mrowietz, A. Nigamova, Y. Nissan, A. Paasch, K. J. Pena Rodriguez, T. Quadfasel, B. Raciti, M. Rieger, D. Savoiu, J. Schindler, P. Schleper, M. Schröder, J. Schwandt, M. Sommerhalder, H. Stadie, G. Steinbrück, A. Tews, B. Wiederspan, M. Wolf, S. Brommer, E. Butz, T. Chwalek, A. Dierlamm, G. G. Dincer, U. Elicabuk, N. Faltermann, M. Giffels, A. Gottmann, F. Hartmann, R. Hofsaess, M. Horzela, U. Husemann, J. Kieseler, M. Klute, O. Lavoryk, J. M. Lawhorn, M. Link, A. Lintuluoto, S. Maier, M. Mormile, TH. Müller, M. Neukum, M. Oh, E. Pfeffer, M. Presilla, G. Quast, K. Rabbertz, B. Regnery, R. Schmieder, N. Shadskiy, I. Shvetsov, H. J. Simonis, L. Sowa, L. Stockmeier, K. Tauqeer, M. Toms, B. Topko, N. Trevisani, T. Voigtländer, R. F. Von Cube, J. Von Den Driesch, M. Wassmer, S. Wieland, F. Wittig, R. Wolf, X. Zuo, G. Anagnostou, G. Daskalakis, A. Kyriakis, A. Papadopoulos, A. Stakia, G. Melachroinos, Z. Painesis, I. Paraskevas, N. Saoulidou, K. Theofilatos, E. Tziaferi, K. Vellidis, I. Zisopoulos, G. Bakas, T. Chatzistavrou, G. Karapostoli, K. Kousouris, I. Papakrivopoulos, E. Siamarkou, G. Tsipolitis, I. Bestintzanos, I. Evangelou, C. Foudas, C. Kamtsikis, P. Katsoulis, P. Kokkas, P. G. Kosmoglou Kioseoglou, N. Manthos, I. Papadopoulos, J. Strologas, C. Hajdu, D. Horvath, K. Márton, A. J. Rádl, F. Sikler, V. Veszpremi, M. Csanád, K. Farkas, A. Fehérkuti, M. M. A. Gadallah, Á. Kadlecsik, P. Major, G. Pásztor, G. I. Veres, B. Ujvari, G. Zilizi, G. Bencze, S. Czellar, J. Molnar, Z. Szillasi, T. Csorgo, F. Nemes, T. Novak, S. Bansal, S. B. Beri, V. Bhatnagar, G. Chaudhary, S. Chauhan, N. Dhingra, A. Kaur, H. Kaur, M. Kaur, S. Kumar, T. Sheokand, J. B. Singh, A. Singla, A. Bhardwaj, A. Chhetri, B. C. Choudhary, A. Kumar, M. Naimuddin, K. Ranjan, M. K. Saini, S. Saumya, S. Baradia, S. Barman, S. Bhattacharya, S. Das Gupta, S. Dutta, S. Sarkar, M. M. Ameen, P. K. Behera, S. C. Behera, S. Chatterjee, G. Dash, P. Jana, P. Kalbhor, S. Kamble, J. R. Komaragiri, D. Kumar, T. Mishra, B. Parida, P. R. Pujahari, N. R. Saha, A. K. Sikdar, R. K. Singh, P. Verma, S. Verma, A. Vijay, S. Dugad, G. B. Mohanty, M. Shelake, P. Suryadevara, A. Bala, S. Banerjee, S. Bhowmik, R. M. Chatterjee, M. Guchait, S. H. Jain, A. Jaiswal, B. M. Joshi, S. Kumar, G. Majumder, K. Mazumdar, S. Parolia, A. Thachayath, S. Bahinipati, C. Kar, D. Maity, P. Mal, K. Naskar, A. Nayak, S. Nayak, K. Pal, P. Sadangi, S. K. Swain, S. Varghese, D. Vats, S. Acharya, A. Alpana, S. Dube, B. Gomber, P. Hazarika, B. Kansal, A. Laha, B. Sahu, S. Sharma, K. Y. Vaish, H. Bakhshiansohi, A. Jafari, M. Zeinali, S. Bashiri, S. Chenarani, S. M. Etesami, Y. Hosseini, M. Khakzad, E. Khazaie, M. Mohammadi Najafabadi, S. Tizchang, M. Felcini, M. Grunewald, M. Abbrescia, A. Colaleo, D. Creanza, B. D’Anzi, N. De Filippis, M. De Palma, W. Elmetenawee, N. Ferrara, L. Fiore, G. Iaselli, L. Longo, M. Louka, G. Maggi, M. Maggi, I. Margjeka, V. Mastrapasqua, S. My, S. Nuzzo, A. Pellecchia, A. Pompili, G. Pugliese, R. Radogna, D. Ramos, A. Ranieri, L. Silvestris, F. M. Simone, Ü. Sözbilir, A. Stamerra, D. Troiano, R. Venditti, P. Verwilligen, A. Zaza, G. Abbiendi, C. Battilana, D. Bonacorsi, P. Capiluppi, A. Castro, F. R. Cavallo, M. Cuffiani, G. M. Dallavalle, T. Diotalevi, F. Fabbri, A. Fanfani, D. Fasanella, P. Giacomelli, L. Giommi, C. Grandi, L. Guiducci, S. Lo Meo, M. Lorusso, L. Lunerti, S. Marcellini, G. Masetti, F. L. Navarria, G. Paggi, A. Perrotta, F. Primavera, A. M. Rossi, S. Rossi Tisbeni, T. Rovelli, G. P. Siroli, S. Costa, A. Di Mattia, A. Lapertosa, R. Potenza, A. Tricomi, P. Assiouras, G. Barbagli, G. Bardelli, B. Camaiani, A. Cassese, R. Ceccarelli, V. Ciulli, C. Civinini, R. D’Alessandro, E. Focardi, T. Kello, G. Latino, P. Lenzi, M. Lizzo, M. Meschini, S. Paoletti, A. Papanastassiou, G. Sguazzoni, L. Viliani, L. Benussi, S. Bianco, S. Meola, D. Piccolo, M. Alves Gallo Pereira, F. Ferro, E. Robutti, S. Tosi, A. Benaglia, F. Brivio, F. Cetorelli, F. De Guio, M. E. Dinardo, P. Dini, S. Gennai, R. Gerosa, A. Ghezzi, P. Govoni, L. Guzzi, G. Lavizzari, M. T. Lucchini, M. Malberti, S. Malvezzi, A. Massironi, D. Menasce, L. Moroni, M. Paganoni, S. Palluotto, D. Pedrini, A. Perego, B. S. Pinolini, G. Pizzati, S. Ragazzi, T. Tabarelli de Fatis, S. Buontempo, A. Cagnotta, F. Carnevali, N. Cavallo, F. Fabozzi, A. O. M. Iorio, L. Lista, P. Paolucci, B. Rossi, R. Ardino, P. Azzi, N. Bacchetta, D. Bisello, P. Bortignon, G. Bortolato, A. C. M. Bulla, R. Carlin, T. Dorigo, F. Gasparini, U. Gasparini, S. Giorgetti, E. Lusiani, M. Margoni, A. T. Meneguzzo, M. Migliorini, M. Passaseo, J. Pazzini, P. Ronchese, R. Rossin, M. Sgaravatto, F. Simonetto, M. Tosi, A. Triossi, S. Ventura, M. Zanetti, P. Zotto, A. Zucchetta, A. Braghieri, S. Calzaferri, D. Fiorina, P. Montagna, V. Re, C. Riccardi, P. Salvini, I. Vai, P. Vitulo, S. Ajmal, M. E. Ascioti, G. M. Bilei, C. Carrivale, D. Ciangottini, L. Fanò, V. Mariani, M. Menichelli, F. Moscatelli, A. Rossi, A. Santocchia, D. Spiga, T. Tedeschi, C. Aimè, C. A. Alexe, P. Asenov, P. Azzurri, G. Bagliesi, V. Bertacchi, R. Bhattacharya, L. Bianchini, T. Boccali, E. Bossini, D. Bruschini, R. Castaldi, M. A. Ciocci, M. Cipriani, V. D’Amante, R. Dell’Orso, S. Donato, R. Forti, A. Giassi, F. Ligabue, A. C. Marini, D. Matos Figueiredo, A. Messineo, S. Mishra, V. K. Muraleedharan Nair Bindhu, M. Musich, S. Nandan, F. Palla, A. Rizzi, G. Rolandi, S. Roy Chowdhury, T. Sarkar, A. Scribano, P. Spagnolo, F. Tenchini, R. Tenchini, G. Tonelli, N. Turini, F. Vaselli, A. Venturi, P. G. Verdini, P. Barria, C. Basile, F. Cavallari, L. Cunqueiro Mendez, D. Del Re, E. Di Marco, M. Diemoz, F. Errico, R. Gargiulo, E. Longo, L. Martikainen, J. Mijuskovic, G. Organtini, F. Pandolfi, R. Paramatti, C. Quaranta, S. Rahatlou, C. Rovelli, F. Santanastasio, L. Soffi, V. Vladimirov, N. Amapane, R. Arcidiacono, S. Argiro, M. Arneodo, N. Bartosik, R. Bellan, C. Biino, C. Borca, N. Cartiglia, M. Costa, R. Covarelli, N. Demaria, L. Finco, M. Grippo, B. Kiani, F. Legger, F. Luongo, C. Mariotti, L. Markovic, S. Maselli, A. Mecca, L. Menzio, P. Meridiani, E. Migliore, M. Monteno, R. Mulargia, M. M. Obertino, G. Ortona, L. Pacher, N. Pastrone, M. Pelliccioni, M. Ruspa, F. Siviero, V. Sola, A. Solano, A. Staiano, C. Tarricone, D. Trocino, G. Umoret, R. White, J. Babbar, S. Belforte, V. Candelise, M. Casarsa, F. Cossutti, K. De Leo, G. Della Ricca, S. Dogra, J. Hong, J. Kim, D. Lee, H. Lee, S. W. Lee, C. S. Moon, Y. D. Oh, M. S. Ryu, S. Sekmen, B. Tae, Y. C. Yang, M. S. Kim, G. Bak, P. Gwak, H. Kim, D. H. Moon, E. Asilar, J. Choi, D. Kim, T. J. Kim, J. A. Merlin, Y. Ryou, S. Choi, S. Han, B. Hong, K. Lee, K. S. Lee, S. Lee, J. Yoo, J. Goh, S. Yang, Y. Kang, H. S. Kim, Y. Kim, S. Lee, J. Almond, J. H. Bhyun, J. Choi, W. Jun, J. Kim, Y. W. Kim, S. Ko, H. Lee, J. Lee, B. H. Oh, S. B. Oh, H. Seo, U. K. Yang, I. Yoon, W. Jang, D. Y. Kang, S. Kim, B. Ko, J. S. H. Lee, Y. Lee, I. C. Park, Y. Roh, I. J. Watson, S. Ha, K. Hwang, B. Kim, H. D. Yoo, M. Choi, M. R. Kim, H. Lee, Y. Lee, I. Yu, T. Beyrouthy, Y. Gharbia, F. Alazemi, K. Dreimanis, A. Gaile, C. Munoz Diaz, D. Osite, G. Pikurs, A. Potrebko, M. Seidel, D. Sidiropoulos Kontos, N. R. Strautnieks, M. Ambrozas, A. Juodagalvis, A. Rinkevicius, G. Tamulaitis, I. Yusuff, Z. Zolkapli, J. F. Benitez, A. Castaneda Hernandez, H. A. Encinas Acosta, L. G. Gallegos Maríñz, M. León Coello, J. A. Murillo Quijada, A. Sehrawat, L. Valencia Palomo, G. Ayala, H. Castilla-Valdez, H. Crotte Ledesma, E. De La Cruz-Burelo, I. Heredia-De La Cruz, R. Lopez-Fernandez, J. Mejia Guisao, A. Sánchez Hernández, C. Oropeza Barrera, D. L. Ramirez Guadarrama, M. Ramírez García, I. Bautista, F. E. Neri Huerta, I. Pedraza, H. A. Salazar Ibarguen, C. Uribe Estrada, I. Bubanja, N. Raicevic, P. H. Butler, A. Ahmad, M. I. Asghar, A. Awais, M. I. M. Awan, H. R. Hoorani, W. A. Khan, V. Avati, A. Bellora, L. Forthomme, L. Grzanka, M. Malawski, K. Piotrzkowski, H. Bialkowska, M. Bluj, M. Górski, M. Kazana, M. Szleper, P. Zalewski, K. Bunkowski, K. Doroba, A. Kalinowski, M. Konecki, J. Krolikowski, A. Muhammad, P. Fokow, K. Pozniak, W. Zabolotny, M. Araujo, D. Bastos, C. Beirão Da Cruz E Silva, A. Boletti, M. Bozzo, T. Camporesi, G. Da Molin, P. Faccioli, M. Gallinaro, J. Hollar, N. Leonardo, G. B. Marozzo, A. Petrilli, M. Pisano, J. Seixas, J. Varela, J. W. Wulff, P. Adzic, P. Milenovic, D. Devetak, M. Dordevic, J. Milosevic, L. Nadderd, V. Rekovic, M. Stojanovic, J. Alcaraz Maestre, Cristina F. Bedoya, J. A. Brochero Cifuentes, Oliver M. Carretero, M. Cepeda, M. Cerrada, N. Colino, B. De La Cruz, A. Delgado Peris, A. Escalante Del Valle, D. Fernández Del Val, J. P. Fernández Ramos, J. Flix, M. C. Fouz, O. Gonzalez Lopez, S. Goy Lopez, J. M. Hernandez, M. I. Josa, J. Llorente Merino, C. Martin Perez, E. Martin Viscasillas, D. Moran, C. M. Morcillo Perez, Á. Navarro Tobar, C. Perez Dengra, A. Pérez-Calero Yzquierdo, J. Puerta Pelayo, I. Redondo, J. Sastre, J. Vazquez Escobar, J. F. de Trocóniz, B. Alvarez Gonzalez, J. Cuevas, J. Fernandez Menendez, S. Folgueras, I. Gonzalez Caballero, P. Leguina, E. Palencia Cortezon, J. Prado Pico, V. Rodríguez Bouza, A. Soto Rodríguez, A. Trapote, C. Vico Villalba, P. Vischia, S. Blanco Fernández, I. J. Cabrillo, A. Calderon, J. Duarte Campderros, M. Fernandez, G. Gomez, C. Lasaosa García, R. Lopez Ruiz, C. Martinez Rivero, P. Martinez Ruiz del Arbol, F. Matorras, P. Matorras Cuevas, E. Navarrete Ramos, J. Piedra Gomez, L. Scodellaro, I. Vila, J. M. Vizan Garcia, B. Kailasapathy, D. D. C. Wickramarathna, W. G. D. Dharmaratna, K. Liyanage, N. Perera, D. Abbaneo, C. Amendola, E. Auffray, J. Baechler, D. Barney, A. Bermúdez Martínez, M. Bianco, A. A. Bin Anuar, A. Bocci, L. Borgonovi, C. Botta, A. Bragagnolo, E. Brondolin, C. E. Brown, C. Caillol, G. Cerminara, N. Chernyavskaya, D. d’Enterria, A. Dabrowski, A. David, A. De Roeck, M. M. Defranchis, M. Deile, M. Dobson, M. Dünser, G. Franzoni, W. Funk, S. Giani, D. Gigi, K. Gill, F. Glege, M. Glowacki, J. Hegeman, J. K. Heikkilä, B. Huber, V. Innocente, T. James, P. Janot, O. Kaluzinska, O. Karacheban, G. Karathanasis, S. Laurila, P. Lecoq, E. Leutgeb, C. Lourenço, M. Magherini, L. Malgeri, M. Mannelli, M. Matthewman, A. Mehta, F. Meijers, S. Mersi, E. Meschi, V. Milosevic, F. Monti, F. Moortgat, M. Mulders, I. Neutelings, S. Orfanelli, F. Pantaleo, G. Petrucciani, A. Pfeiffer, M. Pierini, M. Pitt, H. Qu, D. Rabady, B. Ribeiro Lopes, F. Riti, M. Rovere, H. Sakulin, R. Salvatico, S. Sanchez Cruz, S. Scarfi, C. Schwick, M. Selvaggi, A. Sharma, K. Shchelina, P. Silva, P. Sphicas, A. G. Stahl Leiton, A. Steen, S. Summers, D. Treille, P. Tropea, D. Walter, J. Wanczyk, J. Wang, S. Wuchterl, P. Zehetner, P. Zejdl, W. D. Zeuner, T. Bevilacqua, L. Caminada, A. Ebrahimi, W. Erdmann, R. Horisberger, Q. Ingram, H. C. Kaestli, D. Kotlinski, C. Lange, M. Missiroli, L. Noehte, T. Rohe, A. Samalan, T. K. Aarrestad, M. Backhaus, G. Bonomelli, A. Calandri, C. Cazzaniga, K. Datta, P. De Bryas Dexmiers D’archiac, A. De Cosa, G. Dissertori, M. Dittmar, M. DonegÃ, F. Eble, M. Galli, K. Gedia, F. Glessgen, C. Grab, N. Härringer, T. G. Harte, D. Hits, W. Lustermann, A.-M. Lyon, R. A. Manzoni, M. Marchegiani, L. Marchese, A. Mascellani, F. Nessi-Tedaldi, F. Pauss, V. Perovic, S. Pigazzini, B. Ristic, R. Seidita, J. Steggemann, A. Tarabini, D. Valsecchi, R. Wallny, C. Amsler, P. Bärtschi, M. F. Canelli, K. Cormier, M. Huwiler, W. Jin, A. Jofrehei, B. Kilminster, S. Leontsinis, S. P. Liechti, A. Macchiolo, P. Meiring, F. Meng, J. Motta, A. Reimers, P. Robmann, M. Senger, E. Shokr, F. Stäger, R. Tramontano, C. Adloff, D. Bhowmik, C. M. Kuo, W. Lin, P. K. Rout, P. C. Tiwari, L. Ceard, K. F. Chen, Z. G. Chen, A. De Iorio, W.-S. Hou, T. H. Hsu, Y. W. Kao, S. Karmakar, G. Kole, Y. Y. Li, R.-S. Lu, E. Paganis, X. F. Su, J. Thomas-Wilsker, L. S. Tsai, D. Tsionou, H. Y. Wu, E. Yazgan, C. Asawatangtrakuldee, N. Srimanobhas, V. Wachirapusitanand, Y. Maghrbi, D. Agyel, F. Boran, F. Dolek, I. Dumanoglu, E. Eskut, Y. Guler, E. Gurpinar Guler, C. Isik, O. Kara, A. Kayis Topaksu, Y. Komurcu, G. Onengut, K. Ozdemir, A. Polatoz, B. Tali, U. G. Tok, E. Uslan, I. S. Zorbakir, M. Yalvac, B. Akgun, I. O. Atakisi, E. Gülmez, M. Kaya, O. Kaya, S. Tekten, A. Cakir, K. Cankocak, S. Sen, O. Aydilek, B. Hacisahinoglu, I. Hos, B. Kaynak, S. Ozkorucuklu, O. Potok, H. Sert, C. Simsek, C. Zorbilmez, S. Cerci, B. Isildak, D. Sunar Cerci, T. Yetkin, A. Boyaryntsev, B. Grynyov, L. Levchuk, D. Anthony, J. J. Brooke, A. Bundock, F. Bury, E. Clement, D. Cussans, H. Flacher, J. Goldstein, H. F. Heath, M.-L. Holmberg, L. Kreczko, S. Paramesvaran, L. Robertshaw, V. J. Smith, K. Walkingshaw Pass, A. H. Ball, K. W. Bell, A. Belyaev, C. Brew, R. M. Brown, D. J. A. Cockerill, C. Cooke, A. Elliot, K. V. Ellis, K. Harder, S. Harper, J. Linacre, K. Manolopoulos, D. M. Newbold, E. Olaiya, D. Petyt, T. Reis, A. R. Sahasransu, G. Salvi, T. Schuh, C. H. Shepherd-Themistocleous, I. R. Tomalin, K. C. Whalen, T. Williams, I. Andreou, R. Bainbridge, P. Bloch, O. Buchmuller, C. A. Carrillo Montoya, G. S. Chahal, D. Colling, J. S. Dancu, I. Das, P. Dauncey, G. Davies, M. Della Negra, S. Fayer, G. Fedi, G. Hall, A. Howard, G. Iles, C. R. Knight, P. Krueper, J. Langford, K. H. Law, J. León Holgado, L. Lyons, A.-M. Magnan, B. Maier, S. Mallios, M. Mieskolainen, J. Nash, M. Pesaresi, P. B. Pradeep, B. C. Radburn-Smith, A. Richards, A. Rose, K. Savva, C. Seez, R. Shukla, A. Tapper, K. Uchida, G. P. Uttley, T. Virdee, M. Vojinovic, N. Wardle, D. Winterbottom, J. E. Cole, A. Khan, P. Kyberd, I. D. Reid, S. Abdullin, A. Brinkerhoff, E. Collins, M. R. Darwish, J. Dittmann, K. Hatakeyama, V. Hegde, J. Hiltbrand, B. McMaster, J. Samudio, S. Sawant, C. Sutantawibul, J. Wilson, R. Bartek, A. Dominguez, A. E. Simsek, S. S. Yu, B. Bam, A. Buchot Perraguin, R. Chudasama, S. I. Cooper, C. Crovella, S. V. Gleyzer, E. Pearson, C. U. Perez, P. Rumerio, E. Usai, R. Yi, A. Akpinar, C. Cosby, G. De Castro, Z. Demiragli, C. Erice, C. Fangmeier, C. Fernandez Madrazo, E. Fontanesi, D. Gastler, F. Golf, S. Jeon, J. O’cain, I. Reed, J. Rohlf, K. Salyer, D. Sperka, D. Spitzbart, I. Suarez, A. Tsatsos, A. G. Zecchinelli, G. Barone, G. Benelli, D. Cutts, L. Gouskos, M. Hadley, U. Heintz, K. W. Ho, J. M. Hogan, T. Kwon, G. Landsberg, K. T. Lau, J. Luo, S. Mondal, T. Russell, S. Sagir, X. Shen, M. Stamenkovic, N. Venkatasubramanian, S. Abbott, B. Barton, C. Brainerd, R. Breedon, H. Cai, M. Calderon De La Barca Sanchez, M. Chertok, M. Citron, J. Conway, P. T. Cox, R. Erbacher, F. Jensen, O. Kukral, G. Mocellin, M. Mulhearn, S. Ostrom, W. Wei, S. Yoo, F. Zhang, K. Adamidis, M. Bachtis, D. Campos, R. Cousins, A. Datta, G. Flores Avila, J. Hauser, M. Ignatenko, M. A. Iqbal, T. Lam, Y. F. Lo, E. Manca, A. Nunez Del Prado, D. Saltzberg, V. Valuev, R. Clare, J. W. Gary, G. Hanson, A. Aportela, A. Arora, J. G. Branson, S. Cittolin, S. Cooperstein, D. Diaz, J. Duarte, L. Giannini, Y. Gu, J. Guiang, R. Kansal, V. Krutelyov, R. Lee, J. Letts, M. Masciovecchio, F. Mokhtar, S. Mukherjee, M. Pieri, D. Primosch, M. Quinnan, V. Sharma, M. Tadel, E. Vourliotis, F. Würthwein, Y. Xiang, A. Yagil, A. Barzdukas, L. Brennan, C. Campagnari, K. Downham, C. Grieco, M. M. Hussain, J. Incandela, J. Kim, A. J. Li, P. Masterson, H. Mei, J. Richman, S. N. Santpur, U. Sarica, R. Schmitz, F. Setti, J. Sheplock, D. Stuart, T. Á. Vámi, X. Yan, D. Zhang, S. Bhattacharya, A. Bornheim, O. Cerri, J. Mao, H. B. Newman, G. Reales Gutiérrez, M. Spiropulu, J. R. Vlimant, C. Wang, S. Xie, R. Y. Zhu, J. Alison, S. An, P. Bryant, M. Cremonesi, V. Dutta, T. Ferguson, T. A. Gómez Espinosa, A. Harilal, A. Kallil Tharayil, M. Kanemura, C. Liu, T. Mudholkar, S. Murthy, P. Palit, K. Park, M. Paulini, A. Roberts, A. Sanchez, W. Terrill, J. P. Cumalat, W. T. Ford, A. Hart, A. Hassani, N. Manganelli, J. Pearkes, C. Savard, N. Schonbeck, K. Stenson, K. A. Ulmer, S. R. Wagner, N. Zipper, D. Zuolo, J. Alexander, X. Chen, D. J. Cranshaw, J. Dickinson, J. Fan, X. Fan, S. Hogan, P. Kotamnives, J. Monroy, M. Oshiro, J. R. Patterson, M. Reid, A. Ryd, J. Thom, P. Wittich, R. Zou, M. Albrow, M. Alyari, O. Amram, G. Apollinari, A. Apresyan, L. A. T. Bauerdick, D. Berry, J. Berryhill, P. C. Bhat, K. Burkett, J. N. Butler, A. Canepa, G. B. Cerati, H. W. K. Cheung, F. Chlebana, G. Cummings, I. Dutta, V. D. Elvira, J. Freeman, A. Gandrakota, Z. Gecse, L. Gray, D. Green, A. Grummer, S. Grünendahl, D. Guerrero, O. Gutsche, R. M. Harris, T. C. Herwig, J. Hirschauer, B. Jayatilaka, S. Jindariani, M. Johnson, U. Joshi, T. Klijnsma, B. Klima, K. H. M. Kwok, S. Lammel, C. Lee, D. Lincoln, R. Lipton, T. Liu, K. Maeshima, D. Mason, P. McBride, P. Merkel, S. Mrenna, S. Nahn, J. Ngadiuba, D. Noonan, S. Norberg, V. Papadimitriou, N. Pastika, K. Pedro, C. Pena, F. Ravera, A. Reinsvold Hall, L. Ristori, M. Safdari, E. Sexton-Kennedy, N. Smith, A. Soha, L. Spiegel, S. Stoynev, J. Strait, L. Taylor, S. Tkaczyk, N. V. Tran, L. Uplegger, E. W. Vaandering, I. Zoi, C. Aruta, P. Avery, D. Bourilkov, P. Chang, V. Cherepanov, R. D. Field, C. Huh, E. Koenig, M. Kolosova, J. Konigsberg, A. Korytov, K. Matchev, N. Menendez, G. Mitselmakher, K. Mohrman, A. Muthirakalayil Madhu, N. Rawal, S. Rosenzweig, Y. Takahashi, J. Wang, T. Adams, A. Al Kadhim, A. Askew, S. Bower, R. Hashmi, R. S. Kim, S. Kim, T. Kolberg, G. Martinez, H. Prosper, P. R. Prova, M. Wulansatiti, R. Yohay, J. Zhang, B. Alsufyani, S. Butalla, S. Das, T. Elkafrawy, M. Hohlmann, E. Yanes, M. R. Adams, A. Baty, C. Bennett, R. Cavanaugh, R. Escobar Franco, O. Evdokimov, C. E. Gerber, M. Hawksworth, A. Hingrajiya, D. J. Hofman, J. H. Lee, D. S. Lemos, C. Mills, S. Nanda, G. Oh, B. Ozek, D. Pilipovic, R. Pradhan, E. Prifti, P. Roy, T. Roy, S. Rudrabhatla, N. Singh, M. B. Tonjes, N. Varelas, M. A. Wadud, Z. Ye, J. Yoo, M. Alhusseini, D. Blend, K. Dilsiz, L. Emediato, G. Karaman, O. K. Köseyan, J.-P. Merlo, A. Mestvirishvili, O. Neogi, H. Ogul, Y. Onel, A. Penzo, C. Snyder, E. Tiras, B. Blumenfeld, L. Corcodilos, J. Davis, A. V. Gritsan, L. Kang, S. Kyriacou, P. Maksimovic, M. Roguljic, J. Roskes, S. Sekhar, M. Swartz, A. Abreu, L. F. Alcerro Alcerro, J. Anguiano, S. Arteaga Escatel, P. Baringer, A. Bean, Z. Flowers, D. Grove, J. King, G. Krintiras, M. Lazarovits, C. Le Mahieu, J. Marquez, M. Murray, M. Nickel, S. Popescu, C. Rogan, C. Royon, S. Sanders, C. Smith, G. Wilson, B. Allmond, R. Gujju Gurunadha, A. Ivanov, K. Kaadze, Y. Maravin, J. Natoli, D. Roy, G. Sorrentino, A. Baden, A. Belloni, J. Bistany-riebman, Y. M. Chen, S. C. Eno, N. J. Hadley, S. Jabeen, R. G. Kellogg, T. Koeth, B. Kronheim, Y. Lai, S. Lascio, A. C. Mignerey, S. Nabili, C. Palmer, C. Papageorgakis, M. M. Paranjpe, E. Popova, A. Shevelev, L. Wang, L. Zhang, C. Baldenegro Barrera, J. Bendavid, S. Bright-Thonney, I. A. Cali, P. C. Chou, M. D’Alfonso, J. Eysermans, C. Freer, G. Gomez-Ceballos, M. Goncharov, G. Grosso, P. Harris, D. Hoang, D. Kovalskyi, J. Krupa, L. Lavezzo, Y.-J. Lee, K. Long, C. Mcginn, A. Novak, M. I. Park, C. Paus, C. Reissel, C. Roland, G. Roland, S. Rothman, G. S. F. Stephans, Z. Wang, B. Wyslouch, T. J. Yang, B. Crossman, C. Kapsiak, M. Krohn, D. Mahon, J. Mans, B. Marzocchi, M. Revering, R. Rusack, R. Saradhy, N. Strobbe, K. Bloom, D. R. Claes, G. Haza, J. Hossain, C. Joo, I. Kravchenko, A. Rohilla, J. E. Siado, W. Tabb, A. Vagnerini, A. Wightman, F. Yan, D. Yu, H. Bandyopadhyay, L. Hay, H. W. Hsia, I. Iashvili, A. Kalogeropoulos, A. Kharchilava, M. Morris, D. Nguyen, S. Rappoccio, H. Rejeb Sfar, A. Williams, P. Young, G. Alverson, E. Barberis, J. Bonilla, B. Bylsma, M. Campana, J. Dervan, Y. Haddad, Y. Han, I. Israr, A. Krishna, P. Levchenko, J. Li, M. Lu, R. Mccarthy, D. M. Morse, T. Orimoto, A. Parker, L. Skinnari, E. Tsai, D. Wood, S. Dittmer, K. A. Hahn, D. Li, Y. Liu, M. Mcginnis, Y. Miao, D. G. Monk, M. H. Schmitt, A. Taliercio, M. Velasco, G. Agarwal, R. Band, R. Bucci, S. Castells, A. Das, R. Goldouzian, M. Hildreth, K. Hurtado Anampa, T. Ivanov, C. Jessop, K. Lannon, J. Lawrence, N. Loukas, L. Lutton, J. Mariano, N. Marinelli, I. Mcalister, T. McCauley, C. Mcgrady, C. Moore, Y. Musienko, H. Nelson, M. Osherson, A. Piccinelli, R. Ruchti, A. Townsend, Y. Wan, M. Wayne, H. Yockey, M. Zarucki, L. Zygala, A. Basnet, M. Carrigan, L. S. Durkin, C. Hill, M. Joyce, M. Nunez Ornelas, K. Wei, D. A. Wenzl, B. L. Winer, B. R. Yates, H. Bouchamaoui, K. Coldham, P. Das, G. Dezoort, P. Elmer, P. Fackeldey, A. Frankenthal, B. Greenberg, N. Haubrich, K. Kennedy, G. Kopp, S. Kwan, D. Lange, A. Loeliger, D. Marlow, I. Ojalvo, J. Olsen, F. Simpson, D. Stickland, C. Tully, L. H. Vage, S. Malik, R. Sharma, A. S. Bakshi, S. Chandra, R. Chawla, A. Gu, L. Gutay, M. Jones, A. W. Jung, A. M. Koshy, M. Liu, G. Negro, N. Neumeister, G. Paspalaki, S. Piperov, J. F. Schulte, A. K. Virdi, F. Wang, A. Wildridge, W. Xie, Y. Yao, J. Dolen, N. Parashar, A. Pathak, D. Acosta, A. Agrawal, T. Carnahan, K. M. Ecklund, P. J. Fernández Manteca, S. Freed, P. Gardner, F. J. M. Geurts, I. Krommydas, W. Li, J. Lin, O. Miguel Colin, B. P. Padley, R. Redjimi, J. Rotter, E. Yigitbasi, Y. Zhang, A. Bodek, P. de Barbaro, R. Demina, J. L. Dulemba, A. Garcia-Bellido, O. Hindrichs, A. Khukhunaishvili, N. Parmar, P. Parygin, R. Taus, B. Chiarito, J. P. Chou, S. V. Clark, D. Gadkari, Y. Gershtein, E. Halkiadakis, M. Heindl, C. Houghton, D. Jaroslawski, S. Konstantinou, I. Laflotte, A. Lath, R. Montalvo, K. Nash, J. Reichert, P. Saha, S. Salur, S. Schnetzer, S. Somalwar, R. Stone, S. A. Thayil, S. Thomas, J. Vora, D. Ally, A. G. Delannoy, S. Fiorendi, S. Higginbotham, T. Holmes, A. R. Kanuganti, N. Karunarathna, L. Lee, E. Nibigira, S. Spanier, D. Aebi, M. Ahmad, T. Akhter, K. Androsov, O. Bouhali, R. Eusebi, J. Gilmore, T. Huang, T. Kamon, H. Kim, S. Luo, R. Mueller, D. Overton, A. Safonov, N. Akchurin, J. Damgov, Y. Feng, N. Gogate, Y. Kazhykarim, K. Lamichhane, S. W. Lee, C. Madrid, A. Mankel, T. Peltola, I. Volobouev, E. Appelt, Y. Chen, S. Greene, A. Gurrola, W. Johns, R. Kunnawalkam Elayavalli, A. Melo, D. Rathjens, F. Romeo, P. Sheldon, S. Tuo, J. Velkovska, J. Viinikainen, B. Cardwell, H. Chung, B. Cox, J. Hakala, R. Hirosky, A. Ledovskoy, C. Mantilla, C. Neu, C. Ramón Álvarez, S. Bhattacharya, P. E. Karchin, A. Aravind, S. Banerjee, K. Black, T. Bose, E. Chavez, S. Dasu, P. Everaerts, C. Galloni, H. He, M. Herndon, A. Herve, C. K. Koraka, A. Lanaro, R. Loveless, J. Madhusudanan Sreekala, A. Mallampalli, A. Mohammadi, S. Mondal, G. Parida, L. Pétré, D. Pinna, A. Savin, V. Shang, V. Sharma, W. H. Smith, D. Teague, H. F. Tsoi, W. Vetens, A. Warden, S. Afanasiev, V. Alexakhin, D. Budkouski, I. Golutvin, I. Gorbunov, V. Karjavine, O. Kodolova, V. Korenkov, A. Lanev, A. Malakhov, V. Matveev, A. Nikitenko, V. Palichik, V. Perelygin, M. Savina, V. Shalaev, S. Shmatov, S. Shulha, V. Smirnov, O. Teryaev, N. Voytishin, B. S. Yuldashev, A. Zarubin, I. Zhizhin, G. Gavrilov, V. Golovtcov, Y. Ivanov, V. Kim, V. Murzin, V. Oreshkin, D. Sosnov, V. Sulimov, L. Uvarov, A. Vorobyev, Yu. Andreev, A. Dermenev, S. Gninenko, N. Golubev, A. Karneyeu, D. Kirpichnikov, M. Kirsanov, N. Krasnikov, I. Tlisova, A. Toropin, T. Aushev, K. Ivanov, V. Gavrilov, N. Lychkovskaya, V. Popov, A. Zhokin, R. Chistov, M. Danilov, S. Polikarpov, V. Andreev, M. Azarkin, M. Kirakosyan, A. Terkulov, E. Boos, V. Bunichev, M. Dubinin, L. Dudko, V. Klyukhin, O. Lukina, M. Perfilov, V. Savrin, P. Volkov, G. Vorotnikov, V. Blinov, T. Dimova, A. Kozyrev, O. Radchenko, Y. Skovpen, V. Kachanov, S. Slabospitskii, A. Uzunian, A. Babaev, V. Borshch, D. Druzhkin

**Affiliations:** 1 Yerevan Physics Institute, Yerevan, Armenia; 2 Institut für Hochenergiephysik, Vienna, Austria; 3 Universiteit Antwerpen, Antwerpen, Belgium; 4 Vrije Universiteit Brussel, Brussels, Belgium; 5 Ghent University, Ghent, Belgium; 6 Université Libre de Bruxelles, Brussels, Belgium; 7 Université Catholique de Louvain, Louvain-la-Neuve, Belgium; 8 Centro Brasileiro de Pesquisas Fisicas, Rio de Janeiro, Brazil; 9 Universidade do Estado do Rio de Janeiro, Rio de Janeiro, Brazil; 10 Universidade Estadual Paulista, Universidade Federal do ABC, São Paulo, Brazil; 11 Institute for Nuclear Research and Nuclear Energy, Bulgarian Academy of Sciences, Sofia, Bulgaria; 12 University of Sofia, Sofia, Bulgaria; 13 Instituto De Alta Investigació, Universidad de Tarapacá, Arica, Chile; 14 Beihang University, Beijing, China; 15 Department of Physics, Tsinghua University, Beijing, China; 16 Institute of High Energy Physics, Beijing, China; 17 State Key Laboratory of Nuclear Physics and Technology, Peking University, Beijing, China; 18 Guangdong Provincial Key Laboratory of Nuclear Science and Guangdong-Hong Kong Joint Laboratory of Quantum Matter, South China Normal University, Guangzhou, China; 19 Sun Yat-Sen University, Guangzhou, China; 20 University of Science and Technology of China, Hefei, China; 21 Nanjing Normal University, Nanjing, China; 22 The University of Iowa, Iowa City, IA USA; 23 Institute of Modern Physics and Key Laboratory of Nuclear Physics and Ion-beam Application (MOE), Fudan University, Shanghai, China; 24 Zhejiang University, Hangzhou, China; 25 Universidad de Los Andes, Bogota, Colombia; 26 Universidad de Antioquia, Medellin, Colombia; 27 Faculty of Electrical Engineering, Mechanical Engineering and Naval Architecture, University of Split, Split, Croatia; 28 Faculty of Science, University of Split, Split, Croatia; 29 Institute Rudjer Boskovic, Zagreb, Croatia; 30 University of Cyprus, Nicosia, Cyprus; 31 Charles University, Prague, Czech Republic; 32 Escuela Politecnica Nacional, Quito, Ecuador; 33 Universidad San Francisco de Quito, Quito, Ecuador; 34 Egyptian Network of High Energy Physics, Academy of Scientific Research and Technology of the Arab Republic of Egypt, Cairo, Egypt; 35 Center for High Energy Physics (CHEP-FU), Fayoum University, Faiyum, Egypt; 36 National Institute of Chemical Physics and Biophysics, Tallinn, Estonia; 37 Department of Physics, University of Helsinki, Helsinki, Finland; 38 Helsinki Institute of Physics, Helsinki, Finland; 39 Lappeenranta-Lahti University of Technology, Lappeenranta, Finland; 40 IRFU, CEA, Université Paris-Saclay, Gif-sur-Yvette, France; 41 Purdue University, West Lafayette, IN USA; 42 Laboratoire Leprince-Ringuet, CNRS/IN2P3, Ecole Polytechnique, Institut Polytechnique de Paris, Palaiseau, France; 43 Université de Strasbourg, CNRS, IPHC UMR 7178, Strasbourg, France; 44 Centre de Calcul de l’Institut National de Physique Nucleaire et de Physique des Particules, CNRS/IN2P3, Villeurbanne, France; 45 Institut de Physique des 2 Infinis de Lyon (IP2I), Villeurbanne, France; 46 Georgian Technical University, Tbilisi, Georgia; 47 I. Physikalisches Institut, RWTH Aachen University, Aachen, Germany; 48 III. Physikalisches Institut A, RWTH Aachen University, Aachen, Germany; 49 III. Physikalisches Institut B, RWTH Aachen University, Aachen, Germany; 50 Deutsches Elektronen-Synchrotron, Hamburg, Germany; 51 University of Hamburg, Hamburg, Germany; 52 Karlsruher Institut fuer Technologie, Karlsruhe, Germany; 53 European Organization for Nuclear Research, CERN, Geneva, Switzerland; 54 Institute of Nuclear and Particle Physics (INPP), NCSR Demokritos, Aghia Paraskevi, Greece; 55 National and Kapodistrian University of Athens, Athens, Greece; 56 National Technical University of Athens, Athens, Greece; 57 University of Ioánnina, Ioánnina, Greece; 58 HUN-REN Wigner Research Centre for Physics, Budapest, Hungary; 59 HUN-REN ATOMKI - Institute of Nuclear Research, Debrecen, Hungary; 60 MTA-ELTE Lendület CMS Particle and Nuclear Physics Group, Eötvös Loránd University, Budapest, Hungary; 61 Faculty of Informatics, University of Debrecen, Debrecen, Hungary; 62 Karoly Robert Campus, MATE Institute of Technology, Gyongyos, Hungary; 63 Panjab University, Chandigarh, India; 64 University of Delhi, Delhi, India; 65 Saha Institute of Nuclear Physics, HBNI, Kolkata, India; 66 Indian Institute of Technology Madras, Madras, India; 67 Tata Institute of Fundamental Research-A, Mumbai, India; 68 Tata Institute of Fundamental Research-B, Mumbai, India; 69 National Institute of Science Education and Research, Homi Bhabha National Institute, Bhubaneswar, India; 70 Indian Institute of Science Education and Research (IISER), Pune, India; 71 Isfahan University of Technology, Isfahan, Iran; 72 Institute for Research in Fundamental Sciences (IPM), Tehran, Iran; 73 University College Dublin, Dublin, Ireland; 74 INFN Sezione di Bari, Bari, Italy; 75 Università di Bari, Bari, Italy; 76 Politecnico di Bari, Bari, Italy; 77 INFN Sezione di Bologna, Bologna, Italy; 78 Università di Bologna, Bologna, Italy; 79 INFN Sezione di Catania, Catania, Italy; 80 Università di Catania, Catania, Italy; 81 INFN Sezione di Firenze, Firenze, Italy; 82 Università di Firenze, Firenze, Italy; 83 INFN Laboratori Nazionali di Frascati, Frascati, Italy; 84 INFN Sezione di Genova, Genova, Italy; 85 Università di Genova, Genova, Italy; 86 INFN Sezione di Milano-Bicocca, Milan, Italy; 87 Università di Milano-Bicocca, Milan, Italy; 88 INFN Sezione di Napoli, Napoli, Italy; 89 Università di Napoli ‘Federico II’, Napoli, Italy; 90 Università della Basilicata, Potenza, Italy; 91 INFN Sezione di Padova, Padova, Italy; 92 Fermi National Accelerator Laboratory, Batavia, IL USA; 93 Università di Padova, Padova, Italy; 94 INFN Sezione di Pavia, Pavia, Italy; 95 Università di Pavia, Pavia, Italy; 96 INFN Sezione di Perugia, Perugia, Italy; 97 Università di Perugia, Perugia, Italy; 98 INFN Sezione di Pisa, Pisa, Italy; 99 Università di Pisa, Pisa, Italy; 100 Scuola Normale Superiore di Pisa, Pisa, Italy; 101 Università di Siena, Siena, Italy; 102 INFN Sezione di Roma, Rome, Italy; 103 Sapienza Università di Roma, Rome, Italy; 104 INFN Sezione di Torino, Turin, Italy; 105 Università di Torino, Turin, Italy; 106 Università del Piemonte Orientale, Novara, Italy; 107 INFN Sezione di Trieste, Trieste, Italy; 108 Università di Trieste, Trieste, Italy; 109 Kyungpook National University, Daegu, Korea; 110 Department of Mathematics and Physics - GWNU, Gangneung, Korea; 111 Institute for Universe and Elementary Particles, Chonnam National University, Gwangju, Korea; 112 Hanyang University, Seoul, Korea; 113 Korea University, Seoul, Korea; 114 Department of Physics, Kyung Hee University, Seoul, Korea; 115 Sejong University, Seoul, Korea; 116 Seoul National University, Seoul, Korea; 117 University of Seoul, Seoul, Korea; 118 Department of Physics, Yonsei University, Seoul, Korea; 119 Sungkyunkwan University, Suwon, Korea; 120 College of Engineering and Technology, American, University of the Middle East (AUM), Dasman, Kuwait; 121 Kuwait University - College of Science - Department of Physics, Safat, Kuwait; 122 Riga Technical University, Riga, Latvia; 123 University of Latvia (LU), Riga, Latvia; 124 Vilnius University, Vilnius, Lithuania; 125 National Centre for Particle Physics, Universiti Malaya, Kuala Lumpur, Malaysia; 126 Universidad de Sonora (UNISON), Hermosillo, Mexico; 127 Centro de Investigacion y de Estudios Avanzados del IPN, Mexico City, Mexico; 128 Universidad Iberoamericana, Mexico City, Mexico; 129 Benemerita Universidad Autonoma de Puebla, Puebla, Mexico; 130 University of Montenegro, Podgorica, Montenegro; 131 University of Canterbury, Christchurch, New Zealand; 132 National Centre for Physics, Quaid-i-Azam University, Islamabad, Pakistan; 133 AGH University of Krakow, Krakow, Poland; 134 National Centre for Nuclear Research, Swierk, Poland; 135 Institute of Experimental Physics, Faculty of Physics, University of Warsaw, Warsaw, Poland; 136 Warsaw University of Technology, Warsaw, Poland; 137 Laboratório de Instrumentação e Física Experimental de Partículas, Lisbon, Portugal; 138 Faculty of Physics, University of Belgrade, Belgrade, Serbia; 139 VINCA Institute of Nuclear Sciences, University of Belgrade, Belgrade, Serbia; 140 Centro de Investigaciones Energéticas Medioambientales y Tecnológicas (CIEMAT), Madrid, Spain; 141 Universidad Autónoma de Madrid, Madrid, Spain; 142 Instituto Universitario de Ciencias y Tecnologías Espaciales de Asturias (ICTEA), Universidad de Oviedo, Oviedo, Spain; 143 Instituto de Física de Cantabria (IFCA), CSIC-Universidad de Cantabria, Santander, Spain; 144 University of Colombo, Colombo, Sri Lanka; 145 Department of Physics, University of Ruhuna, Matara, Sri Lanka; 146 PSI Center for Neutron and Muon Sciences, Villigen, Switzerland; 147 Universität Zürich, Zurich, Switzerland; 148 ETH Zurich - Institute for Particle Physics and Astrophysics (IPA), Zurich, Switzerland; 149 National Central University, Chung-Li, Taiwan; 150 National Taiwan University (NTU), Taipei, Taiwan; 151 High Energy Physics Research Unit, Department of Physics, Faculty of Science, Chulalongkorn University, Bangkok, Thailand; 152 Tunis El Manar University, Tunis, Tunisia; 153 Faculty of Science and Letters, Physics Department, Çukurova University, Adana, Turkey; 154 Physics Department, Middle East Technical University, Ankara, Turkey; 155 Bogazici University, Istanbul, Turkey; 156 Istanbul Technical University, Istanbul, Turkey; 157 Istanbul University, Istanbul, Turkey; 158 Yildiz Technical University, Istanbul, Turkey; 159 Institute for Scintillation Materials of National Academy of Science of Ukraine, Kharkiv, Ukraine; 160 National Science Centre, Kharkiv Institute of Physics and Technology, Kharkiv, Ukraine; 161 University of Bristol, Bristol, UK; 162 Rutherford Appleton Laboratory, Didcot, UK; 163 Imperial College, London, UK; 164 Brunel University, Uxbridge, UK; 165 Baylor University, Waco, TX USA; 166 Catholic University of America, Washington, DC USA; 167 The University of Alabama, Tuscaloosa, AL USA; 168 Boston University, Boston, MA, USA; 169 Brown University, Providence, RI USA; 170 University of California, Davis, CA USA; 171 University of California, Los Angeles, CA, USA; 172 University of California, Riverside, CA USA; 173 University of California, San Diego, La Jolla, CA, USA; 174 Department of Physics, University of California - Santa Barbara, Santa Barbara, CA USA; 175 California Institute of Technology, Pasadena, CA USA; 176 Carnegie Mellon University, Pittsburgh, PA USA; 177 University of Colorado Boulder, Boulder, CO, USA; 178 Cornell University, Ithaca, NY, USA; 179 University of Florida, Gainesville, FL USA; 180 Florida State University, Tallahassee, FL USA; 181 Florida Institute of Technology, Melbourne, FL USA; 182 University of Illinois Chicago, Chicago, IL USA; 183 Johns Hopkins University, Baltimore, MD USA; 184 The University of Kansas, Lawrence, KS USA; 185 Kansas State University, Manhattan, KS USA; 186 University of Maryland, College Park, MD USA; 187 Massachusetts Institute of Technology, Cambridge, MA USA; 188 University of Minnesota, Minneapolis, MN USA; 189 University of Nebraska-Lincoln, Lincoln, NE USA; 190 State University of New York at Buffalo, Buffalo, NY, USA; 191 Northeastern University, Boston, MA, USA; 192 Northwestern University, Evanston, IL USA; 193 University of Notre Dame, Notre Dame, IN, USA; 194 The Ohio State University, Columbus, OH, USA; 195 Princeton University, Princeton, NJ USA; 196 University of Puerto Rico, Mayaguez, PR USA; 197 Purdue University Northwest, Hammond, IN, USA; 198 Rice University, Houston, TX USA; 199 University of Rochester, Rochester, NY, USA; 200 Rutgers, The State University of New Jersey, Piscataway, NJ, USA; 201 University of Tennessee, Knoxville, TN USA; 202 Texas A&M University, College Station, TX USA; 203 Texas Tech University, Lubbock, TX USA; 204 Vanderbilt University, Nashville, TN USA; 205 University of Virginia, Charlottesville, VA USA; 206 Wayne State University, Detroit, MI USA; 207 University of Wisconsin - Madison, Madison, WI USA; 208 An institute or international laboratory covered by a cooperation agreement with CERN, Geneva, Switzerland

**Keywords:** Experimental particle physics

## Abstract

In the standard model of particle physics, the masses of the W and Z bosons, the carriers of the weak interaction, are uniquely related. A precise determination of their masses is important because quantum loops of heavy, undiscovered particles could modify this relationship. Although the Z mass is known to the remarkable precision of 22 parts per million (2.0 MeV), the W mass is known much less precisely. A global fit to measured electroweak observables predicts the W mass with 6 MeV uncertainty^1–3^. Reaching a comparable experimental precision would be a sensitive and fundamental test of the standard model, made even more urgent by a recent challenge to the global fit prediction by a measurement from the CDF Collaboration at the Fermilab Tevatron collider^4^. Here we report the measurement of the W mass by the CMS Collaboration at the CERN Large Hadron Collider, based on a large data sample of W → μν events collected in 2016 at the proton–proton collision energy of 13 TeV. The measurement exploits a high-granularity maximum likelihood fit to the kinematic properties of muons produced in W decays. By combining an accurate determination of experimental effects with marked in situ constraints of theoretical inputs, we reach a precise measurement of the W mass, of 80,360.2 ± 9.9 MeV, in agreement with the standard model prediction.

## Abstract (web summary)

The CMS experiment at CERN reports one of the highest-precision measurements of the W boson mass, finding it in line with standard model predictions and at odds with recent anomalous measurements.

## Main

Precision measurements of fundamental parameters have played a major part in the development of the standard model (SM) of particle physics, which provides an accurate description of the known elementary particles and their interactions. Over the span of several decades, they provided increasingly precise estimates for the masses of the W and Z bosons, top quark and Higgs boson, which helped guide the experimental programmes aimed at their discoveries. With the observation of the Higgs boson at the CERN^5–7^ Large Hadron Collider (LHC) and the determination of its mass, all the parameters in the electroweak (EW) sector of the SM are now constrained by experimental measurements. Nevertheless, the SM is widely believed to be incomplete, given that it does not explain certain fundamental observations, such as the asymmetry of matter and antimatter in the universe and the existence of dark matter. In the SM, the W and Z boson masses, *m*_W_ and *m*_Z_, are uniquely related to the coupling strengths of the weak and electromagnetic interactions. If the measured masses and couplings deviated from the predicted relation, it would be a clear sign of physics beyond the SM, probably because of new particles that, although too heavy to be directly produced at existing accelerators, interact by quantum loops with the W and Z bosons^8,9^.

Following the observations of the W and Z bosons at the CERN Super Proton–Antiproton Synchrotron (${\rm{S}}{\rm{p}}\bar{{\rm{p}}}{\rm{S}}$) collider^10–13^, *m*_Z_ was measured with the exceptional precision of 22 parts per million (*m*_Z_ = 91,188.0 ± 2.0 MeV; ref. ^1^), predominantly by the experiments operating at the CERN Large Electron Positron (LEP) collider through measurements of resonant Z boson production in precise beam energy scans^14^. The *m*_W_ measurement at LEP^15^ was based on the direct reconstruction of pair-produced W bosons, the production rate of which in electron–positron collisions is several orders of magnitude lower than the Z boson production rate. Consequently, the uncertainty in the *m*_W_ measurement was an order of magnitude larger than that of *m*_Z_. Subsequent measurements performed at the Fermilab Tevatron^16^ and the LHC^17–19^ contributed to the present experimental average of *m*_W_ = 80,369.2 ± 13.3 MeV (refs. ^1,20^). The value of *m*_W_ derived from the predicted relationships of EW parameters in the SM and independently measured observables, known as a global EW fit, *m*_W_ = 80,353 ± 6 MeV (refs. ^1–3^), is significantly more precise. As such, improving the direct measurement of *m*_W_ tests the SM and enhances sensitivity to new physics. The experimental combination does not include the most precise single measurement, performed by the CDF Collaboration, *m*_W_ = 80,433.5 ± 9.4 MeV (ref. ^4^). The strong disagreement between this value and both the SM expectation and the other measurements^20^ represents an important puzzle in the field of particle physics. An independent high-precision *m*_W_ measurement is, therefore, of the utmost importance. Here we report the results of the first W boson mass determination by the CMS Collaboration. Our measurement is based on the analysis of more than 100 million reconstructed W boson decays selected from a sample of proton–proton (pp) collisions—the largest sample used for measuring *m*_W_. Together with an accurate determination of the experimental effects, this large dataset allows us to markedly reduce the theoretical and experimental uncertainties in our measurement. This result constitutes a substantial step towards resolving the W boson mass puzzle.

## Analysis strategy

At hadron colliders, jets from the hadronization of the quark–antiquark pair produced in the decay of the W boson cannot be selected and calibrated with sufficient accuracy for a precise *m*_W_ measurement. Therefore, measurements of *m*_W_ rely on the W boson decay to a charged lepton ℓ and a neutrino ν, W → *ℓ*ν, in which the W boson cannot be fully reconstructed because neutrinos are not directly measurable in collider detectors. In the rest frame of the decaying W boson, the mass of the W boson is equally shared between the momenta of the neutrino and of the charged lepton. In the laboratory frame, the transverse components of the charged lepton and neutrino momenta (${p}_{{\rm{T}}}^{{\ell }}$ and ${p}_{{\rm{T}}}^{{\rm{\nu }}}$) exhibit characteristic peaks at around *m*_W_/2, although their exact distributions depend on the transverse momentum of the W boson itself, ${p}_{{\rm{T}}}^{{\rm{W}}}$. Therefore, *m*_W_ can be indirectly measured through ${p}_{{\rm{T}}}^{{\ell }}$ or by the transverse component of the negative vector momentum sum of all measured particles in the event, ${{\bf{p}}}_{{\rm{T}}}^{\mathrm{miss}}$, an estimator of ${p}_{{\rm{T}}}^{{\boldsymbol{\nu }}}$. The ${p}_{{\rm{T}}}^{{\rm{miss}}}$ (magnitude of ${{\bf{p}}}_{{\rm{T}}}^{\mathrm{miss}}$) and the transverse mass ${m}_{{\rm{T}}}^{\,{\rm{W}}}$, defined in analogy to the two-body mass as ${m}_{{\rm{T}}}^{{\rm{W}}}=\sqrt{2\,{p}_{{\rm{T}}}^{{\ell }}\,{p}_{{\rm{T}}}^{\mathrm{miss}}-{{\bf{p}}}_{{\rm{T}}}^{{\ell }}\cdot {{\bf{p}}}_{{\rm{T}}}^{\mathrm{miss}}}$, are powerful observables in the Tevatron measurements^4,16^. However, their sensitivity to *m*_W_ in LHC measurements^17–19^ is weakened by the ${p}_{{\rm{T}}}^{{\rm{miss}}}$ resolution, which degrades in the presence of a large number of pp collisions in the same or adjacent bunch crossings (pileup). Although these channels can provide important cross-checks, these considerations inform our *m*_W_ determination strategy, which focuses on the kinematic distributions of the charged lepton in W → *ℓ*ν events.

Among the three leptonic decays, we exploit the muon (μ) channel, because it offers the best experimental precision with the multipurpose, nearly hermetic CMS detector^21^. The CMS apparatus^21^ is designed to trigger on^22–24^ and identify electrons, muons, photons and (charged and neutral) hadrons^25–27^. A global event reconstruction algorithm^28^ aims to reconstruct all individual particles in an event, combining information provided by the all-silicon inner tracker and by the crystal electromagnetic and brass-scintillator hadron calorimeters, operating inside a 3.8 T superconducting solenoid, with data from the gas-ionization muon detectors embedded in the flux-return yoke outside the solenoid. Charged-particle trajectories (tracks) are built from energy deposits in each layer of the silicon detector, referred to as ‘hits.’ Muon tracks typically have at least 12 hits, each of which is measured with an accuracy of about 15 μm in the bending plane. The muon momentum is derived from the curvature of the corresponding track, with a typical resolution for ${p}_{{\rm{T}}}^{{\rm{\mu }}}=40\,\mathrm{GeV}$ of about 1% in the central region and 4% in the forward region of the detector.

Our measurement relies on a deep understanding of both the experimental and theoretical sources of systematic uncertainty. The muon momentum scale (the largest source of uncertainty in the measurement) is calibrated to a few parts per hundred-thousand by using a sample of dimuon decays of the J/Ψ resonance. Muons from **Y**(1S) meson and Z boson decays are used for independent validations. The predicted ${p}_{{\rm{T}}}^{{\rm{\mu }}}$ distribution depends on the theoretical modelling of the ${p}_{{\rm{T}}}^{{\rm{W}}}$ distribution and on the parton distribution functions (PDFs), which describe the momentum distributions of the quarks and gluons inside the protons. The PDFs strongly affect the W boson polarization and, hence, the kinematic distributions of the decay leptons^29^. To minimize the impact of these uncertainties on our measurement, we aggregate selected data and simulated W → μν events into a highly granular three-dimensional distribution depending on ${p}_{{\rm{T}}}^{{\rm{\mu }}}$, *η*^μ^ and *q*^μ^, where ${\eta }^{{\rm{\mu }}}=-\mathrm{ln}\tan (\theta /2)$ is the muon pseudorapidity, *θ* is the muon polar angle with respect to the beam line and *q*^μ^ is the muon electric charge. This distribution is uniformly divided into 48 *η*^μ^ bins from −2.4 to 2.4, 30 ${p}_{{\rm{T}}}^{{\rm{\mu }}}$ bins from 26 GeV to 56 GeV, and two bins in *q*^μ^ (+1 or −1). The *m*_W_ value is extracted from a binned maximum likelihood fit to this distribution, using template shapes for the signal and background processes.

Our analysis uses state-of-the-art calculations to describe the W and Z boson production. The predictions combine an all-order resummation of logarithmically enhanced soft and collinear gluon emissions at next-to-next-to-next-to-leading logarithmic (N^3^LL) accuracy with next-to-next-to-leading order (NNLO) accuracy in perturbative quantum chromodynamics (QCD)^30^. The nonperturbative motion of the partons inside the proton is described by a phenomenological model^31^. We incorporate a new proposal for theory nuisance parameters (TNPs)^32^ to parameterize the impact of unknown perturbative corrections. These models, combined with uncertainty profiling^1^ in the binned maximum likelihood fit to the $({p}_{{\rm{T}}}^{{\rm{\mu }}},{\eta }^{{\rm{\mu }}},{q}^{{\rm{\mu }}})$ distribution, allow the in situ determination of the ${p}_{{\rm{T}}}^{{\rm{W}}}$ spectrum with our W → μν data and reduce its uncertainty to subleading importance in the measurement. In contrast to previous *m*_W_ measurements at hadron colliders^4,16,18,19^, we do not rely on measurements of Z boson production to modify the predicted ${p}_{{\rm{T}}}^{{\rm{W}}}$ distribution. As shown in ref. ^33^, our procedure also significantly constrains the PDFs, the second largest source of uncertainty in our *m*_W_ measurement.

We have also developed an alternative analysis approach, in which *m*_W_ is extracted simultaneously with the angular distributions of the muon from the W boson decay. This procedure is based on the general parameterization of the production cross-section of a spin-1 boson and its decay to leptons in terms of nine helicity states^34^. For each bin in the two-dimensional ${p}_{{\rm{T}}}^{{\rm{W}}}$ and W boson rapidity (*y*^W^) space, and separately for the W^+^ and W^−^ bosons, each helicity component leads to a different $({p}_{{\rm{T}}}^{{\rm{\mu }}},{\eta }^{{\rm{\mu }}})$ distribution. We perform a differential analysis, encoding the variations of the helicity components as alternative templates fitted to the $({p}_{{\rm{T}}}^{{\rm{\mu }}},{\eta }^{{\rm{\mu }}},{q}^{{\rm{\mu }}})$ distributions. Although this method, referred to as ‘helicity fit’, is less sensitive to *m*_W_, it provides a valuable cross-check of the nominal result by relaxing some assumptions about the W boson production and, hence, reducing the dependence of the measurement on theoretical predictions.

We validate the experimental and theoretical inputs of the measurement with two *m*_Z_ determinations. First, we extract *m*_Z_ through a maximum likelihood fit to the Z → μμ dimuon mass distribution. Then, we perform a W-like measurement of *m*_Z_ using only one of the two decay muons, mimicking the conditions of the *m*_W_ analysis. We model the Z and W boson production and their associated uncertainties with a common parameterization and perturbative accuracy, allowing the W-like *m*_Z_ measurement to serve as a robust validation of the predictions and uncertainties relevant for the *m*_W_ determination. The consistency of these results with the precise *m*_Z_ value obtained at LEP confirms the robustness of our *m*_W_ measurement.

The vector boson (V = W or Z) mass and width are defined in the running-width scheme^35^. The analysis is conducted following the data blinding concept^36^. It is optimized on simulated event samples and a random offset, between −500 MeV and 500 MeV, is applied to the *m*_W_ and *m*_Z_ values until all the procedures are established. In the following sections, we briefly discuss the most important aspects of the *m*_W_ measurement, with further details given in the Methods.

## Event samples and selection criteria

The measurements are made using a sample of pp collisions at $\sqrt{s}=13\,{\rm{TeV}}$ collected in 2016 and corresponding to an integrated luminosity of 16.8 ± 1.2% fb^−1^ (ref. ^37^). The events are preselected by an online trigger algorithm that requires the presence of at least one muon with ${p}_{{\rm{T}}}^{{\rm{\mu }}} > 24\,\mathrm{GeV}$, isolated from other energy deposits in the detector and satisfying quality criteria for tracks reconstructed in the silicon tracker and muon detectors^21,22,24^. Selected W → μν events have exactly one muon with ∣*η*^μ^∣ < 2.4 and $26\,\mathrm{GeV} < {p}_{{\rm{T}}}^{{\rm{\mu }}} < 56\,\mathrm{GeV}$. The selected muon must be isolated from other particles and satisfy selection criteria meant to reduce backgrounds and ensure a high-quality reconstruction. To suppress backgrounds and enhance the purity of the W boson signal, we require ${m}_{{\rm{T}}}^{{\rm{W}}} > 40\,{\rm{GeV}}$; no upper limit on ${m}_{{\rm{T}}}^{{\rm{W}}}$ is imposed. Machine-learning techniques are used to improve the resolution of the reconstructed ${p}_{{\rm{T}}}^{{\rm{miss}}}$ (ref. ^38^), enhancing the separation between signal and background events. The simulated ${p}_{{\rm{T}}}^{{\rm{miss}}}$ distribution is corrected using measured Z → μμ events, as discussed in section ‘Hadronic recoil calibration’. A total of 117 million data events are selected by these criteria.

The selection criteria for the dimuon and W-like *m*_Z_ measurements are designed to be maximally consistent with those of the *m*_W_ analysis. Selected events have exactly two muons satisfying the same criteria, but with ${p}_{{\rm{T}}}^{{\rm{\mu }}} < 60\,\mathrm{GeV}$ because the Z boson is heavier than the W boson. The two muons must have opposite electric charge and a dimuon invariant mass in the 60 GeV < *m*_μμ_ < 120 GeV range. A total of 7.5 million Z → μμ data events are selected. For the W-like *m*_Z_ analysis, only one muon from the Z boson decay is considered to form the $({p}_{{\rm{T}}}^{{\rm{\mu }}},{\eta }^{{\rm{\mu }}},{q}^{{\rm{\mu }}})$ templates. The other muon is treated as a neutrino and excluded from the ${p}_{{\rm{T}}}^{{\rm{miss}}}$ computation^39^. The nominal W-like *m*_Z_ event sample is defined such that only positive muons are considered for selection from odd-numbered events and negative muons are considered for selection from even-numbered events. An alternative sample is defined by reversing the event-number parity and *q*^μ^ matching. Each event is considered only once per sample. The results obtained from the two samples are not fully independent because of the partial overlap of selected events. A total of 7.5 million Z → μμ data events are selected, of which 7.4 million are selected for the nominal W-like *m*_Z_ sample.

Monte Carlo (MC) generators are used to produce large samples of simulated events that are used to guide the analysis and to assess the consistency of the data with different hypotheses for the value of *m*_W_. Simulated W and Z boson event samples are generated with MINNLO_PS_^40,41^, interfaced with PYTHIA^42^. The MINNLO_PS_ predictions are scaled by two-dimensional binned corrections in the W or Z boson *p*_T_ and rapidity obtained with SCETLIB^30,31,43^, thereby achieving N^3^LL + NNLO accuracy and improving the description of the data. The CT18Z PDF set^44^ at NNLO accuracy is used. The detector response is simulated using a detailed description of the CMS detector, implemented with the GEANT4 package^45^. More details on the data sample, event selection and simulation are given in section ‘Event samples and selection criteria’.

The average number of pileup interactions in data is 25, with a tail extending up to 44. The simulated distributions of the number of pileup interactions and the position along the beam line of the pp collision producing the muon are corrected to match the measured distributions, so as to accurately capture their impact on the muon reconstruction efficiency. The efficiency predicted by the simulation is corrected to match that measured in data, as discussed in section ‘Efficiency corrections’.

The main backgrounds in the selected W → μν data sample result from events with nonprompt muons, primarily from decays of heavy-flavour hadrons, or with prompt muons, from Z → μμ decays, in which one muon misses the detector acceptance. Smaller backgrounds include W → τν and Z → ττ events, with muons from τ lepton decays, as well as top quark–antiquark pair, single top quark and diboson production. As discussed in section ‘Nonprompt-muon background determination’, the nonprompt-muon background is evaluated using the extended ABCD method^46^, using sideband regions in data. The uncertainty in the estimated background yields is dominated by the nonprompt-muon component and contributes 3.2 MeV to the uncertainty in *m*_W_.

## High-precision muon momentum calibration

Reconstructing and calibrating the muon momentum requires an exceptionally detailed understanding of the features that affect the trajectories of charged particle tracks. In particular, the alignment of the tracking detector components, the magnetic field throughout the tracking volume and the material distribution, which governs the energy loss and multiple scattering (interactions with electrons or nuclei that lead to small-angle deflections), must be precisely determined. Hits in the silicon tracker are much more important to the track determination than those in the muon system for the ${p}_{{\rm{T}}}^{{\rm{\mu }}}$ range relevant to this analysis. Therefore, we reconstruct the muon momentum exclusively using the silicon pixel and strip detectors, restricting the volume of the detector in which these features must be accurately controlled.

The muon tracks are reconstructed using algorithms and conditions specifically developed for this analysis, including a magnetic field mapping and a material model with a higher precision than those used in the standard CMS reconstruction. The alignment procedure^47^ used to determine the position and orientation of the silicon modules has been extended to include fine-granularity corrections for the magnetic field and energy loss. The correspondence between the measured track curvature and the muon momentum is calibrated using a sample of events in which the dimuon invariant mass is consistent with the well-established mass of the J/Ψ resonance^1^. We extract parameterized corrections in fine bins of *η*^μ^ and extrapolate across the relevant range of ${p}_{{\rm{T}}}^{{\rm{\mu }}}$ using a model that takes into account small offsets in the magnetic field, alignment and tracker material remaining after the initial correction procedure. We validate our results using samples of **Y**(1S) → μμ and Z → μμ events. The uncertainty in the procedure is evaluated from the deviations of the *η*^μ^-binned correction parameters from zero when applying the corrections derived from J*/*Ψ → μμ events to Z → μμ events and constraining *m*_Z_ to the value in ref. ^1^.

Extrapolating corrections to the muon momentum resolution from the relatively low momentum range typical of muons from the J/Ψ meson decay to the higher momentum range of muons from Z boson decays is more challenging than for the momentum scale calibration, because multiple scattering has a large impact on the muon momentum resolution and is highly momentum dependent. For this reason, we correct the muon momentum resolution in simulation to match the measured resolution in data using both J*/*Ψ → μμ and Z → μμ events. The muon momentum calibration contributes 4.8 MeV to the *m*_W_ uncertainty, primarily because of the scale calibration. Further details on the muon momentum scale and resolution calibrations are given in section ‘Muon momentum calibration’.

## Theoretical corrections and uncertainties

The uncertainties in the predictions for Z and W boson production include contributions reflecting the limited knowledge of the PDFs, the missing higher-order (HO) perturbative corrections in the QCD and EW interactions, and the nonperturbative effects. The ${p}_{{\rm{T}}}^{{\rm{W}}}$ spectrum cannot be directly measured with high precision given the limited ${p}_{{\rm{T}}}^{{\rm{miss}}}$ resolution. Although the ${p}_{{\rm{T}}}^{{\rm{Z}}}$ spectrum is measured precisely, using it to infer the ${p}_{{\rm{T}}}^{{\rm{W}}}$ spectrum requires estimating theoretical uncertainties in the ${p}_{{\rm{T}}}^{{\rm{W}}}$/${p}_{{\rm{T}}}^{{\rm{Z}}}$ ratio, which depend strongly on the assumed uncertainty correlation^48^. Therefore, we do not apply corrections derived from the measured ${p}_{{\rm{T}}}^{\mathrm{\mu \mu }}$ spectrum to the W boson simulation. Instead, corrections to the ${p}_{{\rm{T}}}^{{\rm{W}}}$ spectrum come from W → μν data events by the profiling procedure used in the maximum likelihood fits used to extract results. This approach relies on the high accuracy of the theoretical predictions, new techniques to model their uncertainties and correlations across phase space, and the large statistical power of the analysed data sample.

The SCETLIB calculation parameterizes the dominant sources of uncertainty in the W and Z boson transverse momentum spectra (${p}_{{\rm{T}}}^{{\rm{V}}}$) due to perturbative and nonperturbative effects. Perturbative uncertainties are represented by the TNPs of ref. ^32^. The TNPs have a true, but unknown, value that is varied according to its expected magnitude. The closure of this procedure is demonstrated for Z boson production in ref. ^49^, and we have independently verified that fitting a HO prediction (for example, at fourth-order logarithmic accuracy) using a lower order prediction (for example, at N^3^LL with the next-order TNPs) as the fit model yields TNP values consistent with the known values. The calculations treat the quarks as massless. Possible modifications due to the true quark masses are effectively absorbed into the other sources of modelling uncertainty. We have tested other alternatives for the ${p}_{{\rm{T}}}^{{\rm{W}}}$ modelling, at equivalent or higher perturbative orders, and confirmed that the variation in *m*_W_ is within the uncertainty evaluated from our nominal prediction at N^3^LL + NNLO accuracy.

The relative fractions of the Z and W boson helicity states and their uncertainties due to missing HO perturbative corrections are evaluated at NNLO in QCD using MINNLO_PS_; we have verified their consistency with the fixed-order NNLO QCD predictions of DYTURBO^50^ and MCFM^51^. Uncertainties due to the PDFs, including their impact on the W boson helicity states, are evaluated by propagating the Hessian eigenvectors of the CT18Z PDF set^52^. Their contribution to the uncertainty in *m*_W_ is 4.4 MeV. We have repeated the *m*_W_ measurement using seven alternative PDF sets. More details on these studies, corrections and uncertainties are given in the Methods.

## Measurement of the Z and W boson masses

The results are obtained from binned maximum likelihood fits in which systematic uncertainties are represented by nuisance parameters with Gaussian constraints^53^. We allow the systematic uncertainties to be constrained and the central values to be pulled, with respect to their initial values, through the profile likelihood function^1^ used in the fits. Common sources of uncertainty are correlated across bins of the distribution. The parameter of interest, that is, the mass of the W or Z boson (*m*_V_), is an unconstrained parameter in the fit. The effect of different *m*_V_ values on the distributions is derived from a continuous interpolation around the nominal value in the fit, set to the world-average experimental value, and from variations of *m*_V_ by ±100 MeV, evaluated from the full matrix-element-level calculation of the MINNLO_PS_ simulation. We verified that the fit correctly extracts the simulated *m*_W_ value to within 0.1 MeV accuracy for 20 points within this range. The construction and minimization of the likelihood is implemented using the TENSORFLOW software package^54^, in which the use of automatic differentiation^55^ of gradients in the likelihood minimization allows the *m*_W_ and W-like *m*_Z_ likelihood fits to be computationally feasible and numerically stable, despite involving approximately 3,000 bins and 4,000 nuisance parameters.

### Extraction of the Z boson mass from the dimuon mass spectrum

We extract *m*_Z_ from a binned maximum likelihood fit to the dimuon mass distribution, in 25 bins of *m*_μμ_ and 14 bins of *η*^μ^ of the muon with the largest ∣*η*^μ^∣. Compared with the world-average value^1^, dominated by measurements at the LEP collider, we obtain

1 $${m}_{\,{\rm{Z}}}^{\mathrm{\mu \mu }}-{m}_{{\rm{Z}}}^{\mathrm{PDG}}=-2.2\pm 4.8\,\mathrm{MeV}.$$

The largest uncertainties result from the muon momentum calibration (4.6 MeV) and from the size of the data sample (1.0 MeV).

**Fig. 1:**
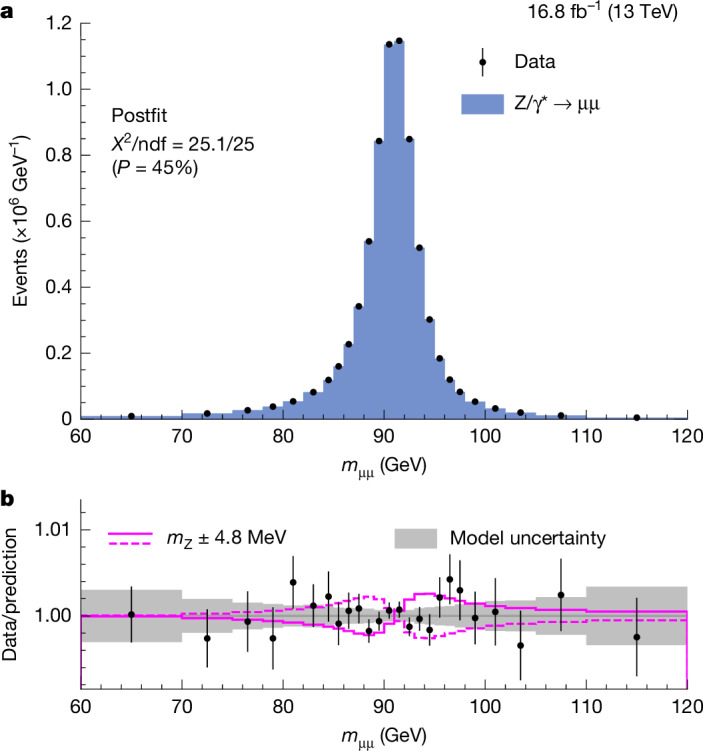
The Z boson mass measurement. **a**, Measured and simulated Z → μμ dimuon mass distributions. The postfit Z → μμ distribution is shown in blue. The small contributions of other processes are included, but not visible. **b**, The ratio of the number of events in data and the total nominal prediction. The vertical bars represent the statistical uncertainties in the data. The total uncertainty in the prediction after the uncertainty profiling procedure (grey band) and the effect of a ±4.8 MeV variation of *m*_Z_ (magenta lines) are also shown, illustrating the precision of the achieved understanding of the distribution.

Figure 1 shows the measured and simulated Z → μμ dimuon mass distributions, with the predictions adjusted to reflect the best-fit values of nuisance parameters obtained from the maximum likelihood fit (referred to as ‘postfit predictions’). The excellent consistency of our result with ${m}_{\,{\rm{Z}}}^{{\rm{PDG}}}$ is a powerful validation of the muon reconstruction, momentum scale calibration and corrections. Although Z → μμ events are not used to determine the values of the parameterized muon momentum scale calibration, they are used, together with the ${m}_{\,{\rm{Z}}}^{{\rm{PDG}}}$ value^1^, to define the systematic uncertainties, as described in section ‘Muon momentum calibration’. Therefore, our *m*_Z_ value is not a measurement independent of the experimental world average.

### W-like measurement of the Z boson mass

The W-like *m*_Z_ analysis extracts *m*_Z_ from a binned maximum likelihood fit to the $({p}_{{\rm{T}}}^{{\rm{\mu }}},{\eta }^{{\rm{\mu }}},{q}^{{\rm{\mu }}})$ distribution of the selected muons. As described in section ‘Event samples and selection criteria’, two event samples are used. The result for the analysis configuration selecting positive muons in odd event-number events, compared with the experimental *m*_Z_ average^1^, is

$${m}_{\,{\rm{Z}}}^{{\rm{W}}{\rm{-like}}}-{m}_{{\rm{Z}}}^{{\rm{PDG}}}=-6\pm 7{\rm{(stat)}}\pm 12{\rm{(syst)}}=-6\pm 14\,{\rm{MeV}},$$

showing that ${m}_{\,{\rm{Z}}}^{{\rm{W}}{\rm{-like}}}$ agrees with ${m}_{\,{\rm{Z}}}^{{\rm{PDG}}}$ (and with ${m}_{\,{\rm{Z}}}^{\mathrm{\mu \mu }}$). Both the helicity fit analysis and the analysis using the configuration with the alternative muon charge and event-number parity matching provide *m*_Z_ values that agree with the baseline result to within one standard deviation.

We validate the accuracy of the theory modelling and corresponding uncertainties by measuring the ${p}_{{\rm{T}}}^{{\rm{Z}}}$ directly in Z → μμ events. Using the Z boson production model described in the previous section, we fit the two-dimensional distribution of the dimuon *p*_T_ and rapidity $({p}_{{\rm{T}}}^{{\rm{\mu \mu }}},{y}^{\mathrm{\mu \mu }})$ to the observed Z → μμ data. The consistency of the predictions and their uncertainties with the data is assessed with a goodness-of-fit test based on a saturated model, in which an unconstrained normalization parameter is introduced for each bin of the likelihood^56^. We conclude that the model and the data are compatible, given the *P*-value of 16% that is evaluated from the ratio of the nominal and saturated likelihoods.

**Fig. 2:**
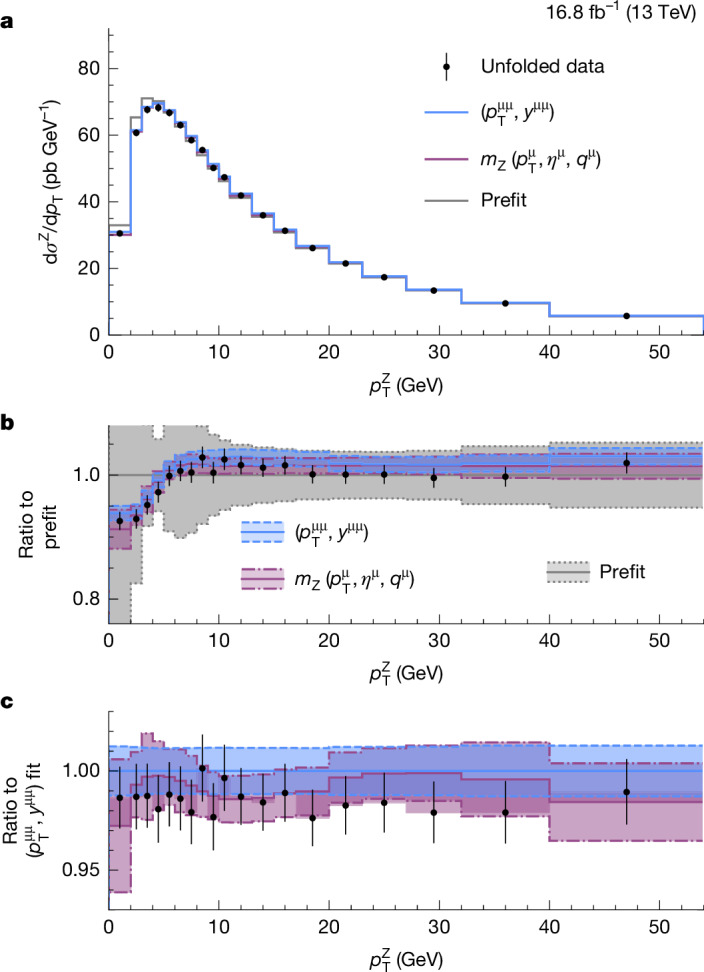
Validation of the theory model. **a**, Unfolded measured ${p}_{{\rm{T}}}^{{\rm{Z}}}$ distribution (points) compared with the generator-level SCETLIB + MINNLO_PS_ predictions before (prefit, grey) and after adjusting the nuisance parameters to the best-fit values obtained from the W-like *m*_Z_ fit (magenta) or from the direct fit to the ${p}_{{\rm{T}}}^{\mathrm{\mu \mu }}$ distribution (blue). **b**, The ratio of the predictions and unfolded data to the prefit prediction. The uncertainty in the prefit prediction is shown by the shaded grey area. **c**, The ratio of the predictions and unfolded data to the postfit prediction from the fit to the $({p}_{{\rm{T}}}^{\mathrm{\mu \mu }},{y}^{\mathrm{\mu \mu }})$ distribution. The postfit uncertainties in the predictions are shown in the shaded magenta and blue bands. The vertical bars represent the total uncertainty in the unfolded data.

The results of the ${p}_{{\rm{T}}}^{\mathrm{\mu \mu }}$ fit are not an input to the W-like *m*_Z_ or *m*_W_ measurements. Rather, we independently determine values for the nuisance parameters describing the ${p}_{{\rm{T}}}^{{\rm{Z}}}$ modelling from the W-like *m*_Z_ measurement and verify that they are consistent with those from the direct fit to ${p}_{{\rm{T}}}^{\mathrm{\mu \mu }}$. Figure 2 shows the generator-level ${p}_{{\rm{T}}}^{{\rm{Z}}}$ distribution, with the predictions adjusted by the nuisance parameter values obtained from the two independent fits to the data. To test the accuracy of the adjusted predictions in describing our data, we account for effects of the detector response and resolution by ‘unfolding’ our measurement to the generator level, as described in section ‘Additional validation of theoretical modelling’. The self-consistency of the postfit distributions from the two fits, as well as their consistency with the data, confirms the robustness of the predictions and of the uncertainty model, and demonstrates the ability of the $({p}_{{\rm{T}}}^{{\rm{\mu }}},{\eta }^{{\rm{\mu }}})$ distribution to constrain the ${p}_{{\rm{T}}}^{{\rm{V}}}$ modelling in situ. This result supports our treatment of the W boson production modelling in the *m*_W_ analysis.

More details on the stability of our W-like Z boson mass measurement under different modelling assumptions and its consistency with the measured ${p}_{{\rm{T}}}^{\mathrm{\mu \mu }}$ distribution are provided in section ‘Additional validation of theoretical modelling’.

### Measurement of the W boson mass

Having validated the analysis steps using the Z boson data, we proceed with the determination of the W boson mass. A fit is performed to the $({p}_{{\rm{T}}}^{{\rm{\mu }}},{\eta }^{{\rm{\mu }}},{q}^{{\rm{\mu }}})$ distribution, shown in Extended Data Fig. 9, and the observed *m*_W_ value is

$${m}_{{\rm{W}}}=80,360.2\pm 2.4\mathrm{(stat)}\pm 9.6\mathrm{(syst)}=\mathrm{80,360.2}\pm 9.9\,\mathrm{MeV},$$

in agreement with the EW fit prediction, *m*_W_ = 80,353 ± 6 MeV (refs. ^1–3^), and with other experimental results, except the latest measurement reported by the CDF Collaboration^4^. The EW fit prediction is based on relationships between *m*_W_ and other experimental observables, including the Z boson, Higgs boson and top quark masses, the fine-structure constant and the muon lifetime. The uncertainty in the prediction is due to missing HO terms in the perturbative calculation used to derive the predicted relationship between the experimental inputs and from uncertainties in the experimental inputs themselves. The two sources of uncertainty are of comparable size.

**Fig. 3:**
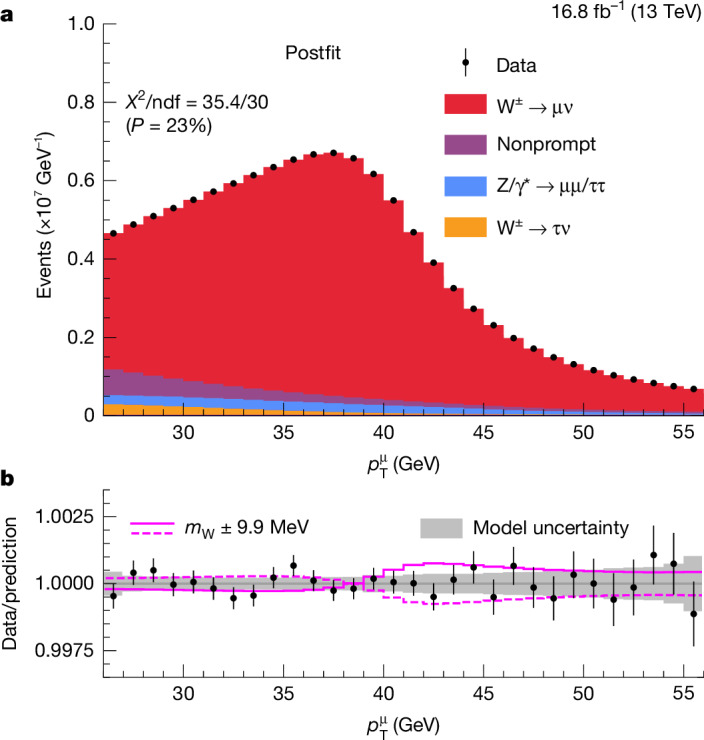
The W boson mass measurement. **a**, Measured and postfit ${p}_{{\rm{T}}}^{{\rm{\mu }}}$ distributions, showing the sensitivity to *m*_W_ from the characteristic peak at about *m*_W_/2. The predicted W → μν contribution, shown in red, reflects the measured value of *m*_W_. The dominant background contributions are shown as coloured filled histograms. **b**, The ratio between the number of events observed in data, including variations in the predictions, and the total nominal prediction. The vertical bars represent the statistical uncertainties in the data. A shift in the *m*_W_ value shifts the peak of the distribution, as shown by the solid and dashed magenta lines, which show an increase or decrease in *m*_W_ by 9.9 MeV. The total contribution of all theoretical and experimental uncertainties in the predictions, after the uncertainty profiling in the maximum likelihood fit, is shown by the grey band.

Figure 3 compares the measured and the postfit predicted ${p}_{{\rm{T}}}^{{\rm{\mu }}}$ distributions, with *m*_W_ adjusted to the observed value. The effect on the ${p}_{{\rm{T}}}^{{\rm{\mu }}}$ distribution of a 9.9 MeV variation in *m*_W_ is shown to illustrate the degree to which the distribution and its uncertainties are controlled, enabling the high precision of the measurement. The main uncertainties in the *m*_W_ measurement are due to the muon momentum calibration (4.8 MeV) and the PDF uncertainties (4.4 MeV). Detailed breakdowns of the *m*_W_ measurement uncertainty are provided in Extended Data Table 2. The robustness of the result with respect to the theory model is tested further by performing the *m*_W_ measurement with the helicity fit, as discussed in section ‘Helicity fit’. The result, 80,360.8 ± 15.2 MeV, is consistent with the nominal value.

## Discussion

In this paper, we report the first W boson mass measurement by the CMS Collaboration at the CERN LHC. The result is markedly more precise than previous LHC measurements. The W boson mass is extracted from a sample of 117 million selected W → μν events, collected in 2016 at the proton–proton collision energy of 13 TeV, using a highly granular binned maximum likelihood fit to the three-dimensional distribution of the muon ${p}_{{\rm{T}}}^{{\rm{\mu }}}$, *η*^μ^ and electric charge. New experimental techniques have been used, together with state-of-the-art theoretical models, to improve the measurement accuracy. The muon momentum calibration, based on J*/*Ψ → μμ decays, as well as the data analysis methods and the treatment of the theory calculations used in the *m*_W_ measurement have been extensively validated by extracting *m*_Z_ and ${p}_{{\rm{T}}}^{{\rm{Z}}}$ both from a direct Z → μμ dimuon analysis and from a W-like analysis of the Z boson data.

**Fig. 4:**
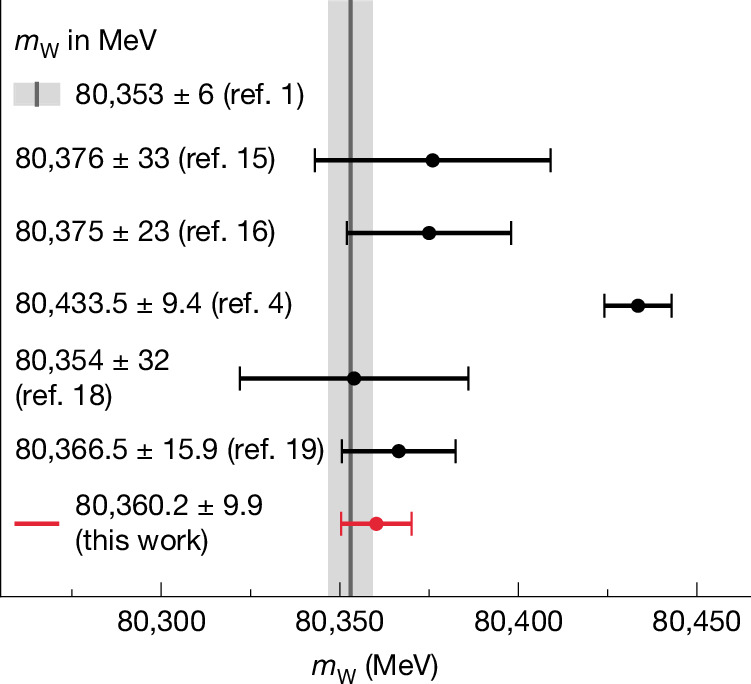
Comparison with other experiments and the EW fit prediction. The *m*_W_ measurement from this analysis (in red) is compared with the combined measurement of experiments at LEP^15^, and with the measurements performed by the D0 (ref. ^16^), CDF (ref. ^4^), LHCb (ref. ^18^) and ATLAS (ref. ^19^) experiments. The global EW fit prediction^1–3^ is represented by the grey vertical band, with the shaded band showing its uncertainty.

As shown in Fig. 4, the measured value, *m*_W_ = 80,360.2 ± 9.9 MeV, agrees with the standard model expectation from the electroweak fit and is in disagreement with the measurement reported by the CDF Collaboration. Our result has similar precision to the CDF Collaboration measurement and is significantly more precise than all other measurements. The dominant sources of uncertainty are the muon momentum calibration and the PDFs. Uncertainties in the modelling of W boson production are subdominant because of new approaches used to parameterize and constrain the predictions and their corresponding uncertainties in situ with the data. This result constitutes a marked step towards achieving an experimental measurement of *m*_W_ with a precision matching that of the EW fit.

## Methods

### Event samples and selection criteria

The dataset used for this analysis, roughly half of the full 2016 sample, ensures an optimal performance of the CMS detector, especially for the reconstruction of charged particle tracks^47^. The data and simulation were processed with the most recent version of the reconstruction software, including improvements to particle identification and reconstruction developed for this analysis, and with the latest detector calibration and description of the operating conditions.

We simulate W and Z boson production at NNLO in QCD using the MINNLO_PS_ Wj and Zj^40,41^ (rev. 3900) processes in POWHEG-BOX-V2 (refs. ^57–59^), interfaced with PYTHIA 8.240 (ref. ^42^) for the parton shower and hadronization, and with PHOTOS++3.61 (refs. ^60,61^) for final-state photon radiation. The Z boson event samples simulate all contributions to the dilepton final state, including those from virtual photons. We use the CP5 underlying event tune^62^, with the hard primordial-*k*_T_ parameter set to 2.225 GeV, obtained from a dedicated optimization using the ${p}_{{\rm{T}}}^{{\rm{\mu \mu }}}$ data in ref. ^63^. The (*G*_μ_, *m*_W_, *m*_Z_) and $({G}_{{\rm{\mu }}},{\sin }^{2}{\theta }_{\mathrm{eff}},{m}_{{\rm{Z}}})$ EW input schemes are used for W and Z boson production, respectively. The CT18Z PDF set^44^ at NNLO accuracy was chosen for the nominal analysis, before unblinding the result, given its good description of our W and Z data and because the expected shifts in *m*_W_ from using other modern PDF sets are within its uncertainties. Additional NNLO PDF sets are studied using event-level weights in the POWHEG MINNLO_PS_ sample: NNPDF3.1 (ref. ^64^), NNPDF4.0 (ref. ^65^), CT18 (ref. ^44^), MSHT20 (ref. ^66^) and PDF4LHC21 (ref. ^67^). We also consider the MSHT20aN3LO approximate N^3^LO PDF set^68^. The POWHEG MINNLO_PS_ generator is also used to simulate events with W or Z bosons decaying to τ leptons, with the same theory corrections on the boson production kinematic distributions as those applied to the samples with muonic decays. To ensure that the MC sample size is not a notable source of uncertainty in the measurement, simulated samples of more than 4 billion W boson production events and 400 million Z boson production events have been produced. The EW production of lepton pairs or of a W boson in association with a quark through photon–photon or photon–quark scattering is simulated at LO using PYTHIA 8.240 (ref. ^69^). Top quark and diboson production are simulated at NLO QCD accuracy using MADGRAPH 5_aMC@NLO v.2.6.5 (ref. ^70^) and POWHEG-BOX-V2 (ref. ^71^), respectively, interfaced with PYTHIA 8.240 for the parton shower and hadronization. Quarkonia production is simulated using PYTHIA 8 interfaced with PHOTOS++ v.3.61 for final-state photon radiation. Single-muon events have been simulated for additional validation of the muon reconstruction and calibration.

Although the muon system is not used for the muon momentum evaluation, it is crucial for triggering and identification. The selected muons must have a reconstructed track in both the silicon tracker and the muon detectors, with a consistent track fit for hits in both detector subsystems, and pass additional quality criteria to ensure a high purity of the selected events. We use the ‘medium’ identification working point^25^, whose efficiency is better than 98% for signal muons. The muons must have a transverse impact parameter smaller than 500 μm with respect to the beam line and be isolated from hadronic activity in the detector. The muon isolation is defined as the pileup-corrected ratio between ${p}_{{\rm{T}}}^{{\rm{\mu }}}$ and the sum of the *p*_T_ of all other reconstructed physics objects within a cone centred around the muon^26^. The isolation of selected muons must be smaller than 15%, using a cone of radius $\Delta R=\sqrt{{(\Delta \phi )}^{2}+{(\Delta \eta )}^{2}}=0.4$, where Δ*ϕ* and Δ*η* are, respectively, the distance in the *ϕ* and *η* coordinates between the muon and the physics objects considered in the sum. Only charged particles within 2 mm of the muon track along the beam axis are considered in the isolation sum. The distance is evaluated between the points of closest approach to the beam line for each track. The same criteria are used to select charged particles used in the ${p}_{{\rm{T}}}^{{\rm{miss}}}$ calculation. Our definition differs from the standard CMS approach, where charged particles in the isolation and ${p}_{{\rm{T}}}^{{\rm{miss}}}$ sums are defined with respect to the vertex that maximizes the sum of ${p}_{{\rm{T}}}^{{\rm{2}}}$ of the associated physics objects^72^. This change of definition is needed to minimize the rate at which the wrong vertex is chosen, which is negligible in Z → μμ events but, with the standard CMS algorithm, ranges from 1% to 5% for W → μν events, depending on ${p}_{{\rm{T}}}^{{\rm{W}}}$. To ensure the validity of the isolation and ${p}_{{\rm{T}}}^{{\rm{miss}}}$ corrections measured with Z → μμ events and applied to W → μν events (as described in sections ‘Efficiency corrections’ and ‘Hadronic recoil calibration’), it is important to make sure that there are no differences in their dependence on the vertex selection.

Muons used in both the *m*_Z_ and *m*_W_ analyses are selected by the same trigger, requiring the presence of at least one muon with ${p}_{{\rm{T}}}^{{\rm{\mu }}} > 24\,\mathrm{GeV}$, to guarantee maximal consistency in terms of event selection and efficiency corrections. Events with electrons with *p*_T_ > 10 GeV (or additional muons with *p*_T_ > 15 GeV) or satisfying looser identification criteria are rejected^25,26^. In the *m*_W_ analysis, the selected muon must have $26 < {p}_{{\rm{T}}}^{{\rm{\mu }}} < 56\,\mathrm{GeV}$. The upper threshold is increased to 60 GeV for the W-like *m*_Z_ measurement. These thresholds restrict the selected events to the ${p}_{{\rm{T}}}^{{\rm{\mu }}}$ range, in which the trigger and reconstruction efficiencies are measured most accurately. The selected muon must be geometrically matched to the object that triggered the event, within a cone of radius Δ*R* = 0.3. In the W-like analysis, in which two muons are reconstructed, the matching is required only for the muon used to form the $({p}_{{\rm{T}}}^{{\rm{\mu }}},{\eta }^{{\rm{\mu }}})$ template. This choice avoids the need to evaluate correlations in the triggering efficiency in events in which both muons satisfy the trigger requirements. For consistency with the W boson selection, W-like events must satisfy *m*_T_ > 45 GeV (about *m*_V_/2). In this case, *m*_T_ is calculated from the selected muon and the ${{\bf{p}}}_{{\rm{T}}}^{{\rm{miss}}}$ value obtained by excluding the other muon from the vector sum.

Events are rejected if they contain electrons with *p*_T_ > 10 GeV satisfying the identification criteria of the veto working point (which has 95% efficiency for genuine electrons^26^) or additional muons of *p*_T_ > 15 GeV matching the loose criteria (with an efficiency of above 99% for real muons^25^). The electron veto rejects the residual contribution of events from top quark and boson pair production, and from Z →ττ decays with one τ lepton decaying to a muon and the other to an electron. The electron veto efficiency has a negligible impact on the analysis. The muon veto efficiency and the corresponding uncertainties are discussed in the next section.

The single-muon selection efficiency is 85%, evaluated from simulated W → μν and Z → μμ events. The fraction of W → μν events in the selected data sample is 89%. The signal purity of the selected dimuon sample is larger than 99.5%, given the stronger suppression of the backgrounds due to the double muon selection and invariant mass requirement. Although the W-like *m*_Z_ analysis provides a stringent test of the analysis strategy in an almost background-free environment, the significant background from nonprompt muons in the *m*_W_ analysis must be validated by other means, as discussed in section ‘Nonprompt-muon background determination’.

### Efficiency corrections

The *m*_W_ measurement is based on a fit to the measured $({p}_{{\rm{T}}}^{{\rm{\mu }}},{\eta }^{{\rm{\mu }}},{q}^{{\rm{\mu }}})$ distribution using simulated templates for the signal and most background processes. Therefore, it is important that the simulation can accurately reproduce the efficiency of the event selection in the $({p}_{{\rm{T}}}^{{\rm{\mu }}},{\eta }^{{\rm{\mu }}})$ bins used in the analysis. Corrections to the simulated muon efficiencies are determined from data with the tag-and-probe (T&P) method^73^, using events from the same Z → μμ sample that we use in the analysis, except that we apply a looser event selection.

The efficiencies are measured differentially in $({p}_{{\rm{T}}}^{{\rm{\mu }}},{\eta }^{{\rm{\mu }}})$ for different stages of the muon selection, factorized as: reconstruction of a standalone track in the muon chambers; matching of a standalone muon with a track in the tracker to form a global muon candidate (tracking); impact parameter and identification quality criteria of the global muon track; trigger selection; and muon isolation. The efficiencies are evaluated in the measured and simulated event samples, for each of the five sequential stages, and their ratios are used as scale factors (SFs) to reweight the simulated events. Efficiencies are determined from the fraction of selected events in which the probe muon passes the selection whose efficiency is being evaluated. Background events with at least one nonprompt muon are subtracted when computing the efficiency in data. These background contributions are estimated by fitting the sum of a signal and a background model to the observed *m*_μμ_ distribution. The Z → μμ contribution is modelled by a simulated template from the MINNLO_PS_ sample, convolved with a Gaussian shape to account for differences in the momentum scale and resolution between data and simulation. An alternative signal model, defined by the convolution of a Breit–Wigner distribution and a resolution function that has a Gaussian core and asymmetric exponential tails, is used to assess the systematic uncertainty. The background component is modelled using an exponential function, except for the reconstruction and tracking steps in the failing probe samples, for which the background fraction is large and its shape at low *m*_μμ_ is sculpted by the ${p}_{{\rm{T}}}^{{\rm{\mu }}}$ selection. For these steps, the background model is an exponential decay distribution that transitions to an error function for *m*_μμ_ < *m*_Z_ to capture threshold effects. Third- or fourth-order polynomials are tested as alternative background shapes.

Misalignment or other effects in the reconstruction of tracks in the muon chambers, which are used for triggering and identification purposes, can result in charge-dependent biases in the measured efficiencies. To properly account for them, efficiencies are measured separately for each muon charge except for the isolation step, for which the charge asymmetry is found to be negligible. The largest asymmetry is in the trigger SFs, rising to 5% in the most forward region of the detector and for ${p}_{{\rm{T}}}^{{\rm{\mu }}} < 35\,\mathrm{GeV}$. The muon isolation is sensitive to the vector sum of the momenta of charged and neutral hadrons in the event, referred to as the hadronic recoil (**u**_T_). The angular distance between the muon and **u**_T_ is different between W → μν and Z → μμ events, leading to a bias in the muon isolation efficiency measured using Z boson decays. For a given ${p}_{{\rm{T}}}^{{\rm{Z}}}$ value, the bias is larger for low ${p}_{{\rm{T}}}^{{\rm{\mu }}}$, when the muon is more likely produced in the direction opposite to that of the Z boson *p*_T_ and in the vicinity of the recoil. The trigger efficiencies are also affected because of the isolation requirement applied at the trigger level. To account for this effect, the trigger and isolation efficiencies are measured triple-differentially in the muon $({p}_{{\rm{T}}}^{{\rm{\mu }}},{\eta }^{{\rm{\mu }}})$ and in the projection of **u**_T_ along the ${p}_{{\rm{T}}}^{{\rm{\mu }}}$ direction, ${u}_{{\rm{T}}}^{{\rm{\mu }}}$. The corrections are applied to W → μν events using the W boson recoil, after correcting its distribution as described in the next section.

The statistical uncertainty in the SFs originates from the limited sample of measured and simulated Z → μμ events in the T&P estimate, whereas systematic uncertainties stem from the modelling of the Z → μμ mass distributions with signal and background components when extracting the efficiencies in the measured event sample. We evaluate these systematic uncertainties by repeating the efficiency measurements in the data sample after varying the signal or background models, taking the difference with respect to the nominal efficiency as the uncertainty.

To mitigate the effects of statistical fluctuations and discrete bin edges, the SFs are smoothed as a function of ${p}_{{\rm{T}}}^{{\rm{\mu }}}$ using a polynomial interpolation. Third-order polynomials (second-order for the tracking step) properly model the ${p}_{{\rm{T}}}^{{\rm{\mu }}}$ dependence of the binned SFs, as determined with statistical tests. Trigger and isolation SFs are smoothed using two-dimensional polynomials in the $({u}_{{\rm{T}}}^{{\rm{\mu }}},{p}_{{\rm{T}}}^{{\rm{\mu }}})$ space, with third order in the ${p}_{{\rm{T}}}^{{\rm{\mu }}}$ direction and second order in the ${u}_{{\rm{T}}}^{{\rm{\mu }}}$ direction. The smoothing acts independently on each *η*^μ^ bin, and no smoothing is performed compared with *η*^μ^ because physical boundaries in the detector might produce genuine discontinuities in the *η*^μ^ dependence of the efficiency. Instead, a smooth dependence on ${p}_{{\rm{T}}}^{{\rm{\mu }}}$ is expected in the momentum range of interest. The smoothing simplifies the treatment of the SF statistical uncertainties in the analysis fit and also leads to reduced uncertainties in *m*_W_ by imposing that the measured SFs are correlated across the ${p}_{{\rm{T}}}^{{\rm{\mu }}}$ or $({u}_{{\rm{T}}}^{{\rm{\mu }}},{p}_{{\rm{T}}}^{{\rm{\mu }}})$ bins. We have verified with pseudo-data tests that, within the SF uncertainties, neither the smoothing procedure nor the chosen polynomial order induces a bias in the measured value of *m*_W_.

Statistical uncertainties in the SFs are implemented in the likelihood as 2,784 nuisance parameters, defined from the independent variations of the smoothing fit parameters according to the eigenvectors of their covariance matrix for each of the 48 *η*^μ^ bins. The number of ${p}_{{\rm{T}}}^{{\rm{\mu }}}$ variations is determined by the order of the smoothing polynomial used for each step. These nuisance parameters are uncorrelated versus *η*^μ^ and *q*^μ^, and modify the ${p}_{{\rm{T}}}^{{\rm{\mu }}}$ spectrum in a continuous way.

Systematic uncertainties in the SFs are estimated as the difference, after the ${p}_{{\rm{T}}}^{{\rm{\mu }}}$ smoothing, between the nominal and alternative SFs resulting from the variation of the signal or background models in the T&P mass fits. These are correlated across the $({p}_{{\rm{T}}}^{{\rm{\mu }}},{\eta }^{{\rm{\mu }}},{q}^{{\rm{\mu }}})$ bins, because the same signal and background models are used in all T&P fits. However, we also implement additional uncertainties uncorrelated among *η*^μ^ bins, resulting in 49 nuisance parameters assigned to each efficiency step to account for the change in the T&P signal model. Similar uncertainties are implemented for the reconstruction and tracking SFs to reflect the change in the background model. In total, the systematic uncertainty in the SFs is encoded in 343 nuisance parameters, correlated between muon charges. The statistical and systematic components of the SF uncertainties, after the smoothing, have a similar contribution to the uncertainty in *m*_W_, and their combined effect is 3.0 MeV.

Dedicated SFs and uncertainties are derived for the muon veto selection used in the single-muon analysis. These SFs are used to correct the simulated yields of the Z boson background process in events where the second prompt muon falls inside the $({p}_{{\rm{T}}}^{{\rm{\mu }}},{\eta }^{{\rm{\mu }}})$ acceptance window but fails the reconstruction or identification criteria of the veto. This component of the Z → μμ background is characterized by a ${p}_{{\rm{T}}}^{{\rm{\mu }}}$ distribution for the selected muon similar to that of W boson decays, but with the peak located at higher values of ${p}_{{\rm{T}}}^{{\rm{\mu }}}$. Moreover, because of the high efficiency of the veto selection, close to unity in many $({p}_{{\rm{T}}}^{{\rm{\mu }}},{\eta }^{{\rm{\mu }}})$ bins, small efficiency variations between data and simulation can result in relatively large corrections for the probability to fail the veto. Therefore, although this background component constitutes a small fraction of the total Z → μμ background, its shape and normalization must be accurately controlled to avoid a bias in the measured *m*_W_. The veto SFs are determined and smoothed with the same technique as for other SFs, but are applied to simulated events as a function of the $({p}_{{\rm{T}}}^{{\rm{\mu }}},{\eta }^{{\rm{\mu }}})$ of the second generator-level muon (evaluated after final-state radiation), taken as a proxy for the nonreconstructed muon.

The veto SFs are measured for ${p}_{{\rm{T}}}^{{\rm{\mu }}} > 15\,\mathrm{GeV}$, split by *q*^μ^ and factorized as three independent terms accounting for muon reconstruction, tracking and loose identification. They differ from the nominal analysis SFs because of a slight tuning of the matching criteria between the inner and outer muon track to cope with the lower ${p}_{{\rm{T}}}^{{\rm{\mu }}}$ threshold. Uncertainties in veto SFs are encoded in 581 nuisance parameters, affecting only the Z → μμ background. The statistical uncertainty derives from varying the parameters of the ${p}_{{\rm{T}}}^{{\rm{\mu }}}$ smoothing polynomials, independently in each *η*^μ^ bin and *q*^μ^. Systematic uncertainties, related to the *m*_μμ_ modelling in the T&P fits, are implemented following the same *η*^μ^ granularity and correlation scheme as the standard SFs.

The contribution of the veto SFs to the uncertainty in *m*_W_ is smaller than 0.5 MeV, reflecting the fact that the Z boson background sensitive to these SFs is strongly suppressed by the veto. The nominal muon veto restricts the selection to ‘global muons’, which have a high-quality track in both the tracker and muon detectors. An alternative definition has also been tested, with the muon inner track not required to be matched to a track reconstructed in the muon detectors. This looser selection has higher efficiency for prompt muons and, therefore, provides better rejection of the Z → μμ background, at the cost of larger systematic uncertainties in the measured veto SFs because of the combination of different categories of reconstructed muons. Tests using pseudo-data generated with either veto selection have been carried out, showing that the measured *m*_W_ values agree within less than 0.1 MeV between the two veto selections and that the residual bias in *m*_W_ is covered by the veto SF uncertainties.

Further corrections and corresponding uncertainties are applied to the simulated events. Partial mistiming of signals in the muon detectors led to the incorrect assignment of the triggered event to the previous proton bunch crossing for a small fraction of events^22^. This is known as ‘prefiring’, and caused a reduction in the trigger efficiency. A correction for this effect is determined in bins of *η*^μ^ and ${p}_{{\rm{T}}}^{{\rm{\mu }}}$ (ref. ^74^). The correction increases with *η*^μ^ and varies between 0.5% and 2%. A similar issue originating from the prefiring of the electromagnetic calorimeter triggers affects the analysis through hadronic jets containing photons or electrons not rejected by the veto. The total contribution of the prefiring to the uncertainty in *m*_W_ is about 0.7 MeV.

**Extended Data Fig. 1:**
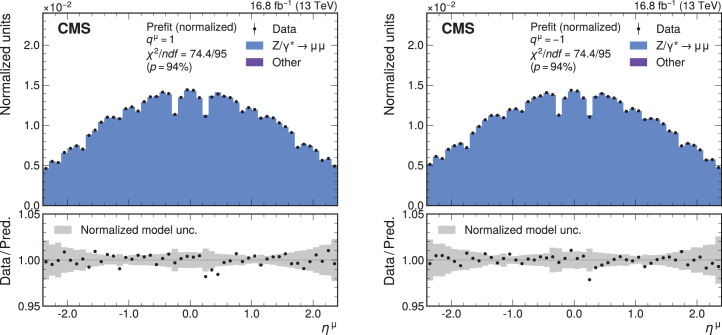
Measured and predicted *η*^μ^ distributions in Z → μμ events with the W-like Z boson selection for positively (left) and negatively (right) charged muons. The normalization of the simulated spectrum is scaled to the measured distribution to better illustrate the level of agreement between the two. The shaded bands correspond to the total (statistical and systematic) uncertainty on the normalized distributions. The normalization of the prediction is scaled by about 1%, which is less than the luminosity and total cross section uncertainties. The vertical bars represent the statistical uncertainties in the data. The bottom panel shows the ratio of the number of events observed in data and of normalized variations in the predictions to that of the total nominal prediction.

The quality of the experimental corrections applied to the simulation is validated using Z → μμ events, which offer a pure sample of prompt muons, comparing the predicted distribution of the selected muon *η*^μ^ with the measured one, for each muon charge, as shown in Extended Data Fig. 1. The agreement between measured and simulated data is within 2% in all bins, and the difference is covered by the uncertainty.

### Hadronic recoil calibration

To further improve the modelling of ${p}_{{\rm{T}}}^{{\rm{miss}}}$ and *m*_T_ in the simulation, hadronic recoil corrections are derived using measured Z → μμ events, by exploiting the relation between **u**_T_ and the ${{\bf{p}}}_{{\rm{T}}}^{\mathrm{\mu \mu }}$ vector, ${{\bf{u}}}_{{\rm{T}}}=-{{\bf{p}}}_{{\rm{T}}}^{\mathrm{\mu \mu }}$. The components of **u**_T_ parallel and perpendicular to ${{\bf{p}}}_{{\rm{T}}}^{\mathrm{\mu \mu }}$ are modelled independently with spline-based parameterizations as functions of ${p}_{{\rm{T}}}^{{\rm{\mu \mu }}}$, for both data and simulated events. The parameterization yields two-dimensional PDFs, expressed in terms of ${p}_{{\rm{T}}}^{{\rm{\mu \mu }}}$ and the **u**_T_ component. Although the resulting parameters are not directly physical, they provide a smooth and flexible description of the **u**_T_ magnitude (*u*_T_) over the full ${p}_{{\rm{T}}}^{{\rm{\mu \mu }}}$ range.

**Extended Data Fig. 2:**
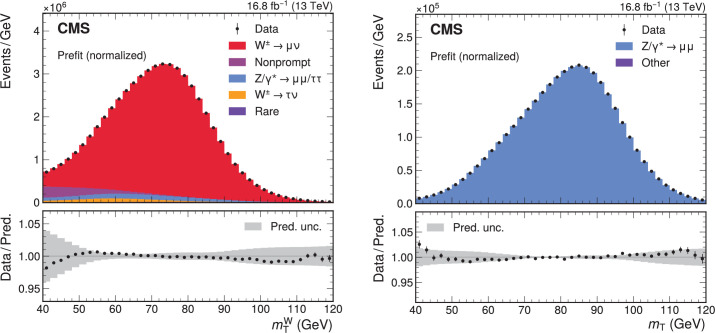
Measured and predicted *m*_T_ distributions in Z → μμ and W → μν events, after calibrating *u*_T_. Given that ${m}_{{\rm{T}}}^{{\rm{W}}}$ is an input variable, the extended ABCD method does not give suitable predictions for the ${m}_{{\rm{T}}}^{{\rm{W}}}$ distribution. Therefore, the nonprompt background contribution to W boson production is estimated from the QCD multijet simulation. The predictions are those prior to the fit to data. The total uncertainties (statistical and systematic) are represented by the gray band and the normalization of the simulated spectrum is scaled to the measured distribution to better illustrate their agreement. The vertical bars represent the statistical uncertainties in the data. The bottom panel shows the ratio of the number of events observed in data and of variations in the predictions to that of the total nominal prediction.

Subsequently, for each simulated event, a new value of ${p}_{{\rm{T}}}^{{\rm{miss}}}$ is computed using an inverse cumulative distribution function transformation of the function mapping the simulated templates to data. The corrections, derived from Z → μμ events and parameterized in ${p}_{{\rm{T}}}^{{\rm{\mu \mu }}}$, are applied to simulated W → μν events as a function of the ${p}_{{\rm{T}}}^{{\rm{W}}}$, where the ${p}_{{\rm{T}}}^{{\rm{W}}}$ is built from the reconstructed muon and the generator-level neutrino. Extended Data Fig. 2 shows the *u*_T_-corrected transverse mass distribution for Z → μμ and W → μν events. Supplementary Fig. 18 shows the impact of the *u*_T_ correction on the parallel and perpendicular components of **u**_T_ for Z → μμ events. Apart from a slight disagreement in normalization between the measured and simulated distributions (unrelated to *u*_T_ and accounted for by other uncertainties), the scale and resolution of the corrected *u*_T_ in simulation match those of the data at the sub-per cent level.

The uncertainty in the corrections is evaluated from the statistical uncertainty of the fits that parameterize the correction of the simulation to the data. Their impact on *m*_W_ is assessed by varying the correction parameters according to the eigenvectors of the fit covariance matrix. We have verified that their contribution to the uncertainty in *m*_W_ is below 0.3 MeV. Because these variations are computationally expensive to evaluate, and their contribution to the *m*_W_ uncertainty is negligible, they are not included in the nominal fit configuration.

### Nonprompt-muon background determination

The nonprompt-muon background consists primarily of events in which muons originate from decays of heavy-flavour hadrons. Despite the large suppression applied by the muon selection criteria, a significant contribution from this background remains in the W boson selection. We evaluate it using data from sideband regions defined by inverting the *m*_T_ selection, the muon isolation requirement, or both. To account for correlations between the isolation and the *m*_T_ sideband regions, the ‘extended ABCD method’ proposed in ref. ^46^ is used. In this method, the low-*m*_T_ sideband region is divided into two regions with *m*_T_ < 20 GeV or 20 < *m*_T_ < 40 GeV, each one further split into events passing or failing the muon isolation criterion, such that the signal region is complemented by five sideband regions of isolation and *m*_T_, compared with the typical three of the classic ABCD method. The extended ABCD method accounts for a linearly varying isolation efficiency as a function of *m*_T_, exploiting the two low-*m*_T_ regions to extrapolate the expected isolation efficiency to the third high-*m*_T_ region, contrary to the standard ABCD approach in which a constant efficiency is assumed across the entire *m*_T_ space.

In each sideband region, the nonprompt-muon component is evaluated by subtracting from the data the contribution of processes with prompt muons, estimated from simulation. For each bin of $({p}_{{\rm{T}}}^{{\rm{\mu }}},{\eta }^{{\rm{\mu }}},{q}^{{\rm{\mu }}})$, the two low-*m*_T_ regions are used to obtain a transfer factor that is applied to events that satisfy the *m*_T_ selection of the signal region but fail the muon isolation requirement, to obtain an estimate of the nonprompt-muon background in the signal region.

To reduce the impact on *m*_W_ of the statistical fluctuations in the nonprompt-muon background template, the ${p}_{{\rm{T}}}^{{\rm{\mu }}}$ distribution from the ABCD prediction is smoothed using an exponential of a third-order polynomial. The minimum order of the polynomial to correctly describe the ${p}_{{\rm{T}}}^{{\rm{\mu }}}$ shape is determined based on statistical tests. The statistical uncertainties of the data are accounted for by propagating the uncertainties in the smoothing function parameters through the analysis. This procedure results in 384 independent variations reflecting the four coefficients of the smoothing polynomials, the two muon charges, and the 48 *η*^μ^ bins. These uncertainties change both the shape and normalization of the ${p}_{{\rm{T}}}^{{\rm{\mu }}}$ distribution in each *η*^μ^ bin.

The prompt-muon contamination in the sideband regions is modelled with simulated events, with all the corrections applied as for the signal region, including the appropriate combination of SFs for events that fail the isolation requirement in the nonisolated sideband regions. All experimental and theoretical systematic uncertainties in the prompt-muon contamination are propagated to the sideband regions by repeating the subtraction of the prompt component and the determination of the smoothing parameters in the sideband regions for each variation. In this way, uncertainties stemming from experimental or theoretical sources are also assigned to the nonprompt-muon background, and the correct correlation structure between prompt- and nonprompt-muon events is consistently taken into account in the uncertainty model.

The extended ABCD method is validated using both simulated nonprompt-muon events from QCD multijet production and a control sample of data enriched in events with nonprompt muons. Simulated background events permit a stringent test of the internal consistency of the method with no signal contamination. However, they might not accurately describe the background processes in data and a complementary data-driven check with the control region is necessary. The control region selects nonprompt muons matched to secondary vertices, which originate from the decay of heavy-flavour hadrons and appear displaced from the beam line in the transverse plane. Other selection criteria are the same as in the signal region. The signal contamination in this sample is below 2%.

We observe that the extended ABCD method overestimates the nonprompt-muon yields in the data control region. This overestimate is also seen in simulated QCD multijet events when comparing the prediction of the extended ABCD method applied to the simulation to the direct prediction in the high-*m*_T_ and low-isolation region. This discrepancy originates from the nonlinear correlation between the nonprompt-muon isolation efficiency and *m*_T_. To account for this effect, a correction is applied to the predicted yields. This correction has a stable value of 85% across different bins of $({p}_{{\rm{T}}}^{{\rm{\mu }}},{\eta }^{{\rm{\mu }}})$ and is used to scale down the overall normalization of the prediction. This value is consistent between data and simulation in the control region and, using simulated events, is also confirmed by testing the method directly in the signal region. A 5% uncertainty is assigned to the predicted background normalization, which covers the largest differences observed when validating the extended ABCD region in the data control region and in simulation.

**Extended Data Fig. 3:**
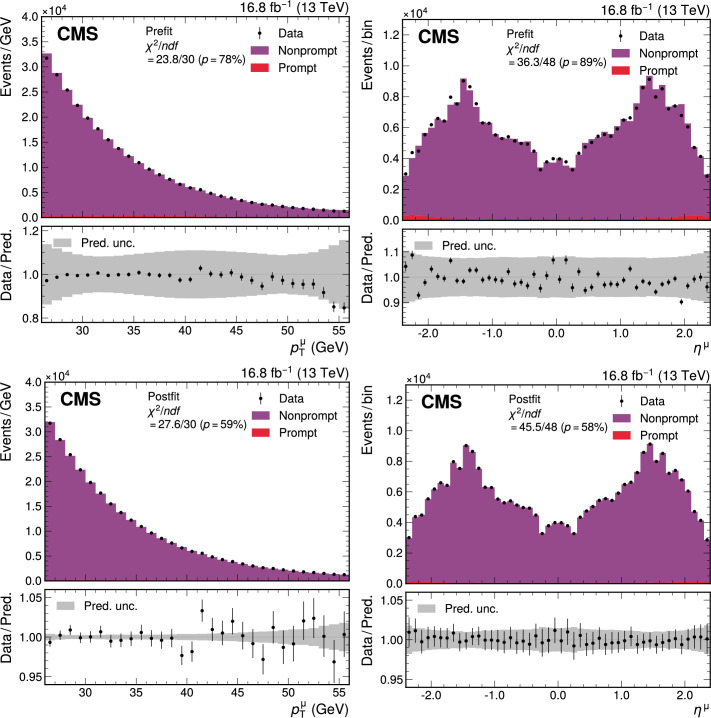
The observed data and the prediction of the extended ABCD method before (top) and after (bottom) the maximum likelihood fit, for the ${{\boldsymbol{p}}}_{{\bf{T}}}^{{\boldsymbol{\mu }}}$ (left) and *η*^μ^ (right) distributions, in a control region enriched in events with nonprompt muons matched to displaced secondary vertices. Small contributions from events with a prompt muon, evaluated using simulated samples, are shown by the red histogram. The total uncertainties (statistical and systematic) are represented by the gray bands. The vertical bars represent the statistical uncertainties in the data. The bottom panel shows the ratio of the number of events observed in data and of variations in the predictions to that of the total nominal prediction.

We test the background uncertainty model by performing maximum likelihood fits to the $({p}_{{\rm{T}}}^{{\rm{\mu }}},{\eta }^{{\rm{\mu }}},{q}^{{\rm{\mu }}})$ distribution in the control region with secondary vertices, both with simulated and data events. To cover residual shape effects, two additional nuisance parameters are assigned to vary the linear and quadratic coefficients of the smoothing polynomial, fully correlated across *η*^μ^ and *q*^μ^ bins. The prefit agreement between the nonprompt-muon background predicted by the extended ABCD method and the data in the control region, after correcting the normalization, is shown in Extended Data Fig. 3. The uncertainty model covers the discrepancies in the shape and normalization, as confirmed by the postfit distributions in Extended Data Fig. 3 and the goodness-of-fit values. Finally, we perform a test with biased pseudo-data directly in the signal region. In this test, the pseudo-data differ from the nominal prediction by the difference between the extended ABCD prediction and the direct prediction in high-*m*_T_ and low-isolation region, evaluated from the QCD multijet simulation. The shift in *m*_W_ from the fit to the biased pseudo-data is within the nonprompt-muon background uncertainty.

The total uncertainty in *m*_W_ from the nonprompt-muon background is 3.2 MeV. It includes the normalization uncertainty (0.2 MeV), the two systematic variations of the coefficients (2.5 MeV) and the statistical uncertainty of the smoothing function coefficients (1.9 MeV). More uncertainties result from variations of the predicted nonprompt-muon background due to experimental and theoretical effects, which modify the prompt-muon contamination that is subtracted from data in the sideband regions. They are accounted for as part of the corresponding experimental and theoretical uncertainties in *m*_W_ from the respective sources. We verified that adopting an alternative smoothing algorithm shifts the observed *m*_W_ by less than the associated uncertainty.

### Muon momentum calibration

The muon tracks are first reconstructed using a standard pattern recognition and Kalman filter track fit^27^. To improve the accuracy of the track parameter determination, the tracks are then refitted using a continuous variable helix (CVH) fit, a global *χ*^2^ fit that extends the generalized broken-line fit^75,76^ to incorporate continuous energy loss and multiple scattering from finite material elements. The detailed material model of the CMS detector used for our simulation is based on the initial design of the tracker material and support structures as well as in situ measurements using collision data, such as ref. ^77^. This model is incorporated into the track fit using the GEANT4e propagator^45,78,79^. To model the magnetic field, we use a parameterization of the detailed three-dimensional solenoidal field map^80^ rather than the less accurate, but computationally faster, finite-element model used in the standard reconstruction. The starting point for the alignment corresponds to what is used in the standard CMS reconstruction^47,81^. As compared with the standard track fit, additional quality criteria are used to select pixel hits, and a refined parameterization of the local hit position is used for the trapezoidal strip modules in the endcaps of the strip detector. To ensure an accurate modelling of track hit positions in the simulation, the numerical precision of the helix-surface intersection in GEANT4 has been increased with respect to the standard CMS simulation. The Kalman filter track fit is used only to associate hits to a given track. The CVH fit is applied to data and simulation, and used to determine all track parameters, including the track momentum.

Although the models used to describe the magnetic field, the material distribution and the detector alignment are the most accurate available at present, a few sources of potential biases remain. The magnetic field model is based on measurements made in the ground-level assembly hall rather than in the cavern and does not account for differences in the field induced by material in the detector and surroundings. The simulation geometry underlying the material model might not provide a perfect description of the real detector, and there are inaccuracies in the Gaussian model used to incorporate material effects. Finally, the alignment is affected by small residual biases in the alignment procedure and by so-called ‘weak modes’ (misalignment patterns, including global translations, twists and radial expansions, that bias the parameter extraction from the track but do not affect the overall *χ*^2^ of the track fits^81^). To correct for these biases, we developed a generalized correction procedure that extends the standard alignment procedure. The alignment degrees of freedom are parameterized by the three translation and three rotation degrees of freedom per tracker module, albeit without extra parameters for module deformation or residual time dependence. The parameterization is extended with additional parameters to correct the *z* component of the magnetic field and the energy loss from material in the vicinity of each module. The correction parameters are derived from a sample of J*/*Ψ → μμ decays using the CVH fit, imposing the additional constraints that the muons are produced from a common vertex and that the muon pair has a mass consistent with that of the J/Ψ meson. These are needed to constrain weak modes in the alignment, magnetic field and energy loss parameters.

The correction procedure is effective in absorbing local biases in the magnetic field, energy loss and alignment, but remains subject to weak modes, as well as to residual biases resulting from limitations in the Gaussian J*/*Ψ meson mass constraint. Convolution effects from the finite detector resolution and for final-state radiation are accounted for only in an approximate manner, and background contributions are not considered. To correct for these potential biases, residual corrections are derived from fits to the J*/*Ψ → μμ dimuon mass distribution in two steps. In the first step, we extract correction factors in fine bins over a four-dimensional space constructed from the ${p}_{{\rm{T}}}^{{\rm{\mu }}}$ and *η*^μ^ of the two muons, to adjust the muon momentum scale in data to that of the simulation. In these fits, the signal model is based on templates from simulation, convolved with a Gaussian whose mean and standard deviation (*σ*) account for the residual scale and resolution difference. The combinatorial background is represented by an exponential function. In the second step, the muon momentum scale correction factors are translated into correction parameters for each individual muon. The conversion between the four-dimensional corrections and the per-muon correction parameters is performed with a *χ*^2^ minimization. The residual corrections to the muon momenta are binned in *η*^μ^ and parameterized as a function of *q*^μ^ and the curvature, *k* ≡ 1/*p*_T_, as

1 $$\frac{\delta k}{k}={A}_{i\eta }-{{\epsilon }}_{i\eta }k+q{M}_{i\eta }/k.$$

The *iη* subscript indicates the corresponding *η* bin of the correction parameters. The parameters are independent for the 48 *η* bins of width 0.1 and are integrated over the *ϕ* coordinate. The *A*_*iη*_ term corresponds to a small adjustment of the magnetic field. The *ϵ*_*iη*_ term is the first one in a Taylor series expansion for the effect of mismodelling the energy loss between the interaction point and the first hit measurement. The *M*_*iη*_ term expresses the bias in the track sagitta resulting from a misalignment of the tracker in the plane transverse to the magnetic field. The expression captures the leading behaviour once local biases in the magnetic field, material and alignment are corrected. In the presence of sufficiently large local biases, additional terms would appear with a more complicated functional form. Using MC simulation, we have validated that residual biases are well described by this functional form, after performing the track refit and applying the generalized global corrections. The corrections are then applied by shifting the reconstructed curvature of the measured muons. Illustrative fits to the dimuon mass distribution in J/Ψ → μμ events are shown in Supplementary Fig. 17, and the parameters of equation (1) extracted from the fits to data are shown in Supplementary Fig. 18.

To avoid extrapolating the muon momentum resolution corrections from the relatively low momentum values typical of muons from J/Ψ decays, we calibrate the muon momentum resolution using both J/Ψ → μμ and Z → μμ events. The resolution corrections are derived from fits to the J/Ψ → μμ and Z → μμ dimuon mass distributions, binned in the ${p}_{{\rm{T}}}^{{\rm{\mu }}}$ and *η*^μ^ of the positively and negatively charged muon as for the scale corrections, and after correcting the momentum scale using the calibration parameters previously extracted from the J*/*Ψ sample. The resolution is parameterized as a function of the curvature as

2 $$\frac{{\sigma }_{k}^{2}}{{k}^{2}}={a}_{i\eta }^{2}+\frac{{c}_{i\eta }^{2}}{{k}^{2}}+\frac{{b}_{i\eta }^{2}}{1+{d}_{i\eta }^{2}{k}^{2}},$$

where the parameters *a*_*iη*_, *c*_*iη*_, *b*_*iη*_ and *d*_*iη*_ parameterize the contributions to the curvature resolution from multiple scattering, hit resolution and the correlations between them induced by the track fit. These parameters are computed in 24 *η* bins of width 0.2 for each of the four terms, separately for data and simulation, and are applied by smearing the reconstructed curvature of the simulated muons through a Gaussian distribution with the width corresponding to the difference in quadrature between data and simulation.

**Extended Data Fig. 4:**
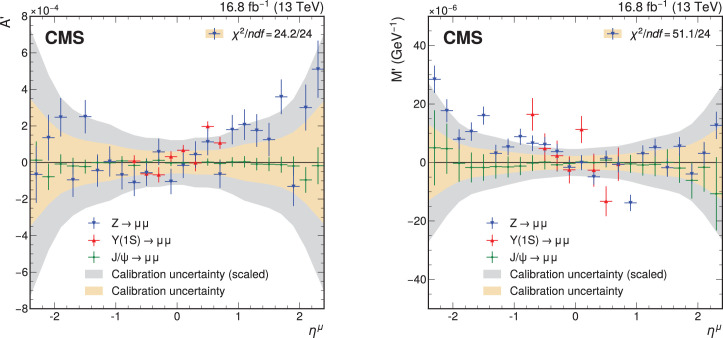
Charge-independent (${{\boldsymbol{A}}}^{{\prime} }$, left) and charge-dependent (${{\boldsymbol{M}}}^{{\prime} }$, right) residual scale differences between data and simulation, after applying the corrections derived from the J/*Ψ* → μμ event sample. The scale differences are evaluated separately in J/*Ψ* → μμ, **Y**(1S) → μμ, and Z → μμ events to assess the consistency of the muon momentum scale calibration in the different event samples. The charge-independent comparison probes a magnetic-field-like difference, whereas the charge-dependent comparison reflects a misalignment-like term. The points with error bars represent the scale parameters and statistical uncertainties associated with the closure test performed with J/*Ψ* → μμ (green), **Y**(1S) → μμ (red), and Z → μμ (blue) events. The yellow band represents the corresponding statistical uncertainty in the calibration parameters derived from the J/*Ψ* calibration sample. The filled gray band shows this uncertainty scaled by a factor of 2.1, as described in the text. The *χ*^2^ values correspond to the compatibility of the scale parameters with zero for the closure test performed with Z → μμ events. The statistical uncertainties for these parameters, as well as for the calibration parameters derived from the J/*Ψ* sample (without the 2.1 uncertainty scaling factor applied), are taken into account. The calibration parameter uncertainties are fully uncorrelated from the Z and **Y**(1S) closure test uncertainties, but very strongly correlated with the J/*Ψ* closure uncertainties, since they use the same data.

The calibration is validated using J*/*Ψ → μμ, **Y**(1S) → μμ, and Z → μμ events, by computing the residual muon momentum scale difference between the measured and simulated distributions, after applying all corrections, following the same two-step procedure used to derive the calibration factors. The residual scale differences between the event samples are obtained in 24 bins of *η*^μ^ following the parameterization of equation (1) with *ϵ*_*iη*_ = 0. The resulting closure parameters, corresponding to a charge-independent magnetic-field-like residual (*A*′) and a charge-dependent alignment-like residual (*M*′), are shown in Extended Data Fig. 4. The *χ*^2^ compatibility test for the J/Ψ → μμ calibration applied to Z → μμ events demonstrates that there is consistency within the statistical uncertainties for the charge-independent residuals. A small inconsistency for the charge-dependent residuals is seen, indicating a systematic uncertainty source. Given the momentum range relevant for W → μν events, the magnetic field and alignment effects are dominant with respect to those reflecting energy loss. The **Y**(1S) → μμ events are used only to validate the calibration in the central region of the detector, in which the dimuon mass resolution allows us to select a high purity sample of muons from the **Y**(1S) meson decay. The small deviations from zero in the J*/*Ψ → μμ events are due to the larger *η*^μ^ bin sizes used for the validation step and from small ${p}_{{\rm{T}}}^{{\rm{\mu }}}$ bin migrations after applying the initial corrections in the *A*′ and *M*′ parameter extraction. The differences are small compared with the statistical uncertainty in the calibration procedure.

The uncertainties propagated to the analysis include the statistical uncertainties in the calibration parameters extracted from the J*/*Ψ sample, with statistical correlations taken into account, as well as the statistical uncertainties in the residual nonclosure between the J/Ψ and the Z samples and the systematic uncertainty associated with the reference measurement of the Z boson mass^1^. Although these uncertainties account for the limited size of the measured and simulated event samples in the J*/*Ψ calibration procedure and closure tests, as well as for the uncertainty in the world-average Z boson mass, other systematic effects might be present, related to weak modes with different sensitivity in J*/*Ψ → μμ and **Y**(1S) → μμ events, trigger biases or other sources. Remaining systematic effects that are not explicitly accounted for are assessed from the closure test between the J*/*Ψ calibration and the momentum scale from the Z sample. The statistical compatibility of this test is assessed for different *η*^μ^ binning choices and considering several possible correlated patterns of biases. To cover all possible biases with a reduced *χ*^2^ smaller than unity, the statistical uncertainty in the J*/*Ψ calibration parameters is scaled by a factor of 2.1, as shown in Extended Data Fig. 4.

For the momentum resolution, the relative agreement between the measured and simulated samples, especially in the tails of the momentum response distribution, is affected by a different pixel hits efficiency after the tighter quality requirements imposed in the CVH fit. To account for this, a systematic uncertainty is evaluated by reweighting the simulated pixel hit multiplicity distribution to match data differentially in *η*^μ^ and taking the full difference as an uncertainty. As the nominal resolution corrections are also affected by this issue, we assign a systematic uncertainty to cover the residual disagreement. This uncertainty is expressed in terms of the statistical uncertainty of the resolution correction parameters, which are scaled by a factor of 10 to cover the observed differences. Because the statistical uncertainty in the resolution correction is small, and because the *m*_W_ measurement is not sensitive to small changes in the resolution, these scaled resolution uncertainties contribute only 1.4 MeV to the uncertainty in *m*_W_.

The uncertainties in ${p}_{{\rm{T}}}^{{\rm{\mu }}}$ from the momentum scale and resolution calibrations are several orders of magnitude smaller than the 1 GeV ${p}_{{\rm{T}}}^{{\rm{\mu }}}$ bin width of our likelihood function. If the impact of the ${p}_{{\rm{T}}}^{{\rm{\mu }}}$ variation is evaluated using event-level shifts in ${p}_{{\rm{T}}}^{{\rm{\mu }}}$, the $({p}_{{\rm{T}}}^{{\rm{\mu }}},{\eta }^{{\rm{\mu }}},{q}^{{\rm{\mu }}})$ template shapes are determined only by events in which the varied ${p}_{{\rm{T}}}^{{\rm{\mu }}}$ is assigned to a different histogram bin than its nominal value. The probability of this bin migration depends on the relative size of the ${p}_{{\rm{T}}}^{{\rm{\mu }}}$ shift compared with the bin width, which is ${\mathcal{O}}(1{0}^{-4})$ for a typical selected muon. As a result, the effective number of events contributing to the template shapes is very small, which leads to large statistical fluctuations in the uncertainty templates. To avoid this issue, the variations are evaluated by reweighting events in terms of the muon momentum response distribution, resulting in smooth variation templates. The breakdown of muon momentum calibration uncertainties is shown in Extended Data Table 1. The total contribution of the muon momentum calibration to the *m*_W_ uncertainty is 4.8 MeV. This uncertainty has been validated by applying the difference in scale between the J/Ψ → μμ and Z → μμ events to the W boson simulation to build a biased prediction that is tested as pseudo-data in the fit. The resulting shift in *m*_W_ from this procedure is covered by the corresponding calibration uncertainties. Supplementary Fig. 1 shows the Z → μμ dimuon mass distributions after correcting the muon momentum scale by the calibration parameters extracted from fits to the J*/*Ψ events.

### Modelling of the W and Z boson transverse momentum distributions

To achieve the best accuracy in modelling the ${p}_{{\rm{T}}}^{{\rm{V}}}$ spectra, we correct the generator-level ${p}_{{\rm{T}}}^{{\rm{V}}}$ and *y*^V^ distributions in MINNLO_PS_ to state-of-the-art calculations in QCD, including the resummation of logarithmically enhanced contributions at small ${p}_{{\rm{T}}}^{{\rm{V}}}$ and a model for nonperturbative effects also at small ${p}_{{\rm{T}}}^{{\rm{V}}}$. We use the SCETLIB code^30,31,43^, which performs ${p}_{{\rm{T}}}^{{\rm{V}}}$ resummation as formulated using soft-collinear effective theory (SCET)^82–84^, using deterministic numerical integration routines to provide predictions with high numerical accuracy. The resummed predictions from SCETLIB are matched to the fixed-order calculation from DYTURBO^50^, at $O({\alpha }_{s}^{2})$ in the QCD coupling constant *α*_*s*_, to achieve N^3^LL + NNLO accuracy. The correction is derived from the ratio of the SCETLIB + DYTURBO and MINNLO_PS_ predictions for a fixed value of *m*_V_ (after the parton shower but before final-state photon radiation) in the full phase space of the decay lepton kinematics. The corrections are binned in 1 GeV bins of ${p}_{{\rm{T}}}^{{\rm{V}}}$, up to 100 GeV, and 0.25 wide bins in the ∣*y*^V^∣ < 5.0 range. They are applied to the MINNLO_PS_ simulation by sampling the binned corrections per event with the generator-level ∣*y*^V^∣ and ${p}_{{\rm{T}}}^{{\rm{V}}}$ to obtain event-level weights that are propagated through the full experimental analysis. This procedure allows us to maintain the statistical power of the MINNLO_PS_ MC sample while improving its accuracy at small ${p}_{{\rm{T}}}^{{\rm{V}}}$. After the correction, the dependence of the ${p}_{{\rm{T}}}^{{\rm{V}}}$ distribution on the parton shower and tune is negligible. We have compared the predictions using SCETLIB + DYTURBO with those using DYTURBO v.1.4.0 (refs. ^50,85^), MATRIX + RADISH v.1.0.0 (refs. ^48,86^) and CuTe-MCFM v10.2 (refs. ^51,87^), at equivalent or higher perturbative order. After propagating those predictions through the analysis as binned corrections in ${p}_{{\rm{T}}}^{{\rm{V}}}$, we find that the expected shifts in *m*_W_ are within the SCETLIB + DYTURBO uncertainties.

**Extended Data Fig. 5:**
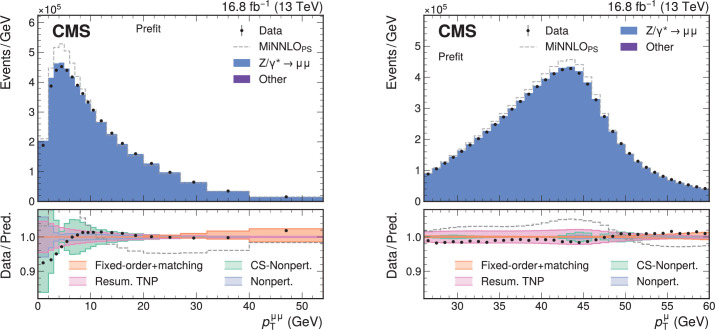
Measured and simulated ${{\boldsymbol{p}}}_{{\bf{T}}}^{{\boldsymbol{\mu \mu }}}$ (left) and ${{\boldsymbol{p}}}_{{\bf{T}}}^{{\boldsymbol{\mu }}}$ (right) distributions in selected Z → μμ events. The standalone uncorrected MINNLO_PS_ predictions are shown by the dashed gray line. The nominal predictions (blue) correct the POWHEG ${{\rm{MiNNLO}}}_{{\rm{PS}}}\,{p}_{{\rm{T}}}^{{\rm{V}}}$ with SCETLIB + DYTURBO at N^3^LL + NNLO, as described in the text. The vertical bars represent the statistical uncertainties in the data. The bottom panel shows the ratio of the number of events observed in data to that of the total nominal prediction, as well as the relative impact of variations of the predictions. Different sources of uncertainty are shown as solid bands in the lower panel: the fixed-order uncertainty and the uncertainty in the resummation and fixed-order matching (orange), resummed prediction using TNPs (pink), the Collins–Soper (CS) kernel nonperturbative uncertainty (green), and other nonperturbative uncertainties (light blue). Additional sources of experimental and theoretical uncertainty that impact the agreement with the data are not shown.

As shown in Extended Data Fig. 5, the SCETLIB + DYTURBO correction substantially improves the description of ${p}_{{\rm{T}}}^{{\rm{\mu \mu }}}$ and ${p}_{{\rm{T}}}^{{\rm{\mu }}}$ data in selected Z → μμ events when compared with the standalone MINNLO_PS_ predictions. Uncertainties in the ${p}_{{\rm{T}}}^{{\rm{W}}}$ prediction, particularly those impacting the low-${p}_{{\rm{T}}}^{{\rm{W}}}$ region, can shift the peak of the ${p}_{{\rm{T}}}^{{\rm{\mu }}}$ distribution in a way similar to a variation of *m*_W_. Therefore, the sensitivity of the analysis to *m*_W_ critically relies on differentiating the uncertainty in ${p}_{{\rm{T}}}^{{\rm{W}}}$ and its impact on the ${p}_{{\rm{T}}}^{{\rm{\mu }}}$ distribution from *m*_W_ variations. As can be appreciated from Extended Data Fig. 5, different sources of uncertainty contribute predominantly to different ${p}_{{\rm{T}}}^{{\rm{V}}}$ regions. The nonperturbative uncertainty is most pronounced at low ${p}_{{\rm{T}}}^{{\rm{V}}}$. Uncertainties in the resummation calculation and in the matching of the resummed and fixed-order calculations are relevant up to ${p}_{{\rm{T}}}^{{\rm{V}}}\approx 40\,{\rm{GeV}}$. The nonperturbative and resummation uncertainties are most pronounced near the peak of the ${p}_{{\rm{T}}}^{{\rm{\mu }}}$ distribution, the region most sensitive to the *m*_W_ value. Consequently, their contributions have an important impact on the measurement of *m*_W_. The perturbative uncertainties in fixed-order QCD, which are dominant at high ${p}_{{\rm{T}}}^{{\rm{V}}}$, have a small impact on ${p}_{{\rm{T}}}^{{\rm{\mu }}}$ in the region sensitive to *m*_W_. The uncertainties are estimated by varying the relevant parameters of the SCETLIB + DYTURBO calculation to obtain alternative predictions that are propagated through the full experimental analysis via event-level weights.

**Extended Data Fig. 6:**
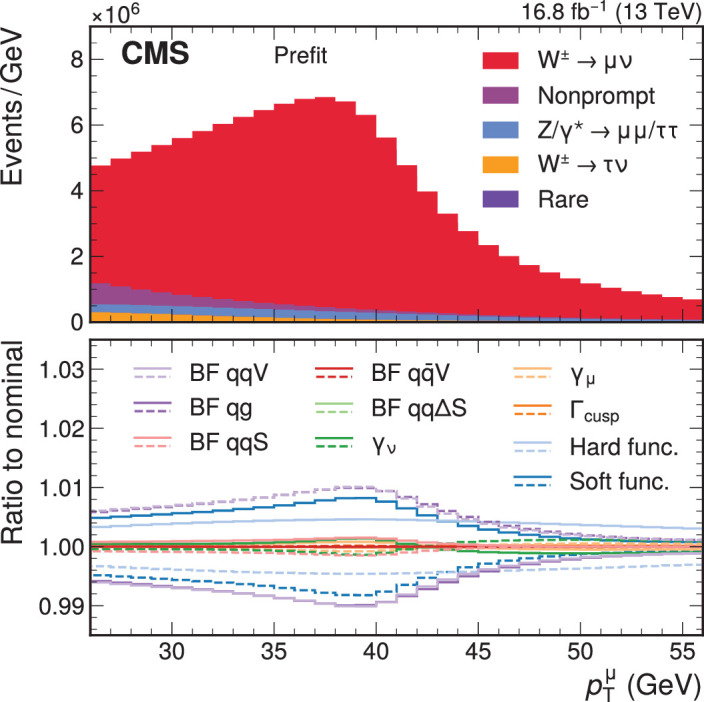
The predicted ${{\boldsymbol{p}}}_{{\bf{T}}}^{{\boldsymbol{\mu }}}$ distribution for selected W → μ***ν*** events, before the maximum likelihood fit. The lower panel shows the ratio of the ten TNP variations described in the text to the nominal prediction, illustrating their impact on the spectrum.

Perturbative uncertainties in the resummed predictions are evaluated using the TNP approach recently proposed in ref. ^32^, which exploits the known all-order perturbative structure of the resummed calculation and is implemented in SCETLIB. In the SCET formalism used here, there are three perturbative ingredients in the ${p}_{{\rm{T}}}^{{\rm{V}}}$ resummation: the ‘hard function’ that describes the hard virtual corrections for W and Z production, the ‘proton beam functions’ that extend the PDFs to include collinear radiation, and the ‘soft function’ describing soft radiation. All these functions share a system of renormalization group equations whose solution yields the all-order resummation of logarithms of ${p}_{{\rm{T}}}^{{\rm{V}}}/{m}_{{\rm{V}}}$. In the TNP approach, the minimal independent set of ingredients that would be needed at the next perturbative order are identified and parameterized in terms of common nuisance parameters. Specifically, there are six sources of TNPs: the three fixed-order boundary conditions of each of the hard (H), soft (S) and beam (B) functions, and three anomalous dimensions governing their renormalization group evolution, namely, the cusp anomalous dimension (*Γ*_cusp_) and the virtuality and rapidity noncusp anomalous dimensions (*γ*_μ_ and *γ*_ν_). The TNPs of the hard and soft functions and the three anomalous dimensions are numerical constants. As such, they are propagated as scalar variations around their known values. The beam functions (BF) comprise five one-dimensional functions of the Bjorken-*x* for the different partonic splitting channels. The qqV and qg BF contain the dominant quark to quark (q → q) and gluon to quark (g → q) channels, whereas the others (${\rm{q}}\bar{{\rm{q}}}{\rm{V}}$, qqS and qqΔS) correspond to specific nondiagonal q → q′ contributions that are present at higher orders^43^. We use their known functional shape and treat their normalization as a scalar TNP for each partonic channel. The TNPs have a true, but unknown, value that can be varied according to their expected typical magnitude. As a result, the TNP approach provides a robust prediction for the correlation of the uncertainties because of the missing higher orders across ${p}_{{\rm{T}}}^{{\rm{V}}}$, *y*^V^ and *m*_V_, which can be consistently used in the profile maximum likelihood fit used to extract *m*_W_. Extended Data Fig. 6 shows the impact of the 10 TNPs, propagated through the analysis using the event-weighting procedure, on the ${p}_{{\rm{T}}}^{{\rm{\mu }}}$ spectrum in W → μν events.

The SCETLIB program implements different configurations for the TNPs, in terms of the logarithmic accuracy of the prediction and the perturbative order at which the TNPs are included. The order of the calculation is expressed following the notation in ref. ^32^. An N^*m*+*k*^LL prediction is built from a full calculation of the first *m* logarithmic terms in the resummation series, with a further *k* terms with unknown coefficients used to estimate the theory uncertainty. We use the N^3+0^LL scheme, in which the prediction has N^3^LL accuracy and the TNPs representing the unknown HO corrections are estimated from multiplicative variations of the same N^3^LL terms. We also consider two alternate schemes, N^3+1^LL and N^4+0^LL. The N^3+1^LL scheme implements the full N^4^LL perturbative structure, combining the known values for the parameters up to N^3^LL with best estimates of the HO terms and their variations to define the TNP variations. The N^4+0^LL scheme follows the same approach as the N^3+0^LL, but it is based on the N^4^LL calculation. We have verified that using the N^3+1^LL or the N^4+0^LL schemes has a negligible impact on the results with respect to the nominal N^3+0^LL scheme. As discussed in sections ‘Additional validation of theoretical modelling’ and ‘The W-like Z and W boson mass measurements’, we validate the robustness of this approach against the measured ${p}_{{\rm{T}}}^{{\rm{\mu \mu }}}$ distribution and the W-like measurement of *m*_Z_, in which the impact of the ${p}_{{\rm{T}}}^{{\rm{Z}}}$ modelling uncertainty is treated in the same way.

The perturbative uncertainty in the fixed-order matching correction of the unpolarized calculation is assessed from 7-point variations of the factorization and renormalization scales, μ_R_ and μ_F_, in the DYTURBO calculation. The uncertainty is correlated for the different W (and Z) boson decay channels, and between W^+^ and W^−^ boson production, but uncorrelated between W and Z boson production. This uncertainty is profiled in the maximum likelihood fit. If the uncertainty is excluded from the profiling procedure and estimated by repeating the maximum likelihood fit after varying μ_R_ and μ_F_, or if the variations are correlated between W and Z boson production, the shift in the measured *m*_W_ value is <0.4 MeV. The impact of both choices on the total uncertainty in *m*_W_ is negligible. An uncertainty due to the matching procedure is evaluated by varying the transition scale (the midpoint of the transition function defined in ref. ^31^) between the resummation and the fixed-order regime from the nominal value of 0.5 *m*_V_ to 0.35 *m*_V_ and 0.75 *m*_V_.

Nonperturbative effects, such as the residual transverse motion of the partons inside the proton, affect the ${p}_{{\rm{T}}}^{{\rm{V}}}$ distribution. These effects are expected to scale as ${({\varLambda }_{\mathrm{QCD}}/{p}_{{\rm{T}}}^{{\rm{V}}})}^{2}$ ^88^, where *Λ*_QCD_ ≈ 200 MeV is the QCD vacuum expectation value. As such, their impact is dominant at low ${p}_{{\rm{T}}}^{{\rm{V}}}$ and less relevant for ${p}_{{\rm{T}}}^{{\rm{V}}}\gtrsim 10\,\mathrm{GeV}$. The predictions considered here implement phenomenological models that require tuning to data to describe these nonperturbative effects. Two sources of nonperturbative effects affect ${p}_{{\rm{T}}}^{{\rm{V}}}$. First, there can be nonperturbative corrections to the Collins–Soper (CS) rapidity anomalous dimension^88^, which are universal for W and Z boson production. Second, there are nonperturbative contributions to the beam (and soft) functions, which account for the intrinsic *k*_T_ of the partons inside the protons, that are not universal as they can depend on the flavour and Bjorken-*x* of the interacting parton. As shown in ref. ^89^, the leading nonuniversal dependence can be captured by a single effective model function that depends only on the vector boson rapidity for each given vector boson type, apart from the helicity cross-section and the collision centre-of-mass energy. The SCETLIB program implements a corresponding nonperturbative model for both these sources^31^, in which the model parameters effectively determine the first two powers in an expansion in ${({\varLambda }_{\mathrm{QCD}}/{p}_{{\rm{T}}}^{{\rm{V}}})}^{2}$ together with a parameter that determines the overall asymptotic behaviour for ${p}_{{\rm{T}}}^{{\rm{V}}}\to 0$. For the intrinsic *k*_T_, the effective model amounts to a (rapidity-dependent) Gaussian smearing in the Fourier conjugate of ${p}_{{\rm{T}}}^{{\rm{V}}}$.

In our analysis, the five parameters of the SCETLIB model are loosely constrained around nominal values that correspond to minimal nonperturbative smearing. The CS anomalous rapidity parameters are correlated between Z and W boson production, whereas the Gaussian smearing terms are uncorrelated between the two. Their best-fit values are extracted from the maximum likelihood fits to the measured distributions. The values obtained from a direct maximum likelihood fit to the ${p}_{{\rm{T}}}^{{\rm{\mu \mu }}}$ distribution are consistent with those resulting from the W-like fit to the ${p}_{{\rm{T}}}^{{\rm{\mu }}}$ distribution in Z → μμ events.

The SCETLIB + DYTURBO and MINNLO_PS_ calculations are performed in a fixed-flavour scheme with massless quarks. Calculations with b and c quark masses have not been performed with a comparable perturbative accuracy. The impact of quark masses is expected to be mostly at the scale of the b and c masses. Their impact is partially estimated by varying the heavy-quark thresholds using charm and bottom quark mass variations of the MSHT20 PDF set^90^. Moreover, the loose initial constraints of our nonperturbative uncertainty model provide flexibility to cover these sources of uncertainty at low ${p}_{{\rm{T}}}^{{\rm{V}}}$. Quark mass effects are expected to differ between W and Z production because of the different flavour contributions to their production. This difference is captured by the variations of the PDF threshold and by our independent treatment of nonperturbative uncertainties between Z and W production. The sufficiency of our model to capture these effects is confirmed by the likelihood fits to data discussed in section ‘Additional validation of theoretical modelling’.

The total impact on *m*_W_ from the perturbative, nonperturbative and quark mass threshold uncertainties is 2.0 MeV, the three components yielding comparable contributions. A summary of nuisance parameters in the maximum likelihood fit that represent these uncertainties is given in Supplementary Table 2. Section ‘Additional validation of theoretical modelling’ further discusses the validation of the model and its uncertainty.

### Modelling of the angular distributions in W and Z boson leptonic decays

The differential cross-section for the production and decay of the spin-1 W and Z bosons can be decomposed in terms of spherical harmonics into nine helicity-dependent states^34^,

3 $$\frac{{\rm{d}}\sigma }{{\rm{d}}{p}_{{\rm{T}}}^{2}{\rm{d}}m{\rm{d}}y\,{\rm{d}}\cos {\theta }^{* }{\rm{d}}{\phi }^{* }}=\frac{3}{16{\rm{\pi }}}\frac{{\rm{d}}{\sigma }^{{\rm{U}}+{\rm{L}}}}{{\rm{d}}{p}_{{\rm{T}}}^{2}{\rm{d}}m{\rm{d}}y}\left[(1+{\cos }^{2}{\theta }^{* })+\mathop{\sum }\limits_{i=0}^{7}{A}_{i}({p}_{{\rm{T}}},m,y){P}_{i}(\cos {\theta }^{* },{\phi }^{* })\right].$$

We choose the CS reference frame^91^, where $\cos {\theta }^{* }$ and ϕ* correspond to the polar and azimuthal angles, respectively, of the muon emitted in the W boson decay. The angular coefficients *A*_*i*_ depend on the boson charge, rapidity *y*^V^, ${p}_{{\rm{T}}}^{{\rm{V}}}$ and *m*_V_. Combined with the unpolarized cross-section *σ*^U+L^, they describe the relationship between the boson production and the kinematic distributions of the decay muons. The *P*_*i*_ spherical harmonics describe the kinematic distributions of the daughter muon, which depend on the properties of the W or Z boson.

The nominal predictions for the angular distributions, from MINNLO_PS_, are NNLO accurate in QCD. Uncertainties in the predicted angular coefficients impact the ${p}_{{\rm{T}}}^{{\rm{\mu }}}$ and *η*^μ^ distributions by modifying the polarization of the W boson. Uncertainties in the angular coefficients are assessed by varying μ_R_ and μ_F_ in the MINNLO_PS_ predictions. The correlations of HO corrections across phase space and processes are not well known. Therefore, we consider these variations uncorrelated among the *A*_*i*_ coefficients and in 10 ${p}_{{\rm{T}}}^{{\rm{V}}}$ bins, but correlated across *y*^V^, and between W^+^, W^−^ and Z.

We have verified that the MINNLO_PS_ predictions and uncertainties for the angular coefficients are consistent with NNLO fixed-order calculations, and that the *A*_*i*_ coefficients predicted at N^3^LL, assessed with both SCETLIB and DYTURBO, are consistent with the MINNLO_PS_ predictions within the assigned uncertainties. We have validated that the *A*_*i*_ coefficients predicted by MINNLO_PS_ are consistent with those measured in data for Z boson production. Measurements of the *A*_*i*_ coefficients for W boson production recently reported by the ATLAS Collaboration^92^ confirm the accuracy of the NNLO predictions. The isotropic smearing of the colliding partons due to the intrinsic *k*_T_ model of PYTHIA induces a modest change to the angular coefficients, in particular *A*_1_ and *A*_3_ at low W or Z boson transverse momentum. The full difference between the angular coefficients before and after the PYTHIA 8 shower and intrinsic *k*_T_ is taken as an additional systematic uncertainty, fully correlated across angular coefficients, phase space, and W and Z boson production. The total uncertainty in *m*_W_ due to the uncertainty in the predicted angular coefficients is 3.2 MeV, with the largest contributions coming from *A*_0_, *A*_2_ and *A*_4_. If the uncertainty in the angular coefficients defined by variations of μ_R_ and μ_F_ is excluded from the profiling procedure and estimated by repeating the maximum likelihood fit for each variation, the measured value of *m*_W_ shifts by +2.5 MeV. This increases the uncertainty in the angular coefficients to 3.9 MeV.

### Parton distribution functions

Supplementary Fig. 2 shows the *η*^μ^ distribution for W^+^ and W^−^ boson events, compared with the predictions obtained with the CT18Z PDF set and its uncertainties, as well as with the central predictions for several other PDF sets. The consistency among the five PDF sets and the observed data is determined by performing likelihood fits to these distributions for each PDF set under consideration. Fits are performed including all the uncertainties of the nominal *m*_W_ fit, as well as removing the PDF + *α*_*s*_ or the theory uncertainties. The impact of *α*_*s*_ is evaluated from alternative PDF fits with *α*_*s*_ shifted by ±0.015 from its nominal value of 0.118. The change of *α*_*s*_ is propagated through the matrix-element calculation in MINNLO_PS_ and the SCETLIB + DYTURBO corrections. The corresponding saturated likelihood goodness-of-fit values are reported in Supplementary Table 1.

To test the dependence of the result on the choice of PDF set, we performed the *m*_W_ measurement with the NNPDF3.1 (ref. ^64^), NNPDF4.0 (ref. ^65^), CT18 (ref. ^44^), MSHT20 (ref. ^66^) and PDF4LHC21 (ref. ^67^) sets at NNLO, and the approximate N^3^LO set MSHT20aN3LO^68^. To assess the consistency of the PDF sets we perform studies in which the MC simulation for the W and Z boson production, and the corresponding PDF uncertainties, are obtained from a given PDF set, whereas another PDF set provides pseudo-data. We then evaluate if the *m*_W_ value extracted from the fit lies within the uncertainty predicted by the PDF set under test. In the case of the CT18Z, CT18 and PDF4LHC PDF sets, their uncertainty covers the *m*_W_ value extracted with all other PDF sets. This does not happen for the remaining PDF sets and, hence, we test the impact of increasing their PDF uncertainty by scaling all eigenvectors by a constant value until the difference in the extracted *m*_W_ is within the postfit *σ*_PDF_. The SFs determined with this procedure are reported in Extended Data Table 3, which also shows that the total *m*_W_ (unscaled) uncertainty due to the alternative PDF sets ranges from 2.4 MeV to 4.6 MeV. The scaling of the PDF uncertainty has only a moderate impact on the total *m*_W_ uncertainty. Results for each PDF set, obtained with and without the scaling factors, are reported in section ‘Results with alternative parton distribution functions’. When the PDF uncertainty is increased, the degree to which the fit is constrained to the predictions of the global PDF fit is relaxed, and the relative importance of this dataset is increased, leading to a better consistency between the measured *m*_W_ values for different PDF sets. Given its agreement with data, relatively large uncertainty and consistency with the other PDF sets, we select the CT18Z PDF set for the nominal prediction. The PDF uncertainty in *m*_W_ from the CT18Z set is 4.4 MeV.

### Impact of missing higher-order electroweak corrections

By interfacing MINNLO_PS_ with PHOTOS++, QED final-state radiation (FSR) is considered at LL accuracy, including matrix-element corrections and the effect of lepton pair production. The uncertainty in the QED FSR modelling is evaluated by comparing the predictions of MINNLO_PS_ + PHOTOS++ to the prediction in which the matrix-element corrections of PHOTOS++ are switched off. Furthermore, we evaluate the difference between the MINNLO_PS_ + PHOTOS++ prediction and the prediction from HORACE v.3.2 (refs. ^93,94^) with the QED FSR modelled at LL in the collinear approximation. For each comparison, the difference between the two predictions is evaluated from the two-dimensional distribution of the *p*_T_ and mass of the dilepton system, with the charged lepton momentum defined after FSR. This difference is applied to the nominal MINNLO_PS_ + PHOTOS++ prediction and propagated through the analysis as a systematic uncertainty. The impact on *m*_W_ is <0.3 MeV.

The QED initial-state radiation (ISR) is modelled by the PYTHIA 8 parton shower at LL accuracy. The uncertainty is evaluated by comparing to a sample with ISR photon radiation switched off. The modifications on the ${p}_{{\rm{T}}}^{{\rm{V}}}$ and *y*^V^ distributions are propagated through the analysis and found to have a negligible impact on *m*_W_. Apart from the photonic corrections, we consider the impact of EW virtual corrections. For the neutral-current Drell–Yan process, the separation between weak and photonic corrections can be performed in a gauge-invariant way. The virtual EW corrections are calculated at NLO with the Z_ew process in the POWHEG-BOX-V2 (rev. 3900) program^95,96^, including universal HO corrections. The ratios between the NLO + HO EW and LO EW predictions of the Z boson mass, and of the rapidity and $\cos {\theta }^{* }$ distributions, are used to define a systematic variation to the nominal MINNLO_PS_ prediction.

For W boson production, the splitting into virtual and photonic corrections is not gauge invariant and is, hence, ambiguous. Nonetheless, it is possible to separate the two contributions, to reproduce the QED FSR given by PHOTOS++. This separation is implemented in ReneSANCe 1.3.11 (ref. ^97^), and the uncertainty in weak virtual corrections is defined as the ratios between the NLO + HO EW and LO EW predictions of the W boson mass, and of the rapidity and $\cos {\theta }^{* }$ distributions. We cross-checked that the full NLO EW corrections (QED plus weak) agree at the 0.3% level between the POWHEG-BOX-V2^98^ and the ReneSANCe programs, also confirming previous benchmarks^99^. The uncertainty from the virtual EW corrections has an impact on *m*_W_ of 1.9 MeV.

### Additional validation of theoretical modelling

To validate the uncertainties in our theoretical predictions and to quantify the sensitivity of our result to alternative ${p}_{{\rm{T}}}^{{\rm{V}}}$ modelling approaches, we performed several additional checks to demonstrate the stability of the results when modifying the treatment of theoretical predictions and their uncertainties in the analysis. To facilitate this, we correct our dimuon data sample for the effect of the detector response by unfolding the two-dimensional $({p}_{{\rm{T}}}^{{\rm{\mu \mu }}},{y}^{\mathrm{\mu \mu }})$ distribution, extending the study reported in ref. ^63^. The Z/γ* → μμ production cross-section is extracted inclusively in the kinematics of the decay muons, defined before final-state photon radiation, and for 60 < *m*_Z_ < 120 GeV, ${p}_{{\rm{T}}}^{{\rm{Z}}} < 54\,{\rm{GeV}}$, and ∣*y*^Z^∣ < 2.5. The unfolding is performed by a maximum likelihood fit to the two-dimensional distribution of $({p}_{{\rm{T}}}^{{\rm{\mu \mu }}},{y}^{\mathrm{\mu \mu }})$ without regularization. The unfolded ${p}_{{\rm{T}}}^{{\rm{Z}}}$ and ∣*y*^Z^∣ distributions, shown in Fig. 2 and Supplementary Fig. 3, are obtained by integrating over the other dimension of the measured two-dimensional distribution.

We have repeated the W-like *m*_Z_ and *m*_W_ measurements using predictions from SCETLIB at different perturbative orders, matched to DYTURBO, as well as different approaches to incorporate the TNPs. When using N^3+1^LL and N^4+0^LL predictions and uncertainties, the measured value of *m*_W_ is shifted by less than the 2.0 MeV of total ${p}_{{\rm{T}}}^{{\rm{W}}}$-modelling uncertainty of the nominal result. Although the approximate N^4^LL predictions^31^ give slightly reduced ${p}_{{\rm{T}}}^{{\rm{W}}}$ uncertainties, the difference in the total uncertainty in *m*_W_ is negligible when compared with the nominal result. Moreover, we have tested the impact of extracting *m*_W_ with an uncertainty model that corresponds to a simplified and more constrained version of the helicity fit rather than relying on the SCETLIB TNPs. In this approach, the ${p}_{{\rm{T}}}^{{\rm{V}}}$ modelling uncertainty is defined by varying all scales in the SCETLIB calculation independently and taking the envelope of the variations (the maximum per bin of the distribution) as uncertainty. This uncertainty is uncorrelated across 10 quantiles of ${p}_{{\rm{T}}}^{{\rm{V}}}$, such that the model is sufficiently flexible to describe the true ${p}_{{\rm{T}}}^{{\rm{V}}}$ distribution in data. The *m*_W_ value measured with this configuration is shifted by <0.2 MeV with respect to the nominal result.

To further assess the impact of missing HO corrections, we have performed the analysis with the SCETLIB calculation matched to the $O({\alpha }_{s}^{3})\,{{\rm{N}}}^{3}{\rm{LO}}$ predictions from NNLOJET^100,101^. The SCETLIB + NNLOJET predictions, using the CT18Z NNLO PDF set, are introduced into the analysis by two-dimensional corrections with the same procedure as for SCETLIB + DYTURBO. Given the very high complexity of the calculation, the numerical precision of the results is significantly worse than that of the MINNLO_PS_ MC sample, and applying this correction introduces non-negligible fluctuations in the predicted templates. These fluctuations are partially accounted for by propagating the statistical uncertainty of the SCETLIB + NNLOJET predictions into the $({p}_{{\rm{T}}}^{{\rm{\mu }}},{\eta }^{{\rm{\mu }}},{q}^{{\rm{\mu }}})$ observable, but the full impact of these fluctuations has not been rigorously assessed. The *m*_Z_ results obtained from the W-like fit with the MINNLO_PS_ predictions corrected to N^3+1^LL + N^3^LO and N^4+0^LL + N^3^LO are shifted down by about 3 MeV with respect to the nominal N^3+0^LL + NNLO result, respectively. Owing to the complexity of the calculation, we have assessed only the impact of the N^3^LO prediction for W^−^ production. The value of ${m}_{{{\rm{W}}}^{-}}$ is extracted from a fit to the $({p}_{{\rm{T}}}^{{\rm{\mu }}},{\eta }^{{\rm{\mu }}},{q}^{{\rm{\mu }}})$ distribution with only negatively charged muons selected. For the nominal configuration using SCETLIB + DYTURBO at N^3+0^LL + NNLO, the total uncertainty in ${m}_{{{\rm{W}}}^{-}}$ is 19.4 MeV, with a ${p}_{{\rm{T}}}^{{\rm{W}}}$ modelling uncertainty of 3.1 MeV. The ${m}_{{{\rm{W}}}^{-}}$ results at N^3+1^LL + N^3^LO and N^4+0^LL + N^3^LO differ from the N^3+0^LL + NNLO result by less than the ${p}_{{\rm{T}}}^{{\rm{W}}}$ modelling uncertainty. These checks show that our results are not significantly affected by HO predictions for Z and W boson production.

As an additional test of the measurement dependence on the accuracy of the ${p}_{{\rm{T}}}^{{\rm{V}}}$ modelling, we have performed the W-like *m*_Z_ measurement with the MINNLO_PS_ MC sample reweighted such that the predicted ${p}_{{\rm{T}}}^{{\rm{Z}}}$ matches the measured ${p}_{{\rm{T}}}^{\mathrm{\mu \mu }}$ distribution. The corrections are derived from the unfolding measurement shown in Fig. 2 and Supplementary Fig. 3 and applied directly to the MINNLO_PS_ prediction. The value of *m*_Z_ extracted from this configuration differs by −1.8 MeV with respect to the nominal result, to be compared with the 1.7 MeV uncertainty due to the ${p}_{{\rm{T}}}^{{\rm{Z}}}$ modelling. The stability of these results, and their consistency with the independently measured value of *m*_Z_, supports the use of the SCETLIB + DYTURBO prediction and its uncertainties. We also tested the impact of applying the same corrections, derived from the ratio of the unfolded data and the MINNLO_PS_ predictions for ${p}_{{\rm{T}}}^{{\rm{Z}}}$, to the W boson simulation. This procedure corresponds approximately to tuning the predictions to reproduce the ${p}_{{\rm{T}}}^{{\rm{Z}}}$ spectrum, under the assumption that the differences between the data and the ${p}_{{\rm{T}}}^{{\rm{Z}}}$ and ${p}_{{\rm{T}}}^{{\rm{W}}}$ predictions arise from the same sources. Given the weakness of this assumption, we do not consider this procedure to be an acceptable approach for the nominal result. The resulting shift in *m*_W_ with respect to the nominal result (based on the SCETLIB + DYTURBO prediction) is smaller than 0.5 MeV and the change in the total uncertainty is negligible.

Finally, we test a simultaneous fit to the $({p}_{{\rm{T}}}^{{\rm{\mu }}},{\eta }^{{\rm{\mu }}},{q}^{{\rm{\mu }}})$ distribution in W → μν events and the $({p}_{{\rm{T}}}^{{\rm{\mu \mu }}},{y}^{\mathrm{\mu \mu }})$ distribution in Z → μμ events. The TNPs are correlated across the W and Z boson processes, whereas uncertainties in the matching contributions and angular coefficients are left uncorrelated between the different processes. For the nonperturbative model, the Gaussian smearing parameters are considered independent for W and Z, whereas the CS anomalous rapidity is correlated. The *m*_W_ value extracted in this fit is shifted by +0.6 MeV compared with the nominal result. The total uncertainty is moderately reduced because of additional constraints on theory and experimental uncertainties that are correlated across the W and Z processes. Supplementary Fig. 18 presents a summary of these results, shown as a comparison to the nominal result and its uncertainty.

**Extended Data Fig. 7:**
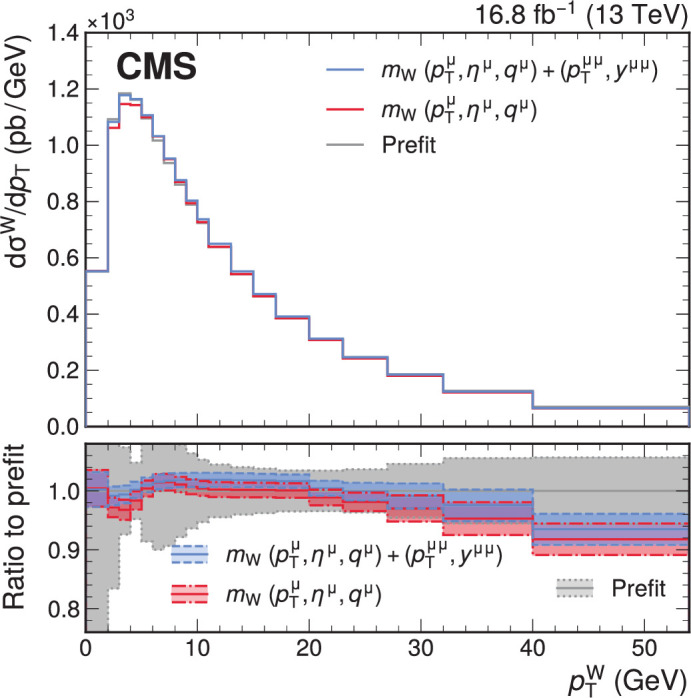
The generator-level ${{\boldsymbol{p}}}_{{\bf{T}}}^{{\bf{W}}}$ distribution, with three instances of the prediction and their uncertainty: before the maximum likelihood fit (“prefit”), and reflecting the results of the two fits described in the text. The distribution and uncertainties obtained from the combined $({p}_{{\rm{T}}}^{{\rm{\mu }}},{\eta }^{{\rm{\mu }}},{q}^{{\rm{\mu }}})$ and $({p}_{{\rm{T}}}^{{\rm{\mu \mu }}},{y}^{{\rm{\mu }}{\rm{\mu }}})$ fit is shown in red, whereas the blue band shows the distribution obtained from the nominal $({p}_{{\rm{T}}}^{{\rm{\mu }}},{\eta }^{{\rm{\mu }}},{q}^{{\rm{\mu }}})$ fit. The generator-level distribution predicted by SCETLIB + DYTURBO before incorporating in situ constraints is shown in gray. The ratio of the postfit predictions to the prefit prediction (in gray), as well as their uncertainties, are shown by the shaded bands in the lower panel.

The impact of including the ${p}_{{\rm{T}}}^{\mathrm{\mu \mu }}$ data in the fit is shown in Extended Data Fig. 7, which compares the generator-level ${p}_{{\rm{T}}}^{{\rm{W}}}$ spectrum modified by the best-fit values of nuisance parameters for the two fits. The consistency of the two results supports the conclusion that the ${p}_{{\rm{T}}}^{\mathrm{\mu \mu }}$ measurement is not required as an input to describe the ${p}_{{\rm{T}}}^{{\rm{W}}}$ distribution, with the added benefit of a reduced model dependence of the result. The loose assumptions about the correlation of the nonperturbative parameters between W and Z boson production limit the impact of including the ${p}_{{\rm{T}}}^{\mathrm{\mu \mu }}$ data.

### Helicity fit

Although the theoretical model and uncertainties described in section ‘Theoretical corrections and uncertainties’ reflect our best knowledge of QCD and of the proton structure, approximations of this model or the presence of new physics motivates the extraction of *m*_W_ using a parallel approach with a reduced model dependence, which we call ‘helicity fit’. With this technique we extract, from a likelihood fit to the $({p}_{{\rm{T}}}^{{\rm{\mu }}},{\eta }^{{\rm{\mu }}},{q}^{{\rm{\mu }}})$ distribution, not only the mass of the W boson but also, simultaneously, its polarization and the ${p}_{{\rm{T}}}^{{\rm{W}}}$ and *y*^W^ spectra. At the core of this alternative analysis is the observation that *m*_W_ variations induce a uniform scaling of the ${p}_{{\rm{T}}}^{{\rm{\mu }}}$ spectrum, whereas changes in the W boson polarization or in the $({p}_{{\rm{T}}}^{{\rm{W}}},{y}^{{\rm{W}}})$ double-differential cross sections lead to a nonuniform sculpting of the ${p}_{{\rm{T}}}^{{\rm{\mu }}}$ and *η*^μ^ spectra. We implement variations in the W boson polarization and in the $({p}_{{\rm{T}}}^{{\rm{W}}},{y}^{{\rm{W}}})$ distribution as a set of independent nuisance parameters in the signal likelihood function that is used to fit the measured $({p}_{{\rm{T}}}^{{\rm{\mu }}},{\eta }^{{\rm{\mu }}},{q}^{{\rm{\mu }}})$ distribution. The W boson polarization enters into our analysis procedure through the helicity decomposition of equation (3). We use helicity cross-sections, *σ*_*i*_, which correspond to the product of the angular coefficients *A*_*i*_ and the unpolarized cross section, and we neglect the terms with *i* > 4, predicted to be zero in first approximation and having no effect on our measurement (given that we integrate over the ϕ^*^ angle in equation (3)).

For each muon charge, the analysis covers the ${p}_{{\rm{T}}}^{{\rm{V}}}$ and *y*^V^ plane with nuisance parameters that represent variations of the production cross-section, separately for 7 bins in *y*^V^ (within ∣*y*^V^∣ < 3) times 8 bins in ${p}_{{\rm{T}}}^{{\rm{V}}}$ (for ${p}_{{\rm{T}}}^{{\rm{V}}} < 60\,\mathrm{GeV}$), plus 16 overflow bins. The unpolarized cross-section (*σ*^U+L^) and five helicity cross-sections are defined for each of those 144 bins, leading to a total of 864 nuisance parameters. The helicity cross-sections, *σ*_*i*_ ∝ *σ*^U+L^*A*_*i*_, are defined for *i* = 0–4 in terms of *A*_*i*_ and *σ*^U+L^ from equation (3). We propagate variations in the helicity amplitudes, which depend on the unobserved ${p}_{{\rm{T}}}^{{\rm{W}}}$ and *y*^W^, into a multitude of $({p}_{{\rm{T}}}^{{\rm{\mu }}},{\eta }^{{\rm{\mu }}},{q}^{{\rm{\mu }}})$ distributions, obtained by reweighting the simulated events. For each individual variation, the sum of all contributions is recomputed to get a new prediction for the $({p}_{{\rm{T}}}^{{\rm{\mu }}},{\eta }^{{\rm{\mu }}},{q}^{{\rm{\mu }}})$ distribution. The nuisance parameters are constrained around the theoretical predictions with uncertainties that are relaxed with respect to their theoretical values, used for the nominal result. The *σ*^U+L^ and *σ*_4_ parameters have very loose initial constraints, of ±50% and ±100% of the predicted cross-sections, respectively. The initial uncertainties in the four other helicity terms, for which the fit has limited constraining power, are defined by the spread of theory predictions (reflecting missing higher orders) and by uncertainties covering several different PDF sets. To ensure coverage of all possible correlated variations allowed by the theory model used in the baseline analysis, apart from the explicit helicity cross-section variations, we also retain the PDF and missing HO uncertainties, as well as the primordial-*k*_T_ smearing and nonperturbative uncertainties in the angular coefficients. The latter two are also retained in the unpolarized term, given that their impact on the cross-section at low ${p}_{{\rm{T}}}^{{\rm{V}}}$ is significant within the finite-width bins of the helicity cross-section variations. By contrast, we do not consider uncertainties in the unpolarized cross-section from resummation, matching and missing higher orders because they are largely redundant with respect to the explicit *σ*^U+L^ variations. This approach results in a significant reduction in model-dependent assumptions with respect to the nominal analysis.

We validate the helicity fit approach by measuring a negligible *m*_W_ bias in pseudo-data samples generated with different PDF sets and ${p}_{{\rm{T}}}^{{\rm{W}}}$ or *y*^W^ spectra, and by measuring the Z boson mass in the W-like configuration. The expected *m*_W_ uncertainty is evaluated for different prefit constraints on the helicity nuisance parameters. We observe only a mild dependence of the *m*_W_ uncertainty on all helicity terms, except for *σ*_3_, whose variations have a similar impact on ${p}_{{\rm{T}}}^{{\rm{\mu }}}$ as those resulting from *m*_W_ variations. Therefore, in the *m*_W_ extractions made to verify the stability of the measurement, we scale the prefit uncertainties of *σ*_3_ and of all the other *σ*_*i*_ terms by two independent factors.

**Extended Data Fig. 8:**
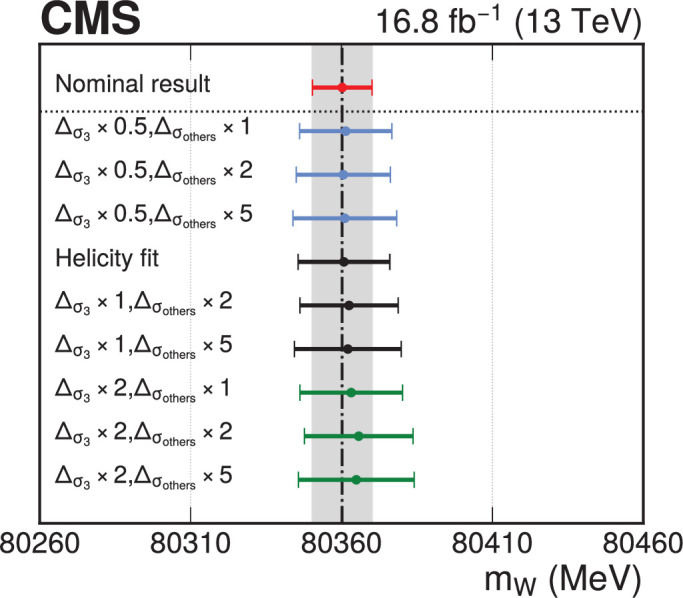
The W boson mass measured with the helicity fit for different scaling scenarios of the prefit helicity cross section uncertainties, denoted by ${{\boldsymbol{\Delta }}}_{{{\boldsymbol{\sigma }}}_{{\bf{3}}}}$ and ${{\boldsymbol{\Delta }}}_{{{\boldsymbol{\sigma }}}_{{\bf{others}}}}$ for the *σ*_3_ and the other components, respectively. The points are grouped and colored according to the scaling of *σ*_3_. The black line indicates the nominal result, with its uncertainties shown by the gray band.

Extended Data Fig. 8 shows the *m*_W_ values measured with the helicity fit for different scenarios of the prefit helicity cross-section uncertainties. We halved or doubled the default *σ*_3_ prefit uncertainty, to study possible shifts of the central value under more aggressive or conservative theoretical assumptions. For each of those scenarios, we inflated the other helicity cross-section uncertainties by factors of 2 or 5 (apart from the nominal uncertainty). All eight extra cases give central *m*_W_ values that are stable and consistent with both the baseline and helicity fit nominal results. Supplementary Fig. 6 shows the W boson differential cross-sections in ${p}_{{\rm{T}}}^{{\rm{W}}}$ and |*y*^W^|, extracted from the $({p}_{{\rm{T}}}^{{\rm{\mu }}},{\eta }^{{\rm{\mu }}},{q}^{{\rm{\mu }}})$ distributions through the decomposition of the helicity amplitudes in ${p}_{{\rm{T}}}^{{\rm{W}}}$ and *y*^W^ bins.

### The W-like Z and W boson mass measurements

**Extended Data Fig. 9:**
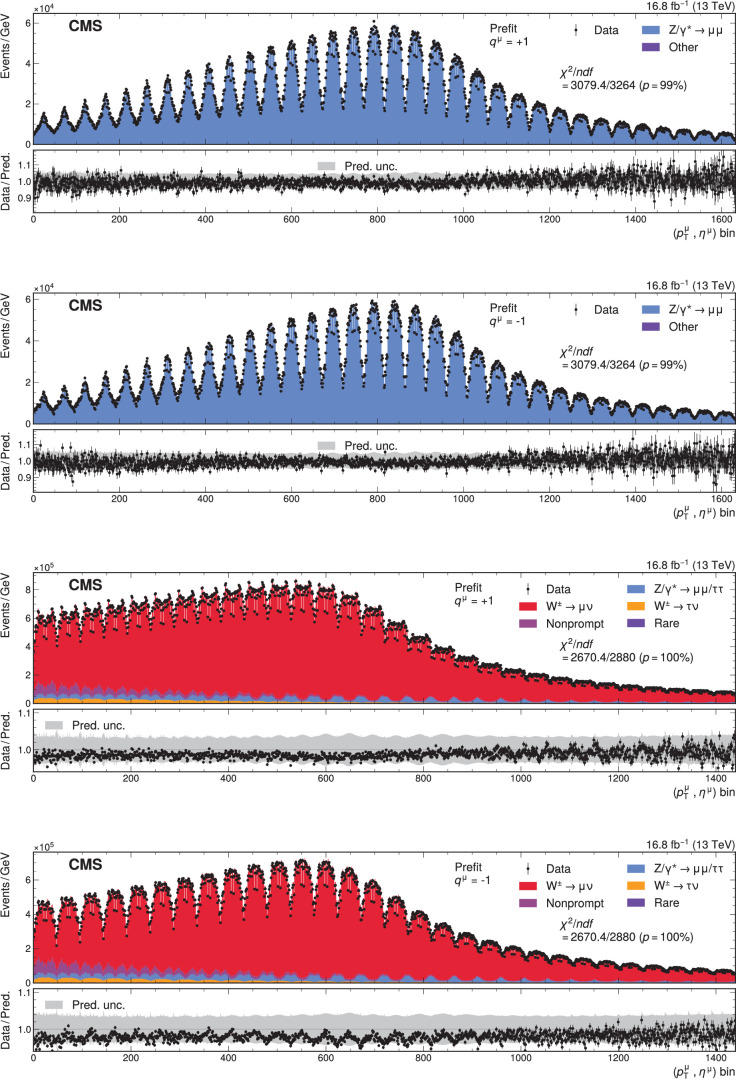
Measured and predicted $({{\boldsymbol{p}}}_{{\bf{T}}}^{{\boldsymbol{\mu }}},{{\boldsymbol{\eta }}}^{{\boldsymbol{\mu }}})$ distributions used in the W-like *m*_Z_ (upper two) and *m*_W_ (lower two) measurements for positively (upper and second from bottom) and negatively (second from top and lower) charged muons. The two-dimensional distribution is “unrolled” such that each bin on the *x*-axis represents one $({p}_{{\rm{T}}}^{{\rm{\mu }}},{\eta }^{{\rm{\mu }}})$ cell. The gray band represents the uncertainty in the prediction, before the fit to the data. The bottom panel shows the ratio of the number of events observed in data to the nominal prediction. The vertical bars represent the statistical uncertainties in the data.

**Extended Data Fig. 10:**
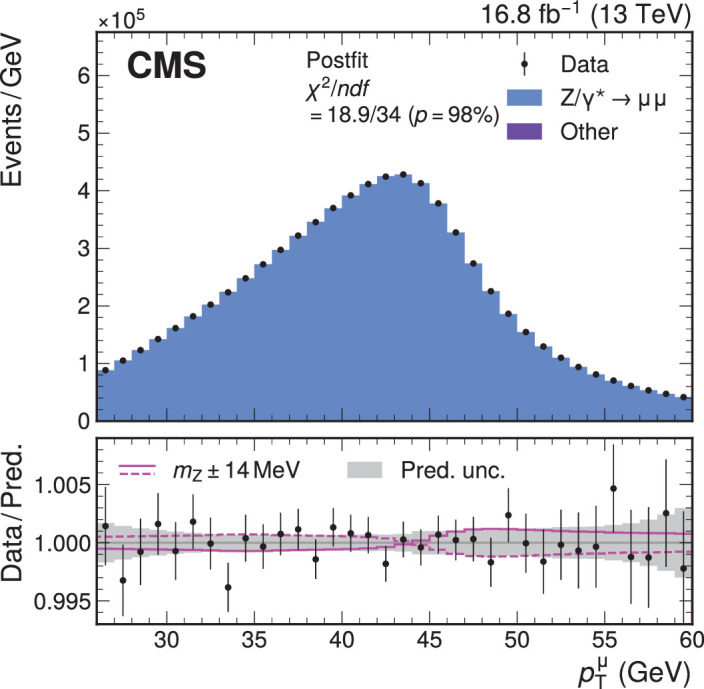
Measured and simulated ${{\boldsymbol{p}}}_{{\bf{T}}}^{{\boldsymbol{\mu }}}$ distributions, with the prediction adjusted according to the best fit values of nuisance parameters and of *m*_Z_ obtained from the maximum likelihood fit of the W-like *m*_Z_ analysis. The vertical bars represent the statistical uncertainties in the data. The bottom panel shows the ratio of the number of events observed in data to the nominal prediction. The solid and dashed purple lines represent, respectively, the relative impact of an increase and decrease of *m*_Z_ by 14 MeV. The uncertainties in the predictions, after the systematic uncertainty profiling in the maximum likelihood fit, are shown by the shaded band.

Extended Data Fig. 9 and Supplementary Fig. 8 show the $({p}_{{\rm{T}}}^{{\rm{\mu }}},{\eta }^{{\rm{\mu }}})$ distributions used in the binned maximum likelihood template fits to perform the W-like *m*_Z_ and *m*_W_ measurements, before and after the maximum likelihood fit, respectively. The *η*^μ^ binning allows sensitivity to discontinuities in the geometry of the detectors and maximally exploits in situ constraints of systematic uncertainties. The ${p}_{{\rm{T}}}^{{\rm{\mu }}}$ binning roughly corresponds to the ${p}_{{\rm{T}}}^{{\rm{\mu }}}$ resolution, useful to enhance the sensitivity to the measured mass, while avoiding fluctuations in the simulated templates that could potentially lead to undercoverage of the estimated uncertainties^102^. The predicted and observed ${p}_{{\rm{T}}}^{{\rm{\mu }}}$ distribution of the W-like analysis, with the prediction corrected by the best-fit values of the nuisance parameters after the maximum likelihood fit to the $({p}_{{\rm{T}}}^{{\rm{\mu }}},{\eta }^{{\rm{\mu }}},{q}^{{\rm{\mu }}})$ distribution, is shown in Extended Data Fig. 10. The impact of a variation of *m*_Z_ corresponding to the total uncertainty of the measurement is also shown, as well as the uncertainties in the prediction, illustrating the precision of the measurement.

The uncertainty due to the size of the simulated samples is estimated via the Barlow–Beeston approach^103^, as simplified by Conway^53^. The estimate is increased by 25% to account for the effect of fluctuations in the alternate templates used to construct the systematic variations^102^. The 1.25 scaling factor is estimated by evaluating the coverage of the uncertainty with bootstrap resampling of the simulated samples^104^. The width of the W boson, *Γ*_W_, is varied with the mass according to the SM relationship ${\varGamma }_{{\rm{W}}}\propto {m}_{{\rm{W}}}^{3}$. The theoretical uncertainty of 0.6 MeV from the EW fit is taken as an uncertainty in *Γ*_W_, but we verified that using the experimental uncertainty in ref. ^1^ has a negligible impact on the results. The uncertainty in *m*_W_ from *Γ*_W_ is <0.2 MeV. Systematic uncertainties whose +1*σ* and −1*σ* variation templates affect the event yields asymmetrically are decomposed into two symmetric variations, defined such that the symmetric and anti-symmetric components are represented separately. This procedure preserves the total variance and covariance of the event yields and reduces non-linearities in the likelihood, simplifying the evaluation of uncertainties and impacts.

Several additional tests are performed to verify the robustness of the statistical procedure. The likelihood minimization is performed with an independent implementation of the likelihood function and with a different minimization algorithm, which yields a value of *m*_W_ that is identical to the nominal result within the numerical precision. The minimization was performed 10 times with the starting values of *m*_W_ and the nuisance parameters set to random values. The resulting minima of the likelihood function and the associated *m*_W_ values are consistent with the nominal result in all cases. Finally, an estimation of *m*_W_ is performed linearizing the likelihood function and absorbing all systematic uncertainties into the data covariance matrix, such that the systematic uncertainties are not explicit fit parameters. Instead, they are treated equivalently to the statistical uncertainty of the data. In this configuration, the minimum can be evaluated analytically, without the need for iterative minimization. As discussed in ref. ^105^, this treatment is mathematically equivalent when the likelihood is purely quadratic. The value of *m*_W_ extracted with this procedure is shifted by 1.5 MeV with respect to the nominal result. The difference is primarily due to the linearization of uncertainties associated with the nonprompt background estimate, which in this approximated treatment are not scaled with the yield of the nonprompt background in case it differs from its prefit value. Although the nominal treatment is a more accurate representation of the uncertainties, the stability of the result within the associated uncertainties addresses potential concerns about the robustness of the likelihood minimization^106^.

The individual systematic uncertainties in the W-like *m*_Z_ and *m*_W_ measurements are presented in Extended Data Table 2. The uncertainty breakdown labelled as ‘Nominal impact’ is computed according to the procedure described in ref. ^33^, in which the data statistical uncertainty corresponds to a hypothetical analysis with no systematic uncertainties. The impact for all other sources of uncertainty corresponds to the amount by which the total uncertainty would decrease in quadrature if that source were removed from the analysis. The total uncertainty cannot be calculated as the sum in quadrature of the impacts because of correlations between the partial uncertainties.

This uncertainty breakdown is not directly comparable to that of ATLAS^19^, which uses an alternative method to define the uncertainty contributions, referred to as global impacts^105^. In that approach, the data statistical uncertainty is, instead, computed with the nuisance parameters present in the fit. If the data constrain the nuisance parameters in situ, beyond the externally imposed constraints, then fluctuations in the data and the simulated event samples become correlated with the fitted values of the nuisance parameters, which, in turn, increases the statistical components of the uncertainty. The impacts of systematic sources are computed considering fluctuations of the corresponding external measurements (that is, of the so-called global observables) within their uncertainties. In the presence of stronger in situ constraints, this method typically leads to smaller impacts than our approach. These two procedures differ only in the split between the statistical and systematic components of the uncertainty; they do not affect the total uncertainty of the result. To facilitate the comparison with the uncertainty breakdown of the ATLAS measurement, Extended Data Table 2 also reports the leading uncertainties using global impacts.

Supplementary Table 2 shows a summary of the number of nuisance parameters included in the likelihood for the W-like *m*_Z_ and *m*_W_ fits. The parameters are categorized into groups, corresponding to the main sources of uncertainty reported in Extended Data Table 2, and gathering conceptually related systematic uncertainties. Uncertainties specific to W bosons, for instance, the mass or width variations, are not implemented in the W-like *m*_Z_ analysis because the W + jets background is negligible.

### Measurement of ${{\boldsymbol{m}}}_{{{\bf{W}}}^{+}}-{{\boldsymbol{m}}}_{{{\bf{W}}}^{-}}$

Our measurement assumes that the W^+^ and W^−^ bosons have identical masses, ${m}_{{{\rm{W}}}^{+}}={m}_{{{\rm{W}}}^{-}}$, as required by charge, parity and time reversal symmetry. This requirement reduces the impact of uncertainties that affect the two charges differently, including the PDFs, angular coefficients and the alignment terms of the muon momentum calibration. By relaxing this requirement, we perform a measurement of the mass difference,

$${m}_{{{\rm{W}}}^{+}}-{m}_{{{\rm{W}}}^{-}}=57.0\pm 30.3\,{\rm{MeV}}.$$

The significant increase in the uncertainty compared with the *m*_W_ measurement is due to uncertainties that have a strong negative correlation between the two charges. In particular, the muon momentum calibration contributes an uncertainty of 22.0 MeV, the angular coefficients contribute 18.7 MeV and the PDF uncertainty is 11.8 MeV. The statistical uncertainty of the data is 4.7 MeV. The *P*-value indicating the compatibility of this result and the expectation of ${m}_{{{\rm{W}}}^{+}}-{m}_{{{\rm{W}}}^{-}}=0$ is 6.0%, or about 1.9*σ*. The charge-independent *m*_W_ value measured in this configuration is shifted by 0.3 MeV with respect to the nominal result, having a negligible effect on the total uncertainty. The correlation coefficient between ${m}_{{{\rm{W}}}^{+}}$ and ${m}_{{{\rm{W}}}^{-}}$ is *ρ*_+−_ = − 0.40, whereas the correlation between the mass difference and mass average is only 0.02. The small correlation between *m*_W_ and ${m}_{{{\rm{W}}}^{+}}-{m}_{{{\rm{W}}}^{-}}$ is a consequence of a strong degree of anticorrelation for the alignment components of the ${p}_{{\rm{T}}}^{{\rm{\mu }}}$ calibration uncertainties, and the uncertainties in the *A*_3_ angular coefficient.

If the ${m}_{{{\rm{W}}}^{+}}$ and ${m}_{{{\rm{W}}}^{-}}$ values are measured independently, the total uncertainty in each is about 20 MeV, with statistical uncertainties of 2 MeV and 3 MeV, respectively. Because the statistical uncertainties are negligible, the difference between the W^+^ and W^−^ production rates does not have a visible effect on the charge-inclusive *m*_W_ measurement, which is the average of the two results. The total variance in the sum (${m}_{{{\rm{W}}}^{+}}+{m}_{{{\rm{W}}}^{-}}=2{m}_{{\rm{W}}}$) or difference (${m}_{{{\rm{W}}}^{+}}-{m}_{{{\rm{W}}}^{-}}$) is ${\sigma }_{+}^{2}+{\sigma }_{-}^{2}+2c\,{\rho }_{+-}{\sigma }_{+}{\sigma }_{-}$, where ${\sigma }_{+}^{2}$ and ${\sigma }_{-}^{2}$ are the variances of ${m}_{{{\rm{W}}}^{+}}$ and ${m}_{{{\rm{W}}}^{-}}$, respectively, and *c* = +1 for the sum and −1 for the difference. The opposite effect of *ρ*_+−_ on the propagation of uncertainty results in a factor of around 1/3 between the *m*_W_ uncertainty and that of ${m}_{{{\rm{W}}}^{+}}-{m}_{{{\rm{W}}}^{-}}$.

As a validation of this result, we also perform the corresponding measurement in the case of the W-like *m*_Z_ measurement using the positively and negatively charged muons. In this case, the two leptons are from the same object and, therefore, the comparison is purely a validation of the theoretical and experimental inputs. The result when selecting positively charged muons in odd event-number events is

$${m}_{{{\rm{Z}}}^{+}}-{m}_{{{\rm{Z}}}^{-}}=30.9\pm 32.5\,{\rm{MeV}},$$

and for the reversed muon charge and event-number selection, we get

$${m}_{{{\rm{Z}}}^{+}}-{m}_{{{\rm{Z}}}^{-}}=6.4\pm 32.3\,{\rm{MeV}}.$$

Apart from the PDFs, which are not relevant for this measurement, the breakdown of uncertainties is similar to the ${m}_{{{\rm{W}}}^{+}}-{m}_{{{\rm{W}}}^{-}}$ case. The muon momentum scale and the angular coefficients contribute uncertainties of 23.1 MeV and 14.5 MeV, respectively. The statistical uncertainty of the data is 13.9 MeV.

Supplementary Table 3 shows the impacts on the difference between the measured mass with positive or negative muons in the W-like *m*_Z_ and *m*_W_ analyses, comparing with the nominal result from the simultaneous fit to both charges, and using nominal impacts. The breakdown of uncertainties from the global definition of the impacts is also reported, for completeness.

We have performed several additional checks that confirm that the small tension with the expectation of ${m}_{{{\rm{W}}}^{+}}={m}_{{{\rm{W}}}^{-}}$ does not reflect a bias or an underestimation of our uncertainties that would impact our result. The alignment components of the muon momentum scale calibration and the *A*_3_ angular coefficient uncertainties are the dominant sources affecting the ${m}_{{{\rm{W}}}^{+}}-{m}_{{{\rm{W}}}^{-}}$ measurement. Therefore, we repeat both measurements after varying the central value of these parameters by 1*σ* such that the charge difference is reduced, keeping their relative uncertainty fixed. Up variations of the ${p}_{{\rm{T}}}^{{\rm{\mu }}}$ scale alignment terms, and down variations of the *A*_3_ coefficient uncertainties, each reduce ${m}_{{{\rm{W}}}^{+}}-{m}_{{{\rm{W}}}^{-}}$. The maximum shift in ${m}_{{{\rm{W}}}^{+}}-{m}_{{{\rm{W}}}^{-}}$ when varying these two terms, either independently or coherently, moves the result towards zero by 1.2*σ*, compared with the ${m}_{{{\rm{W}}}^{+}}-{m}_{{{\rm{W}}}^{-}}$ measurement with the nominal uncertainty model. If the alignment parameters of the muon momentum scale calibration are extracted from Z → μμ events, the measured ${m}_{{{\rm{W}}}^{+}}-{m}_{{{\rm{W}}}^{-}}$ shifts towards zero by 16 MeV, with a total uncertainty of 25 MeV. In the extreme configuration in which the alignment term is varied by 3*σ*, resulting in ${m}_{{{\rm{W}}}^{+}}-{m}_{{{\rm{W}}}^{-}}\approx 0$, the extracted *m*_W_ differs from our nominal result by 0.6 MeV.

### Results with alternative parton distribution functions

We performed the *m*_W_ measurement using alternative PDF sets, with and without scaling factors, following the procedure described in section ‘Parton distribution functions’. The results are shown in Extended Data Table 3 and Supplementary Fig. 7. The scaling procedure, combined with the uncertainty profiling and in situ data constraints, improves the consistency between the *m*_W_ results obtained with the different PDF sets. If no uncertainty scaling is used, the results vary by 8.1 MeV, from 80 355.1 ± 9.3 MeV (NNPDF4.0) to 80 363.2 ± 9.9 MeV (PDF4LHC21). If we use the uncertainty scaling, the spread of results is reduced to 6.2 MeV, ranging from 80 357.0 ± 10.8 MeV (NNPDF4.0 with uncertainties scaled by 5.0) to 80 363.2 ± 9.9 MeV (PDF4LHC21 with no scaling factor). The spread of these values is within the total PDF uncertainty of our nominal measurement (performed with CT18Z).

We also tested the impact of excluding the PDF uncertainties from the profiling procedure in the maximum likelihood fit, and estimated their impact by repeating the fit for each PDF eigenvector variation. The total uncertainty is defined as the sum in quadrature of the PDF eigenvector variation uncertainties and the total profiled uncertainty. In this configuration, when using the CT18Z PDF set, the measured *m*_W_ value decreases by less than 2 MeV, within the PDF uncertainty of the nominal result using profiling. The PDF uncertainty estimated from the CT18Z set increases by a factor of about 2.5 when the PDF uncertainty is not profiled, and the total uncertainty in *m*_W_ increases by about 3 MeV. The shift in the measured *m*_W_ value is within 1*σ* of the profiled PDF uncertainty for NNPDF4.0, MSHT20, MSHT20aN3LO and PDF4LHC, and within 2*σ* for NNPDF3.1 and CT18. The PDF uncertainty increase varies between a factor of 1.5 (NNPDF4.0) and a factor of 3 (CT18). We have explicitly verified the coverage of the uncertainty, for both the profiled and the unprofiled cases, using pseudo-experiments whose pseudo-data are obtained from a random draw of counts as predicted from a random realization of the statistical model parameters. This method corresponds to the ‘frequentist toys’ approach described in ref. ^107^.

### Additional validation checks of experimental inputs

Several additional tests were performed to ensure that the analysis is robust with respect to variations in the selections used.

The *m*_Z_ extraction from Z → μμ events is performed in subsets of events defined by the relative location of the two muons in the CMS detector. Supplementary Fig. 9 shows that the nominal *m*_Z_ result is compatible with the results obtained when both muons are central (∣*η*^μ^∣ < 0.9), one is central and the other is forward, or both are forward. The same exercise is performed depending on *η*^μ^, by requiring both muons on the *η*^μ^ < 0 half of the detector, both on the positive half, and one in each half of the detector, with the same conclusions. Concerning the W-like *m*_Z_ and *m*_W_ analyses, the *m*_V_ extraction is performed in 24 bins of *η*^μ^. The results are shown in Supplementary Fig. 11, in which the compatibility with the nominal result can also be appreciated. The *η*^μ^ dependence of the result is also assessed by evaluating the difference between the *m*_V_ values measured when selecting muons in the central (|*η*| < 0.9) and forward (0.9 < |*η*| < 2.4) regions of the detector. The difference between the *m*_W_ values measured with central muons and with forward muons is 15.3 ± 14.7 MeV. For the W-like *m*_Z_ analysis, the corresponding result is 22.8 ± 21.1 MeV.

We test the stability of the measurement by performing the *m*_W_ extraction in separate data collection periods and by dividing the dataset into five regions according to the number of reconstructed vertices. The number of reconstructed vertices is strongly correlated with the number of pileup interactions, and the average pileup increased with time during data taking. Therefore, these checks confirm that the measurement is stable with respect to pileup, as well as other time-dependent changes in operational conditions. Because of the limited sample size, we do not derive independent efficiency SFs per period. Instead, we use the nominal SFs measured for the inclusive dataset. We estimate the impact of time-dependent effects that are not modelled by the simulation and apply them as uncertainties in the SFs per data collection period. An independent *m*_W_ parameter is assigned to each bin, and they are fitted simultaneously. Most of the experimental uncertainties are uncorrelated across the independent bins. The results are presented in Supplementary Figs. 13 and 14, which show good compatibility with the nominal measurement.

Moreover, we evaluate the effect of splitting the data by the muon azimuthal angle *ϕ*^μ^. For this study, we use the nominal SFs and apply uncertainties to account for the estimated variation of the efficiency SFs across the *ϕ*^μ^ regions. As shown in Supplementary Fig. 12, the results are consistent with the nominal measurement. We assess the impact of the ${m}_{{\rm{T}}}^{{\rm{W}}}$ selection in the *m*_W_ measurement by repeating the measurement with different ${m}_{{\rm{T}}}^{{\rm{W}}}$ thresholds, from 30 GeV to 50 GeV. The summary is shown in Supplementary Fig. 15. The largest deviations from the nominal value correspond to −2.8 MeV and 3.3 MeV, comparable to the total nonprompt-muon background uncertainty. The total uncertainty varies inversely with the ${m}_{{\rm{T}}}^{{\rm{W}}}$ threshold, from 10.1 MeV to 9.7 MeV, because of the reduced impact of the nonprompt-muon background uncertainty when the ${m}_{{\rm{T}}}^{{\rm{W}}}$ threshold is increased. However, the nonprompt-muon background estimation and the recoil calibration and uncertainties have not been independently optimized for the varied thresholds.

Other tests that were performed and also showed no incompatibility with the corresponding nominal result are as follows:

1. performing the W-like *m*_Z_ and *m*_W_ analyses splitting events by the sign of *η*^μ^, for each half of the CMS detector separately;
2. performing the *m*_W_ analysis reducing the ${p}_{{\rm{T}}}^{{\rm{\mu }}}$ range considered by removing 4 GeV on the high end, on the low end, and on both ends; and
3. performing the *m*_W_ analysis treating the normalization of the W signal process unconstrained. A scaling factor of 0.979 ± 0.026 is obtained, in agreement with the SM expectation of unity.

## Online content

Any methods, additional references, Nature Portfolio reporting summaries, source data, extended data, supplementary information, acknowledgements, peer review information; details of author contributions and competing interests; and statements of data and code availability are available at 10.1038/s41586-026-10168-5.

## Supplementary information

Supplementary Information This file contains Supplementary Figs. 1–18 and Supplementary Tables 1–3.

## Figures and Tables

**Extended Data Table 1:**
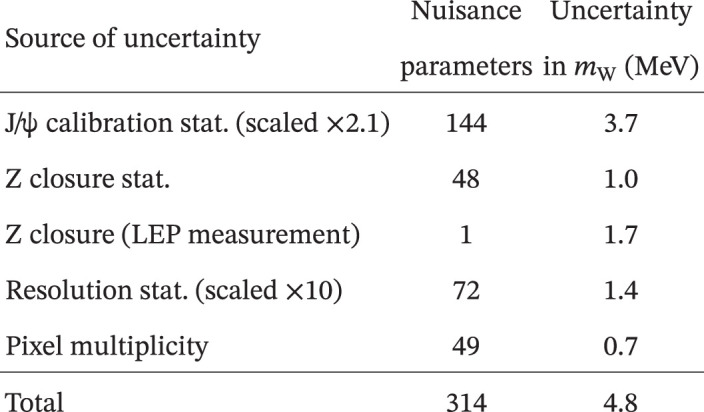
Breakdown of muon momentum calibration uncertainties

**Extended Data Table 2:**
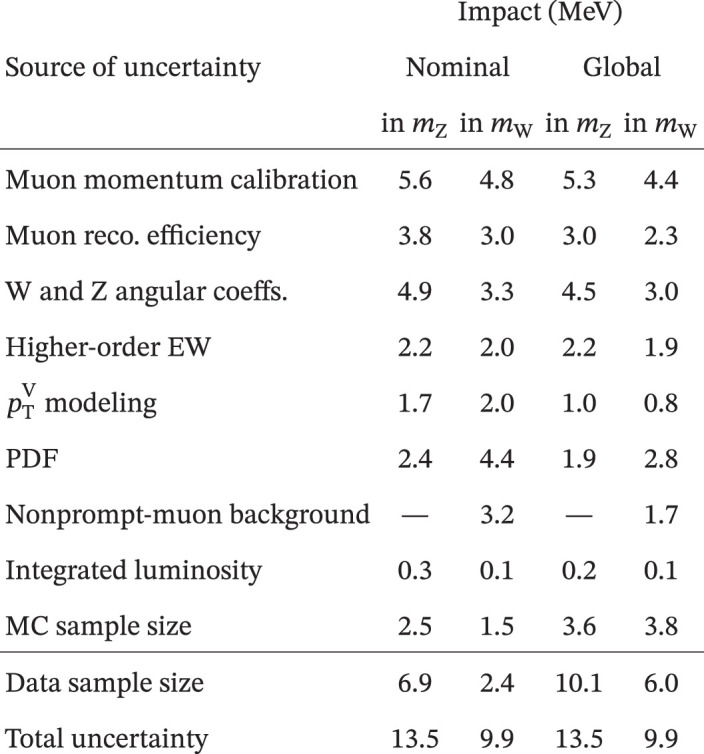
Uncertainties in the W-like *m*_Z_ and *m*_W_ measurements, with contributions to the total uncertainty from individual sources separated according to the “nominal”^33^ and “global”^105^ definitions of the impacts

**Extended Data Table 3:**
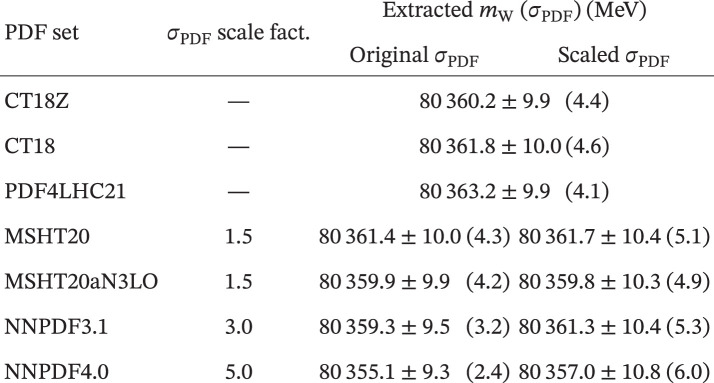
The *m*_W_ values measured for different PDF sets

The second column indicates the prefit uncertainty scaling factors required to cover the central predictions of the considered PDF sets, as described in the text. The observed values, with uncertainties scaled following the procedure described in Section A 8 and with the default unscaled uncertainties, are shown in the last two columns. The uncertainty in *m*_W_ from the PDF is indicated in parenthesis following the total uncertainty.

## Data Availability

Release and preservation of data used by the CMS Collaboration as the basis for publications is guided by the CMS data preservation, reuse and open access policy (https://opendata.cern.ch/record/415). Tabulated results are provided in the HEPData record for this work (10.17182/hepdata.155627).

## Code availability

The CMS core software is publicly available in our GitHub repository (github.com/cms-sw/cmssw). The data and simulation were processed with v.10_6_26 to produce output in the NanoAOD^108^ format. The processing of the data into histograms was performed with the WRemnants framework, which is publicly available in a GitHub repository (github.com/WMass/WRemnants). The version tagged ‘published’ has been used for the results in the paper. The statistical analysis was performed with the Rabbit software framework (GitHub repository; github.com/WMass/rabbit), which was extensively validated against the results of the CMS Combine^107^ statistical analysis tool.
