# Supplementary material for: High-precision measurement of the W boson mass with the CMS experiment
Source: Nature. 2026 Apr 8;652(8109):321–7. doi: 10.1038/s41586-026-10168-5 (PMC13061639; doi:10.1038/s41586-026-10168-5)
Supplement: Supplementary file 1 — This file contains Supplementary Figs. 1–18 and Supplementary Tables 1–3. [file 41586_2026_10168_MOESM1_ESM.pdf]

nature portfolio

https://doi.org/10.1038/s41586-026-10168-5

Supplementary information

High-precision measurement of the W boson mass with the CMS experiment

In the format provided by the authors and unedited

EUROPEAN ORGANIZATION FOR NUCLEAR RESEARCH (CERN)

CERN-EP-2024-308
2025/12/15

CMS-SMP-23-002

High-precision measurement of the W boson mass with the CMS experiment

—Supplemental information—
additional figures and tables

The CMS Collaboration

Submitted to Nature

© 2025 CERN for the benefit of the CMS Collaboration. CC-BY-4.0 license

1 Supplementary information: additional figures and tables

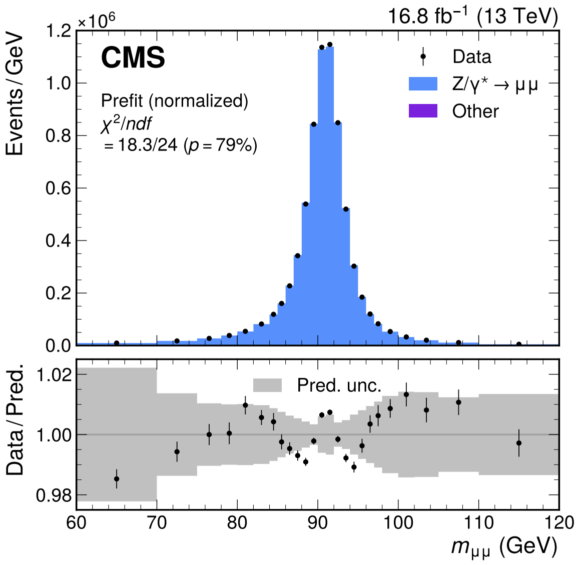

Figure 1: Measured and simulated $Z \rightarrow \mu\mu$ dimuon mass distributions, after applying the muon momentum scale and resolution corrections. The simulated predictions and uncertainties are scaled to match the number of observed data events. The vertical bars represent the statistical uncertainties in the data. The bottom panel shows the ratio of the number of events observed in data and of variations in the predictions to that of the total nominal prediction.

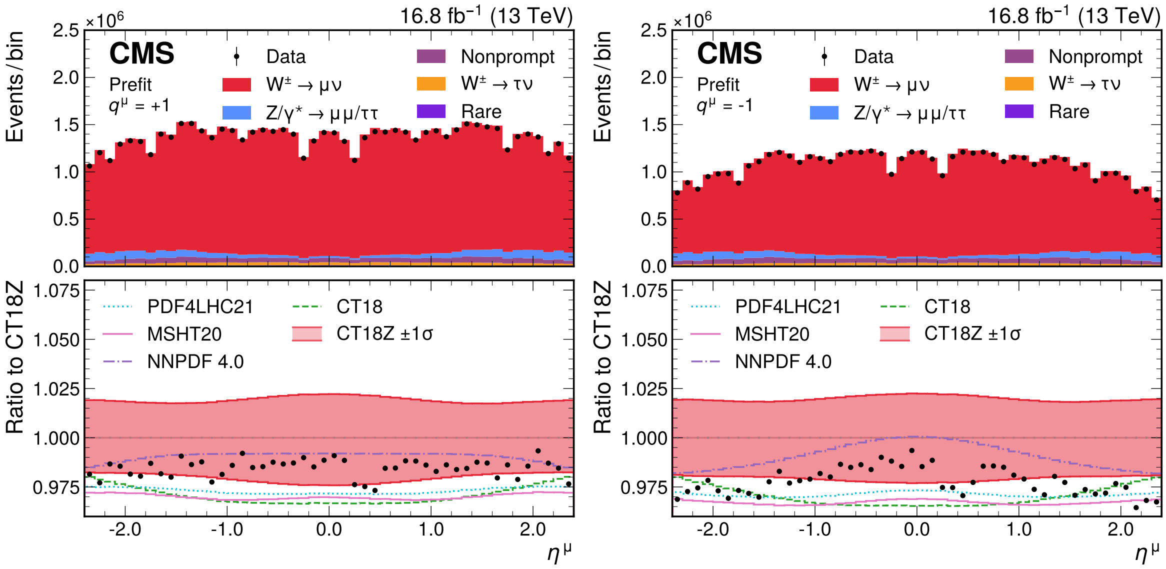

Figure 2: Measured and predicted η^{μ} distributions for positively (left) and negatively (right) charged muons. The nominal prediction, obtained with the CT18Z PDF set, is shown in filled red. The uncertainty, evaluated as the sum of the eigenvector variation sets, is represented by the filled band in the lower panel. The predictions using the PDF4LHC21, MSHT20, NNPDF4.0, and CT18 sets are also shown (without uncertainty bands). The vertical bars represent the statistical uncertainties in the data. The bottom panel shows the ratio of the number of events observed in data and of variations in the predictions to that of the nominal prediction.

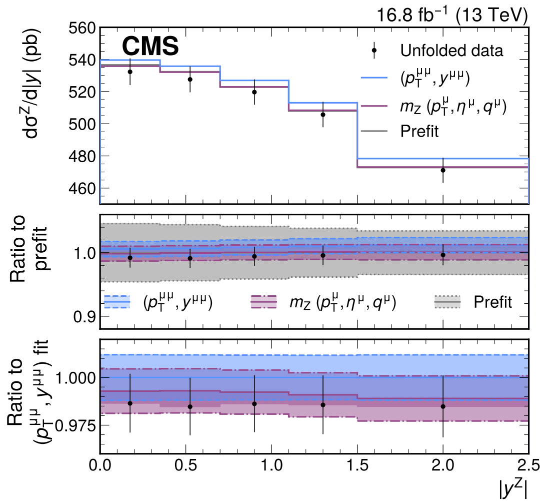

Figure 3: Unfolded measured $|y^Z|$ distribution (points) compared with the generator-level SCETLIB+MINNLO$_{PS}$ predictions before (prefit, gray) and after adjusting the nuisance parameters to the best fit values obtained from the W-like m_Z fit (magenta) or from the direct fit to the $p_T^{\mu\mu}$ distribution (blue). The results are obtained with the selection $|y^Z| < 2.5$ and $p_T^Z < 54\,\text{GeV}$. The center panel shows the ratio of the predictions and unfolded data to the prefit prediction. The uncertainty in the prefit prediction is shown by the shaded gray area. The bottom panel shows the ratio of the predictions and unfolded data to the postfit prediction from the fit to the $(p_T^{\mu\mu}, y^{\mu\mu})$ distribution. The postfit uncertainties in the predictions are shown in the shaded magenta and blue bands. The vertical bars represent the total uncertainty in the unfolded data.

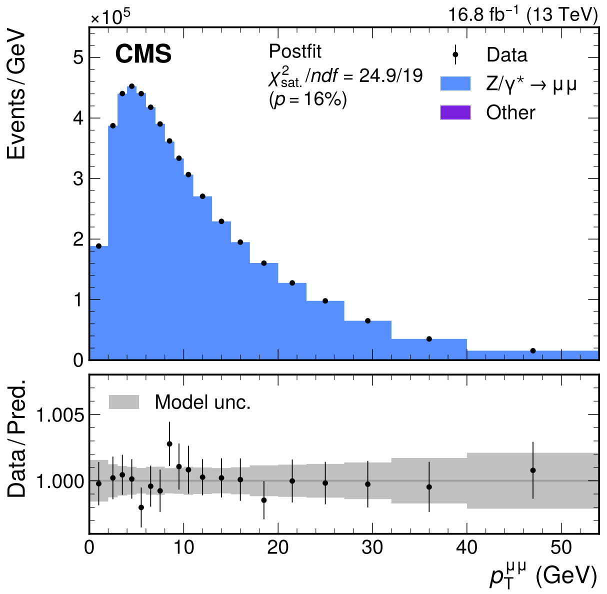

Figure 4: Measured and simulated $p_T^{\mu\mu}$ distributions in selected $Z \to \mu\mu$ events, with the normalization and uncertainties of the prediction set to the postfit values. The gray band represents the total systematic uncertainty. The vertical bars represent the statistical uncertainties in the data. The bottom panel shows the ratio between the number of events observed in data, including variations in the predictions, and the nominal prediction.

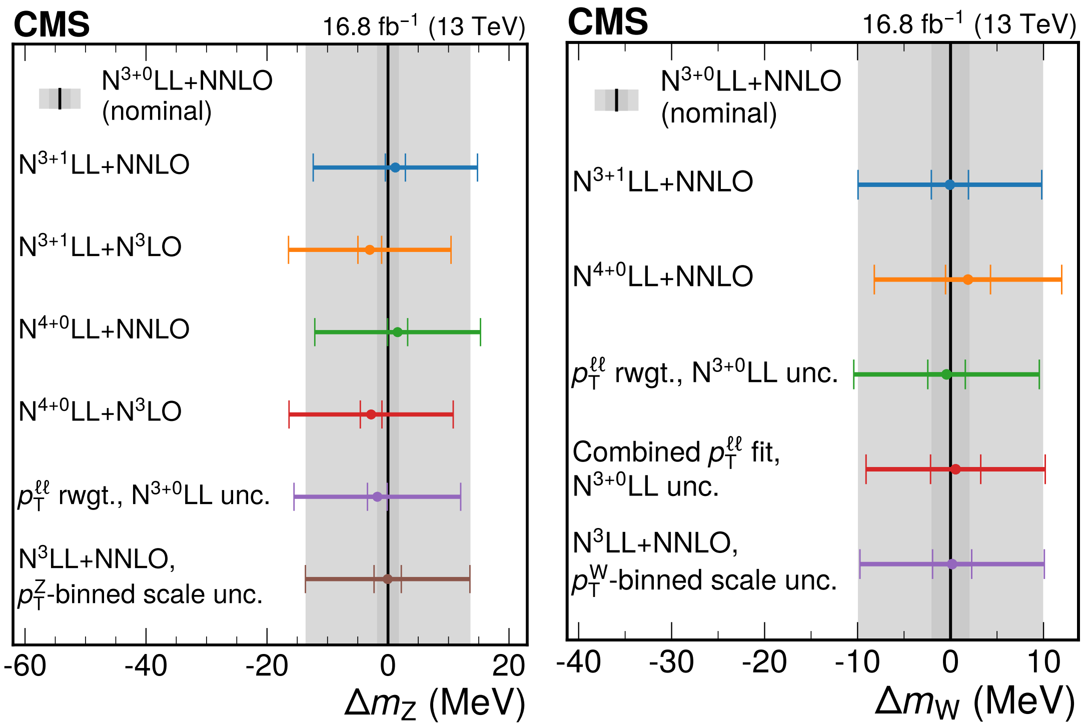

Figure 5: Comparison of the nominal result and its theory uncertainty for the W-like m_Z measurement (left) and the m_W measurement (right), using SCETLIB+DYTURBO at N^{3+0}LL+NNLO, with the difference in m_V measured when using alternative approaches to the p_T^V modeling and its uncertainty. The results from alternative approaches to the p_T^V modeling and uncertainty are shown as points. The solid black line represents the nominal result, the inner shaded gray band shows the p_T^V modeling uncertainty, and the outer shaded gray band shows the total uncertainty in the nominal result. The p_T^V modeling uncertainties are shown as the inner bars while the outer bars denote the total uncertainties.

Table 1: Goodness-of-fit test statistic for different PDF sets when fitting simultaneously the η^μ distributions for selected W^+ (W^-) events and the $y^{\mu\mu}$ distribution for $Z \to \mu\mu$ events. Both the saturated likelihood ratios, which are expected to follow a χ^2 distribution with ndf degrees of freedom if the model is an accurate representation of the data, and the associated p-value are shown. The fit is performed in the nominal configuration with all uncertainties (left column), nominal configuration without PDF and α_s uncertainties (middle column), and nominal configuration without theory uncertainties (right column).

PDF set	Nominal fit		Without PDF+α_s unc.		Without theory unc.	
	χ^2/ndf	p-val. (%)	χ^2/ndf	p-val. (%)	χ^2/ndf	p-val. (%)
CT18Z	100.7/116	84	125.3/116	26	103.8/116	78
CT18	100.7/116	84	153.2/116	1.0	105.7/116	74
PDF4LHC21	97.7/116	89	105.5/116	75	104.1/116	78
MSHT20	97.0/116	90	107.4/116	70	98.8/116	87
MSHT20aN3LO	99.0/116	87	122.8/116	31	101.9/116	82
NNPDF3.1	99.1/116	87	105.5/116	75	115.0/116	51
NNPDF4.0	99.7/116	86	104.3/116	77	116.7/116	46

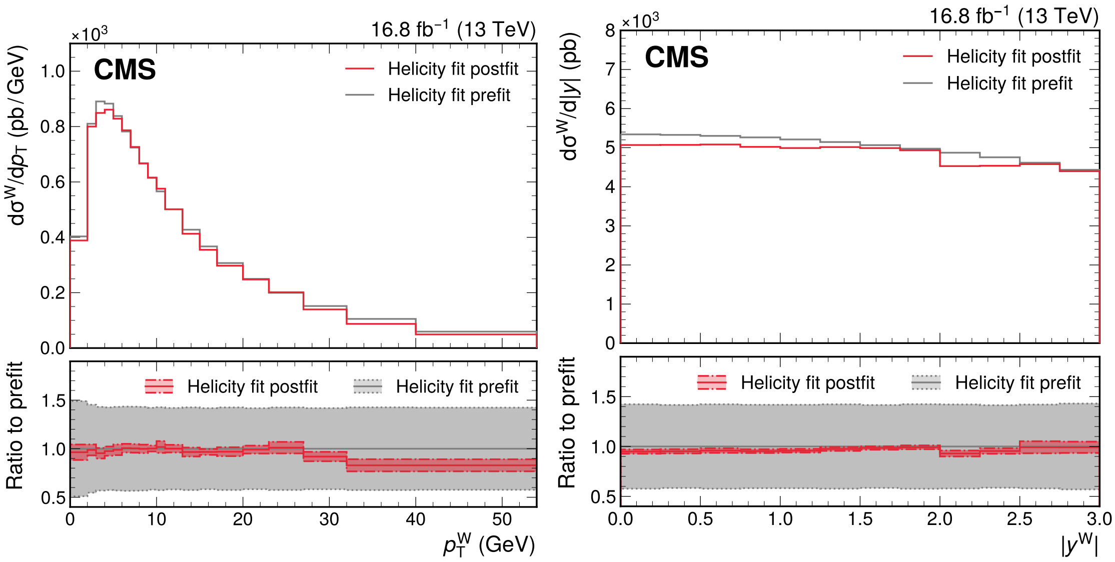

Figure 6: Differential W boson production cross section, in the W $\to \mu\nu$ decay channel, in p_T^W (left) and $|y^W|$ (right), measured from the $(p_T^\mu, \eta^\mu, q^\mu)$ distributions using the helicity fit approach (in red). The SCETLIB+DYTURBO generator-level predictions, before incorporating in situ constraints, are also shown (in gray). The results are shown for the selection $|y^W| < 3.0$ and $p_T^W < 54$ GeV. The lower panel shows the ratio between the postfit and prefit spectra.

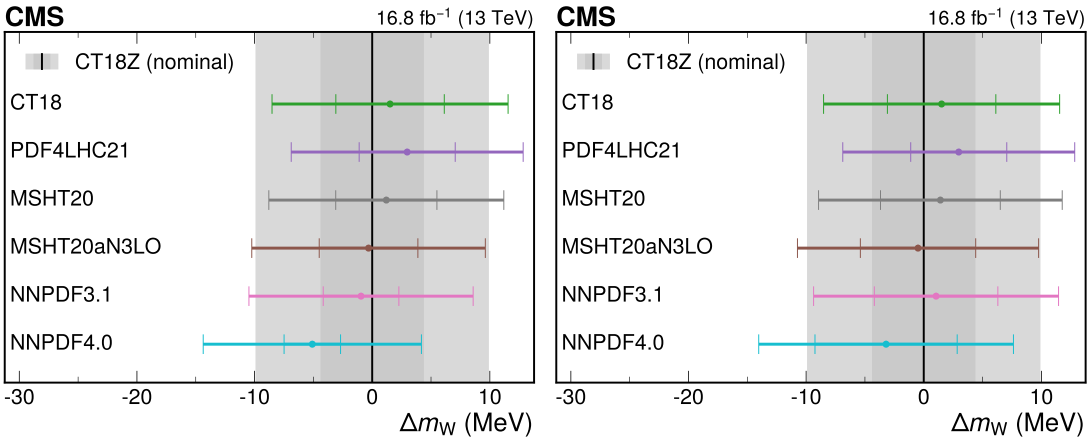

Figure 7: Difference in m_W values for six alternative recent PDF sets, when using the original uncertainty for the given set (left) and when the uncertainties are scaled to accommodate the central prediction of the other sets (right). Each point corresponds to the result obtained when using the indicated PDF set and its uncertainty for the simulated predictions. The inner bar shows the uncertainty from the PDF and the outer bar the total uncertainty. The nominal result, using CT18Z, is shown by the black line, with the CT18Z PDF and total uncertainty shown in dark and light gray, respectively. The uncertainty scaling procedure described in Section of 8 the Methods section in the main work improves the consistency of the m_W values across the PDF sets and with the nominal result.

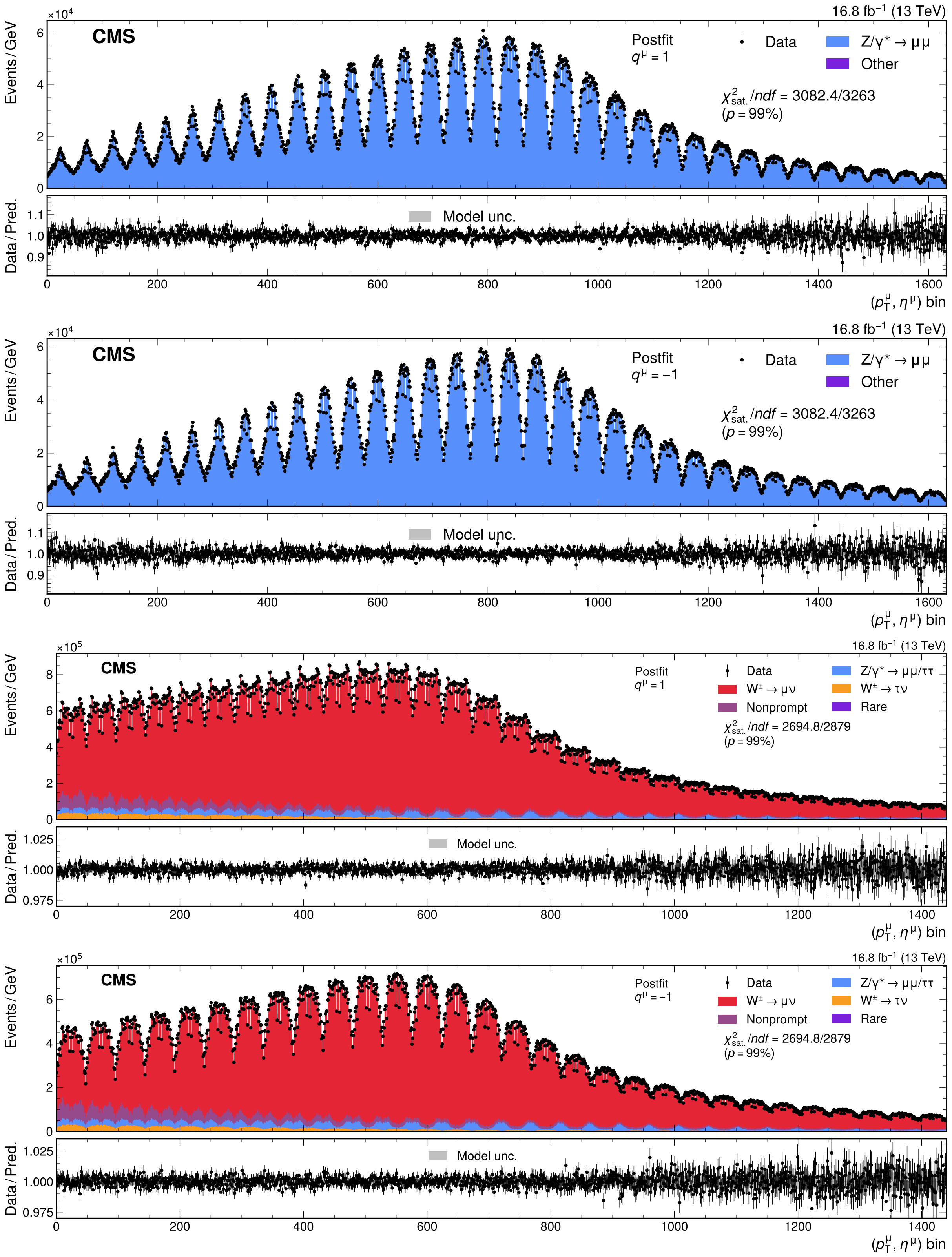

Figure 8: The postfit $(p^{\mu}_{\text{T}}, \eta^{\mu})$ distribution compared to the observed data for the W-like m_{Z} (upper two) and m_{W} (lower two) measurements for positively (upper and second from bottom) and negatively (second from top and lower) charged muons. The predictions and their uncertainties are adjusted to the best fit values obtained from the maximum likelihood fit. The two-dimensional distribution is "unrolled" such that each bin on the x-axis represents one $(p^{\mu}_{\text{T}}, \eta^{\mu})$ cell. The gray band represents the uncertainty in the prediction, before the fit to the data. The bottom panel shows the ratio of the number of events observed in data to the nominal prediction. The vertical bars represent the statistical uncertainties in the data.

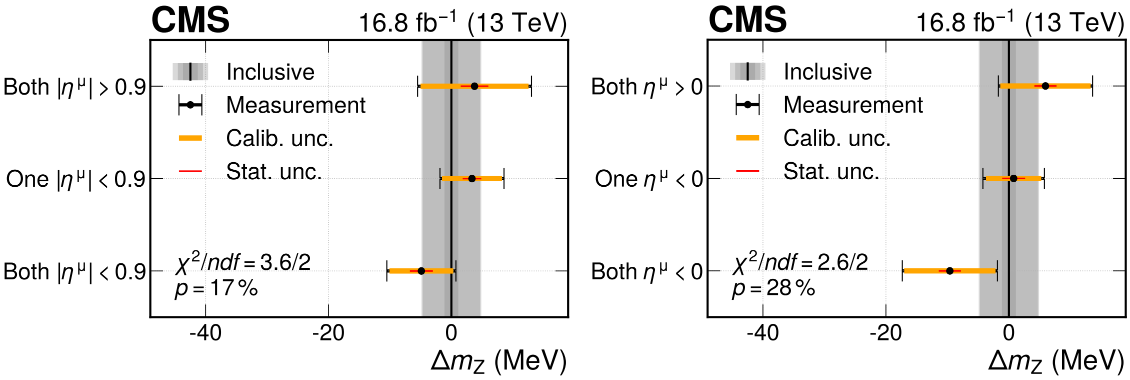

Figure 9: The difference between the nominal m_Z value measured from the $Z \rightarrow \mu\mu$ events and the result when m_Z is allowed to vary, in three regions of the η^μ of the two muons. The results binned in $|\eta^\mu|$ (both central, one central and one forward, and both forward) are shown on the left and results binned in η^μ (both negative, one positive and one negative, and both positive) are shown on the right. The result of a fit with three m_Z parameters is compared with the result with a single m_Z parameter and the compatibility of the results is also shown, as assessed via the saturated goodness-of-fit test. The points show the m_Z result for the indicated η^μ region and the horizontal bars represent the calibration (orange line), statistical (red line), and total (black line) uncertainties. The black vertical line represents the result with a single m_Z parameter, with the three shaded gray bands representing the statistical (dark grey), calibration (intermediate grey), and total (light grey) uncertainties.

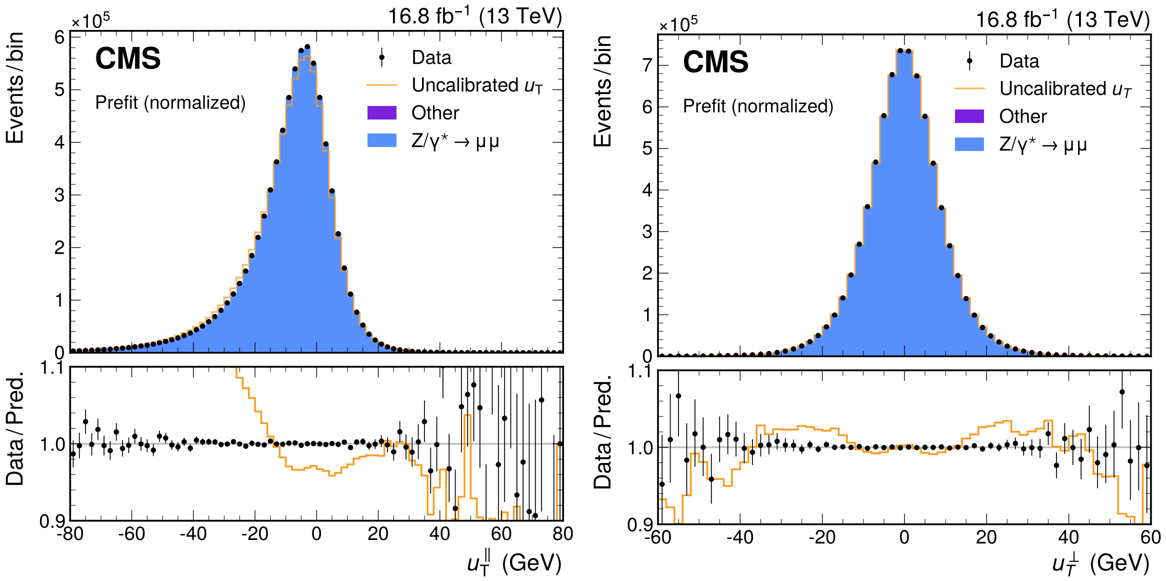

Figure 10: Comparison of the prediction and observed data for the parallel ($u_T^{\parallel}$, left) and perpendicular ($u_T^{\perp}$, right) components of the hadronic recoil. The filled histograms show the simulation with the hadronic recoil corrected according to the procedure described in the text. The orange line shows the predicted distribution before the hadronic recoil corrections. The uncertainties in the predictions are not shown. The bottom panel shows the ratio of the number of events observed in data (black point) and the uncorrected prediction (orange line) to the recoil-corrected prediction.

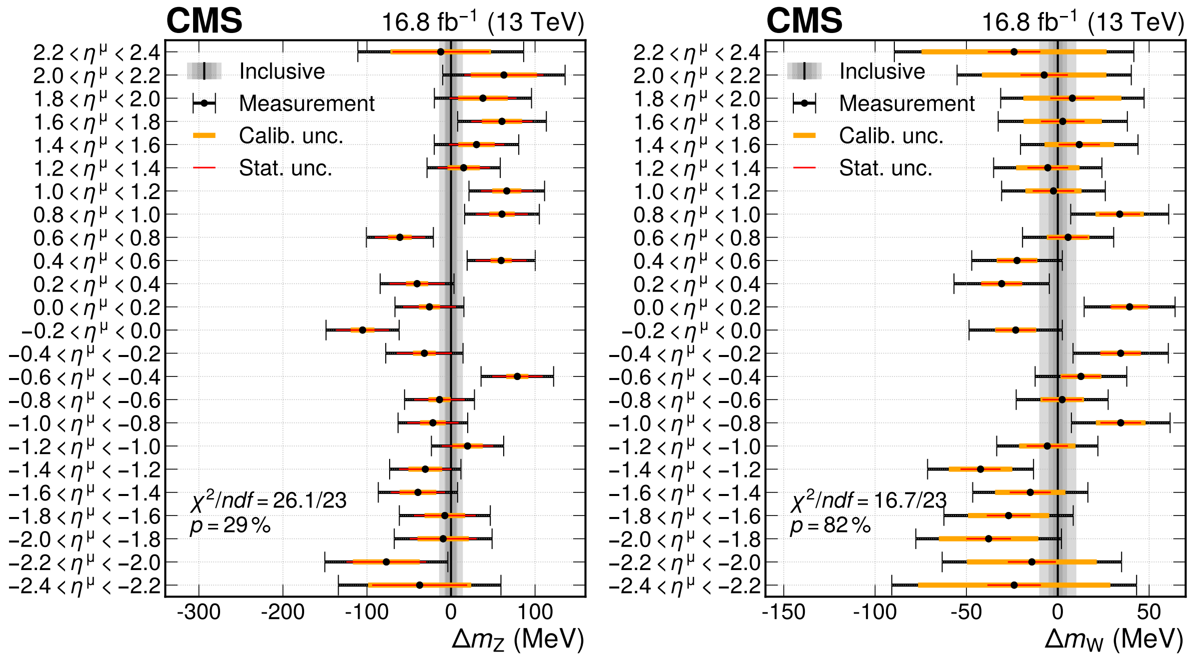

Figure 11: For the W-like m_Z analysis (left) and the m_W measurement (right) the result of a fit with 24 m_V parameters corresponding to different η^μ ranges is compared with the nominal m_V fit result. The χ^2-like compatibility of the two fits is also shown, assessed via the saturated goodness-of-fit test. The points show m_V result for the indicated η^μ region, and the horizontal bars represent the calibration (orange line), statistical (red line), and total (black line) uncertainties. The black vertical line shows the result with a single m_V parameter, with the shaded gray bands representing its statistical, calibration, and total uncertainties.

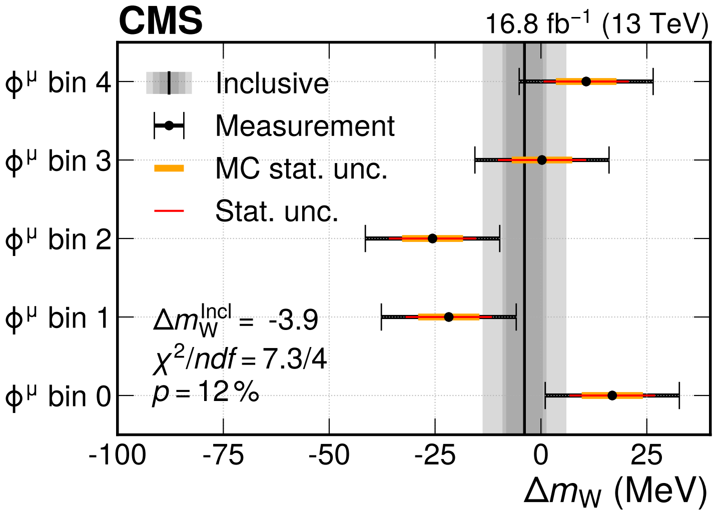

Figure 12: Measured value of m_W after splitting the analyzed data and simulated samples in five uniformly spaced bins of muon ϕ^{μ} from $-\pi$ to π. The points show the m_W measurement for the indicated bin, and the horizontal bars represent the MC (orange line), data statistical (red line), and total (black line) uncertainties. The χ^2-like compatibility of the measurements is also shown, assessed via the saturated goodness-of-fit test. The mutual correlation of the five measurements is accounted for in the χ^2, and is about 30% accounting for the common theoretical uncertainties. Most of the experimental uncertainties are uncorrelated across the five bins. The black vertical line shows the combined result from a simultaneous fit of the five bins with a single m_W parameter, with the shaded gray bands representing its data or MC statistical uncertainty and the total uncertainty. The zero of the horizontal axis corresponds to the nominal measured value. The partial uncertainties are defined using the "global" impacts.

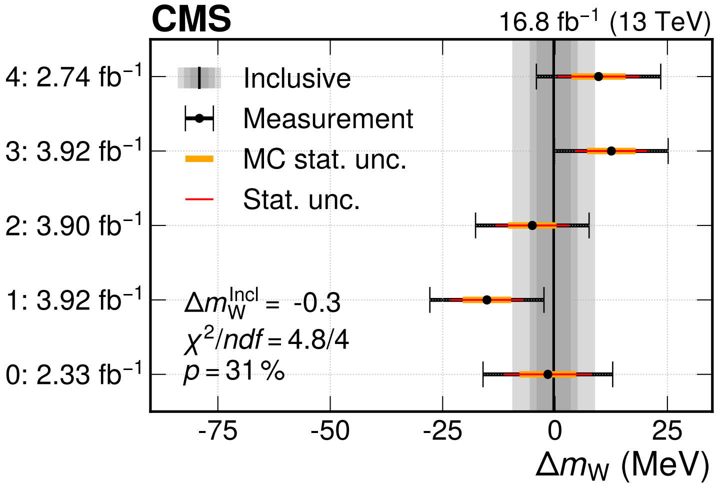

Figure 13: Measured value of m_W after splitting the analyzed data and simulated samples in five independent subsets. The points show the m_W measurement for the indicated integrated luminosity, and the horizontal bars represent the MC (orange line), data statistical (red line), and total (black line) uncertainties. The integrated luminosity of each bin follows the discrete pattern of the data-taking runs. The five bins gather data collected from the beginning to the end of the data taking from bottom to top. Since the average pileup increased with time during 2016, this splitting approximately corresponds to a categorization in bins of pileup as well. The χ^2-like compatibility of the measurements is also shown, assessed via the saturated goodness-of-fit test. The mutual correlation of the five measurements is accounted for in the χ^2, and is about 30% accounting for the common theoretical uncertainties. Most of the experimental uncertainties are treated as uncorrelated across the five bins. The black vertical line shows the combined result from a simultaneous fit of the five bins with a single m_W parameter, with the shaded gray bands representing its data or MC statistical uncertainty and the total uncertainty. The zero of the horizontal axis corresponds to the nominal measured value. The partial uncertainties are defined using the “global” impacts.

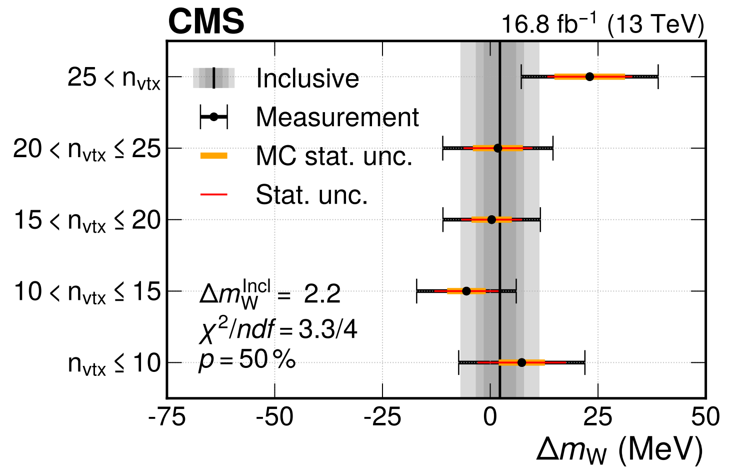

Figure 14: Measured value of m_W after splitting the analyzed data and simulated samples in five independent subsets based on the number of reconstructed vertices (n_{vtx}). The points show the m_W measurement for the indicated n_{vtx}region, and the horizontal bars represent the MC (orange line), data statistical (red line), and total (black line) uncertainties. The χ^2-like compatibility of the measurements is also shown, assessed via the saturated goodness-of-fit test. The mutual correlation of the five measurements is accounted for in the χ^2, and is about 30%, accounting for the common theoretical uncertainties. The black vertical line shows the combined result from a simultaneous fit of the five bins with a single m_W parameter, with the shaded gray bands representing its data or MC statistical uncertainty and the total uncertainty. The zero of the horizontal axis corresponds to the nominal measured value, $m_W = 80\,360.2$ MeV. The partial uncertainties are defined using the "global" impacts.

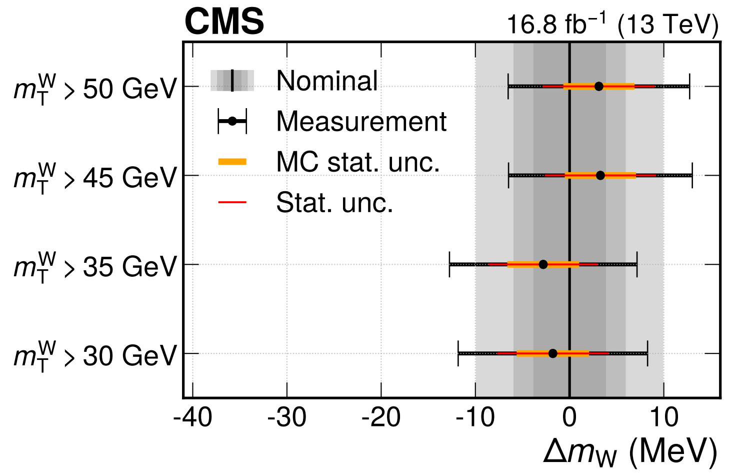

Figure 15: Measured value of m_W after modifying the threshold in the transverse mass m_T. The points show the m_W measurement for the indicated threshold, and the horizontal bars represent the MC (orange line), data statistical (red line), and total (black line) uncertainties. The partial uncertainties are defined using the "global" impacts. The black vertical line shows the nominal measured value, for which the m_T threshold is 40 GeV. The measurements are not statistically independent.

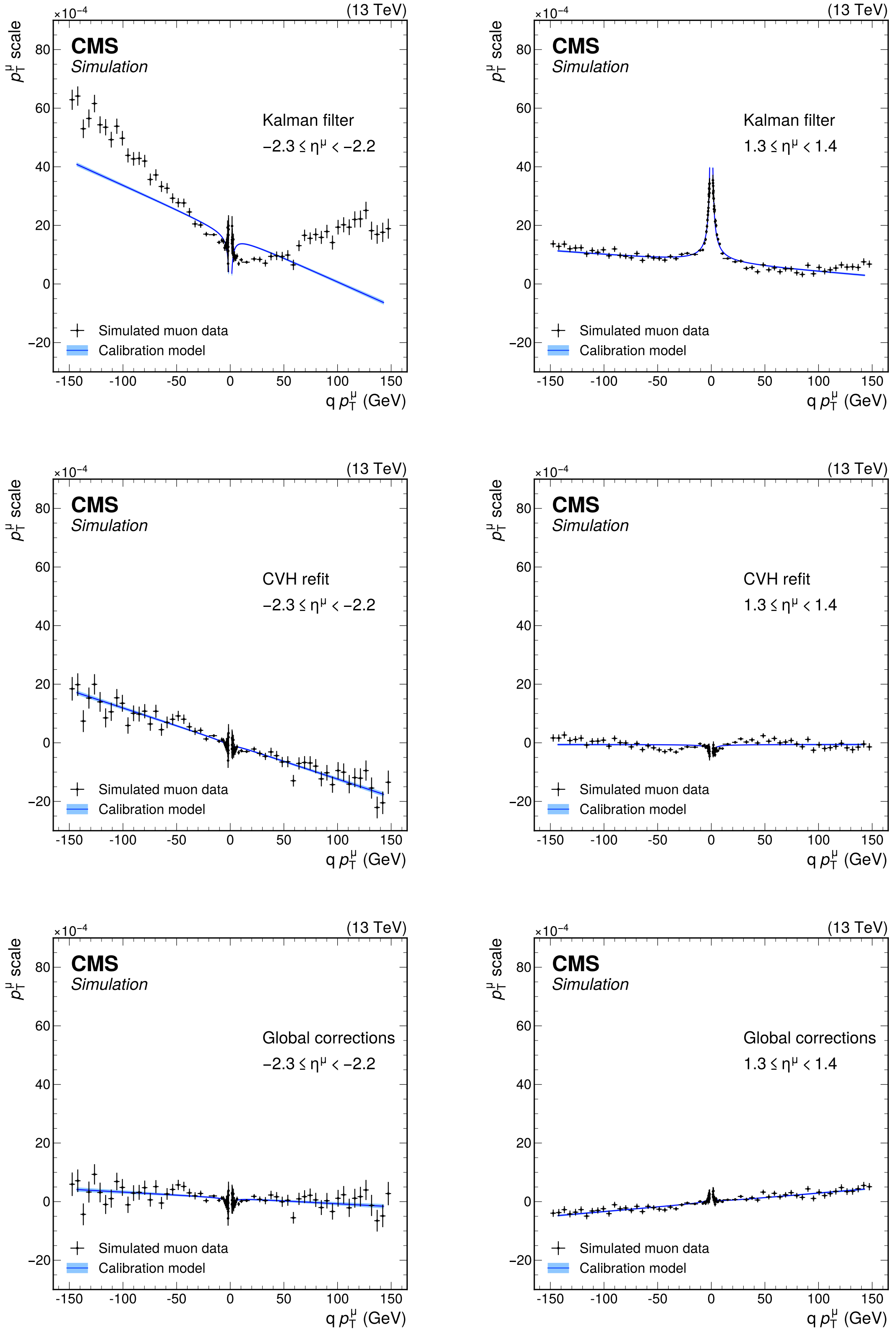

Figure 16: Muon momentum scale bias evaluated in simulated events as a function of p_{T}^{μ} times the muon charge q. The black dots represent simulated data, while the solid line is a fit of the calibration model. The bias is shown after the Kalman filter track fit (top), the CVH refit (middle), and the generalized global corrections applied on top of the CVH refit (bottom). The comparison is performed in two η^{μ} bins in the forward (left) and central (right) regions of the tracker.

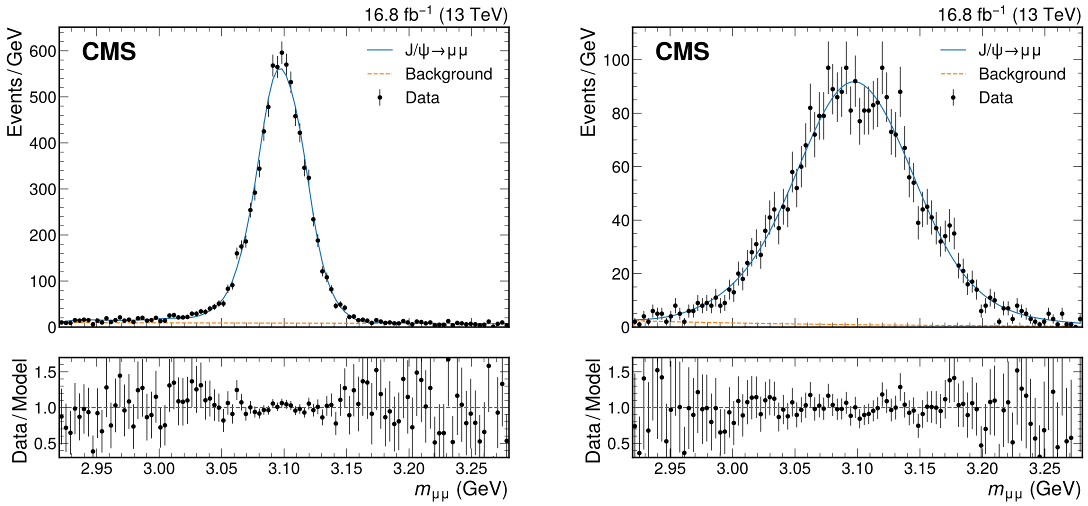

Figure 17: Dimuon invariant mass distributions in $J/\psi \rightarrow \mu\mu$ decays reconstructed in data (black points) in two representative η^{μ} bins in the central (left) and forward (right) regions of the tracker. The blue line represents a fit to the distribution. The small background component is shown as a dashed orange line.

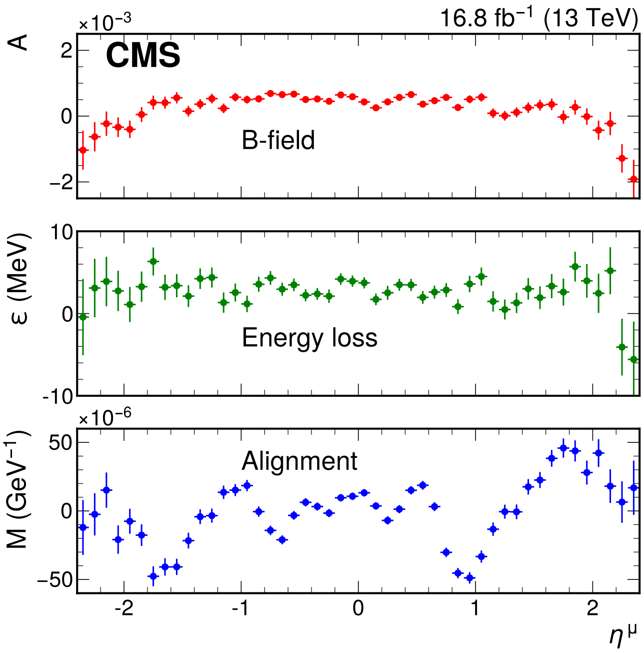

Figure 18: Parameters of the calibration model as functions of η^{μ}, extracted from $J/\psi \rightarrow \mu\mu$ events.

Table 2: Number of nuisance parameters for the main groups of systematic uncertainties, for the W-like m_Z and m_W fits. The number of parameters is displayed only once when it is the same for both fits, while "—" means that this source is not relevant. For completeness, subgroups of parameters are also reported as indented labels for a few groups.

Systematic uncertainties	W-like m_Z	m_W
Muon efficiency	3127	3658
Muon eff. veto	—	531
Muon eff. syst.	343	
Muon eff. stat.	2784	
Nonprompt-muon background	—	387
Prompt-muon background	2	3
Muon momentum scale	314	
L1 prefiring	14	
Integrated luminosity	1	
PDF (CT18Z)	60	
Angular coefficients	177	353
W MiNNLO$_{PS}$ μ_F, μ_R	—	176
Z MiNNLO$_{PS}$ μ_F, μ_R	176	
PYTHIA shower k_T	1	
p_T^V modeling	22	32
Nonperturbative	4	10
Perturbative	4	8
Theory nuisance parameters	10	
c, b quark mass	4	
Higher-order EW	6	7
Z boson width	1	
Z boson mass	—	1
W boson width	—	1
$\sin^2\theta_W$	1	
Total	3725	4833

Table 3: Uncertainties in the W-like m_Z and m_W measurements, comparing the mass difference between charges and the nominal charge combination, using nominal (upper) and global (lower) impacts.

Source of uncertainty	Nominal impact (MeV)			
	in $m_{Z^+} - m_{Z^-}$	in m_Z	in $m_{W^+} - m_{W^-}$	in m_W
Muon momentum calibration	23.1	5.6	21.6	4.8
Muon reco. efficiency	7.1	3.8	7.2	3.0
W and Z angular coeffs.	14.5	4.9	18.7	3.3
Higher-order EW	0.2	2.2	1.5	2.0
p_T^V modeling	0.6	1.7	7.4	2.0
PDF	0.9	2.4	11.8	4.4
Nonprompt-muon background	—	—	7.5	3.2
Integrated luminosity	<0.1	0.3	0.1	0.1
MC sample size	4.9	2.5	3.0	1.5
Data sample size	13.9	6.9	4.7	2.4
Total uncertainty	32.5	13.5	30.3	9.9

Source of uncertainty	Global impact (MeV)			
	in $m_{Z^+} - m_{Z^-}$	in m_Z	in $m_{W+} - m_{W-}$	in m_W
Muon momentum scale	21.2	5.3	20.0	4.4
Muon reco. efficiency	6.5	3.0	5.8	2.3
W and Z angular coeffs.	13.9	4.5	13.7	3.0
Higher-order EW	0.2	2.2	1.5	1.9
p_T^V modeling	0.4	1.0	2.7	0.8
PDF	0.7	1.9	4.2	2.8
Nonprompt-muon background	—	—	4.8	1.7
Integrated luminosity	<0.1	0.2	0.1	0.1
MC sample size	6.4	3.6	8.4	3.8
Data sample size	18.1	10.1	13.4	6.0
Total uncertainty	32.5	13.5	30.3	9.9
